# The discovery of a new nonbile acid modulator of Takeda G protein‐coupled receptor 5: An integrated computational approach

**DOI:** 10.1002/ardp.202400423

**Published:** 2025-01-13

**Authors:** Rudy Salam, Michael Bakker, Mária Krutáková, Alžbeta Štefela, Petr Pávek, Jurjen Duintjer Tebbens, Jan Zitko

**Affiliations:** ^1^ Department of Biophysics and Physical Chemistry, Faculty of Pharmacy Charles University Hradec Králové Czech Republic; ^2^ Department of Pharmacy, Faculty of Medicine Universitas Brawijaya Malang Indonesia; ^3^ Department of Pharmacology and Toxicology, Faculty of Pharmacy Charles University Hradec Králové Czech Republic; ^4^ Department of Pharmaceutical Chemistry and Pharmaceutical Analysis, Faculty of Pharmacy Charles University Hradec Králové Czech Republic

**Keywords:** INT‐777, molecular docking, nonbile acid, pharmacophore, TGR5

## Abstract

The Takeda G protein‐coupled receptor 5 (TGR5), also known as GPBAR1 (G protein‐coupled bile acid receptor), is a membrane‐type bile acid receptor that regulates blood glucose levels and energy expenditure. These essential functions make TGR5 a promising target for the treatment of type 2 diabetes and metabolic disorders. Currently, most research on developing TGR5 agonists focuses on modifying the structure of bile acids, which are the endogenous ligands of TGR5. However, TGR5 agonists with nonsteroidal structures have not been widely explored. This study aimed at discovering new TGR5 agonists using bile acid derivatives as a basis for a computational approach. We applied a combination of pharmacophore‐based, molecular docking, and molecular dynamic (MD) simulation to identify potential compounds as new TGR5 agonists. Through pharmacophore screening and molecular docking, we identified 41 candidate compounds. From these, five candidates were selected based on criteria including pharmacophore features, a docking score of less than 9.2 kcal/mol, and similarity in essential interaction patterns with a reference ligand. Biological assays of the five hits confirmed that Hit‐3 activates TGR5 similarly to the bile acid control. This was supported by MD simulation results, which indicated that a hydrogen bond interaction with Tyr240 is involved in TGR5 activation. Hit‐3 (CSC089939231) represents a new nonsteroidal lead that can be further optimized to design potent TGR5 agonists.

## Graphical abstract

A high‐throughput virtual screening utilizing pharmacophore search and molecular docking discovered new nonsteroidal ligands of Takeda G protein‐coupled receptor 5 (TGR5). Activation of TGR5 by Hit‐3 (CSC089939231) was verified experimentally and rationalized by molecular dynamics simulations. Hit‐3 can serve as a useful starting point for future hit‐to‐lead structure optimization.

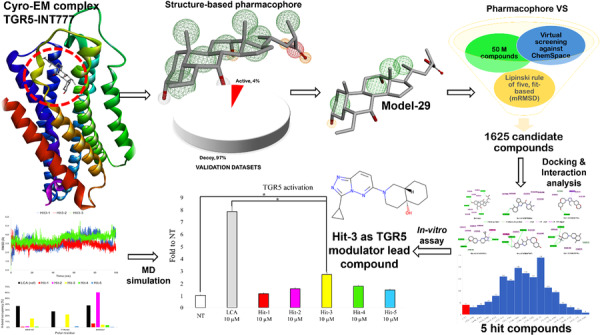

## 1 INTRODUCTION

The Takeda G protein‐coupled receptor 5 (TGR5), also known as GPBAR1 (UniProt ID: Q8TDU6), is a membrane‐type bile acid receptor (M‐BAR) and is a member of the rhodopsin‐like superfamily of G‐protein‐coupled receptors.^[^ ^1^, ^2^ ^]^ This receptor is widely expressed across all tissues, including the gallbladder, placenta, spleen, lung, liver, intestine, kidney, adrenal glands, adipose tissue, and smooth muscle, as well as in immune cells, monocytes, and macrophages.^[^ ^2^, ^3^ ^]^ TGR5 regulates metabolism via the bile acid (BA) signaling pathway and contributes to various pathophysiological processes.^[^ ^4^ ^]^ TGR5 activation activates intracellular adenylyl cyclase cyclic adenosine 3′,5′‐monophosphate (cAMP) signaling pathways and the protein kinase‐A pathway and profoundly affects bile acid metabolism, blood sugar, and energy intake and inhibits macrophage activation by proinflammatory mediators.^[^ ^2^, ^3^, ^5^, ^6^ ^]^ Through the TGR5–GRK–arrestin axis, TGR5 activation can impair nuclear factor kappa‐light (NF‐κB) transcriptional activity and turn on SRC kinase (proto‐oncogene tyrosine‐protein kinase Src), resulting in tyrosine phosphorylation of several antiviral signaling molecules and therefore modulating the innate antiviral immune response.^[^ ^3^, ^7^ ^]^ An exhaustive literature review has confirmed that the major functions of TGR5 are linked to blood glucose homeostasis and energy expenditure.^[^ ^3^, ^8^, ^9^ ^]^ TGR5 activation promotes the release of glucagon‐like peptide‐1 (GLP‐1), which amplifies insulin secretion and improves blood glucose homeostasis, thus making TGR5 a potential target for type 2 diabetes therapy.^[^ ^2^, ^3^, ^10^ ^]^

**Figure 1:**
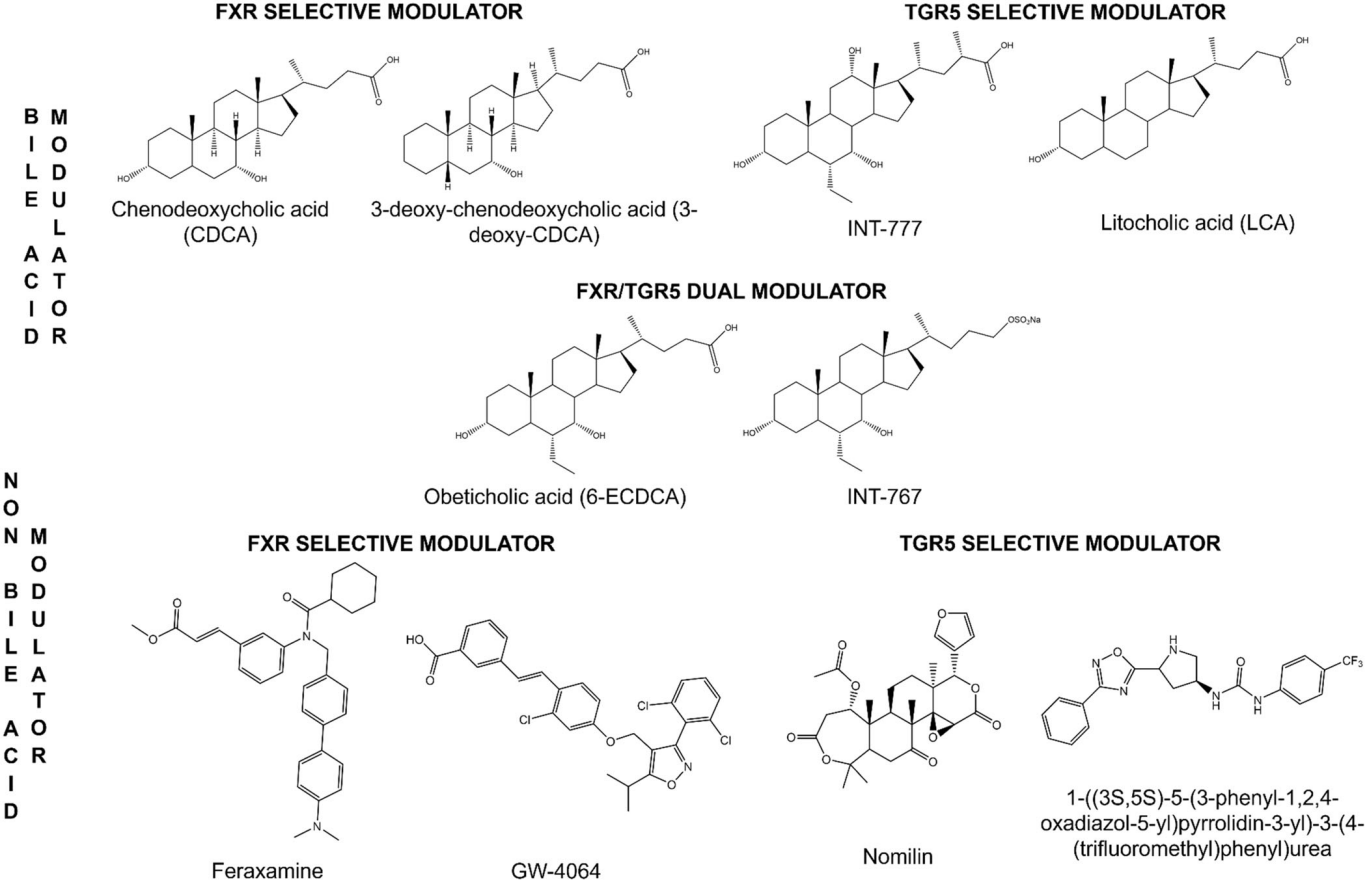
Bile acid and nonbile acid modulators of farnesoid X receptor (FXR) and Takeda G protein‐coupled receptor 5 (TGR5).^[^ ^1^, ^11^ ^]^

TGR5 can be activated by both steroidal and nonsteroidal ligands.^[^ ^1^, ^11^ ^]^ The steroid group is based on the structure of bile acids and includes both endogenous (lithocholic acid [LCA]) and semi‐synthetic (INT‐777) derivatives (Figure 1).^[^ ^1^, ^11^ ^]^ The nonsteroidal group consists of synthesized small molecular weight compounds such as ((1,2,4‐oxadiazol‐5‐yl)pyrrolidin‐3‐yl))ureydil derivatives (Figure 1, bottom right corner).^[^ ^12^ ^]^ Natively, TGR5 modulators from the steroid class can activate the other bile acid receptor, farnesoid X receptor (FXR).^[^ ^1^ ^]^ Both bile acid receptors can be activated by bile acids and their derivatives; therefore, most efforts in the development of new modulators are focused on the structural modification of bile acids (Figure 1), and this is due to the fact that both receptors have similar structural features in the ligand‐binding site.^[^ ^1^ ^]^ Consideration of the use of dual modulators becomes an interesting strategy; however, their utilization can be associated with severe side effects. Consequently, the development of new selective modulators of TGR5 is challenging.

According to the reported cryo‐electron microscopy structures, the ligand‐binding cavity of TGR5 has an oval shape with hydrophilic residues clustered at the bottom of the cavity, and the rest of the ligand‐binding domain surface is formed primarily by hydrophobic residues.^[^ ^4^, ^13^ ^]^ Inside the TGR5 orthosteric region, seven hydrophobic residues and three polar residues interact to accommodate compounds with different chemical features, which are not restricted to steroidal ligands such as bile acid.^[^ ^4^, ^13^ ^]^ Bile acid, a potent signaling molecule with pleiotropic effects that naturally activate TGR5, binds to TGR5 with its A‐ring facing the hydrophilic bottom of the cavity formed by polar residues Tyr240, Ser270, Glu169, and Asn93.^[^ ^2^, ^3^, ^13^, ^14^ ^]^ The absence of the hydroxyl group at the 3α position is found to be related to the selectivity of bile acids in the activation of TGR5 or FXR. The lack of the 3α‐hydroxyl group does not influence the activation of bile acids with respect to FXR but could decrease the activation response to TGR5 due to the absence of a hydrogen bond (H‐bond) with Tyr240.^[^ ^1^, ^13^ ^]^ Bile acids with longer and branched side chains have more affinity to TGR5 than to FXR.^[^ ^15^, ^16^ ^]^ Bile acids with longer and branched side chains have more affinity to TGR5 than to FXR.^[^ ^15^, ^16^ ^]^ In addition to selectivity challenges, safety issues of bile acid‐derivative ligands such as LCA and chenodeoxycholic acid (CDCA) have become a concern as LCA is known to be a toxic bile acid and CDCA increases the hepatic biomarkers alanine transaminase (ALT) and aspartate aminotransferase (AST).^[^ ^5^ ^]^ Steroid TGR5 agonists have been additionally linked to gallbladder overfilling, gallbladder emptying blockage, itching, and cardiovascular issues, adding to the safety concerns with steroid TGR5 agonists.^[^ ^9^ ^]^

Computer‐aided drug design (CADD), combining theoretical and computational approaches, can be used for the discovery, development, and analysis of both steroidal and nonsteroidal TGR5 agonists. Before sufficient information on the crystal structure of TGR5 was discovered, CADD attempted to overcome the lack of information by exploiting the homology of receptors and pharmacophore modeling. Pharmacophore approaches can be divided into two, ligand‐based and structure‐based, with the latter technique being possible only when information on the three‐dimensional structure of the target is available.^[^ ^17^ ^]^ Ligand‐based pharmacophore and receptor homology were employed by Sindhu to compensate for the lack of the crystal structure of TGR5.^[^ ^5^ ^]^ The unclear binding conformation of the ligand in the binding cavity and the possibility of missing important features in the binding cavity are the common problems of ligand‐based pharmacophores that could influence the accuracy of the virtual screening.^[^ ^17^ ^]^ A recent breakthrough was the discovery of the cryo‐electron microscopy structure of TGR5 by Yang et al. (PDB ID: 7CFN) in 2020 and Ma et al. (PDB ID: 7XTQ) in 2022 with resolutions of 3.0 and 3.2 Å, respectively. The two reported structures are nearly identical, with the exception of the ligand attached to the TGR5 binding cavity.^[^ ^4^, ^13^ ^]^ This discovery has opened a plethora of possibilities for pharmacophore agonist development. Structure‐based pharmacophore modeling is direct and more precise than ligand‐based modeling due to incorporating the important interactions that take place between ligands and receptors when eliciting a biological response.^[^ ^17^, ^18^ ^]^

This study focused on a computational approach with a structure‐based pharmacophore following the discovery of the cryo‐electron microscopy structure of TGR5. The availability of knowledge regarding ligand interactions in the TGR5 binding cavity encourages the construction of new structure‐based pharmacophore models in search of new TGR5 agonists. In the present study, compounds that met the predefined pharmacophore features and attained the best docking score values were analyzed for molecular interactions. The compounds with the best fit to the docking poses and similar interactions to the reference ligand (INT‐777) were identified as hit molecules and considered potential TGR5 agonists. Furthermore, biological assays were performed to confirm the potential activation of TGR5 and rationalize the activity of the hit compounds obtained in the previous step using molecular dynamic (MD) simulations. For safety issues of the hit compounds, we provide estimated data concerning chemical properties and pharmacokinetic parameters such as absorption, distribution, metabolism, excretion (ADME), and toxicity.

## 2 RESULTS AND DISCUSSION

### 2.1 Pharmacophore generation and validation

**Figure 2:**
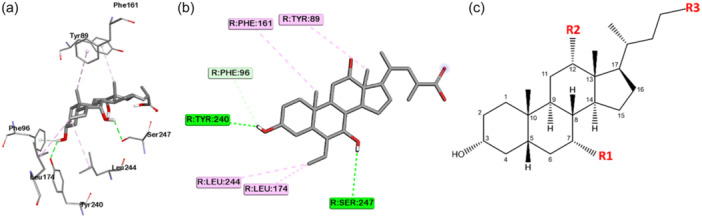
(a, b) Interaction between INT‐777 and TGR5. (c) Core structure of cholic acid with differences in R^1^, R^2^, and R^3^. (R indicates functional group; dashed green line shows hydrogen bond interaction; dashed purple line indicates hydrophobic interaction).

The pharmacophore model was constructed based on the interactions observed in the crystallographic complex of the TGR5 receptor with its semi‐synthetic bile acid agonist INT‐777 (PDB ID: 7CFN).^[^ ^13^ ^]^ Seven residues of TGR5 interact directly with INT‐777, as depicted in Figure 2. Bile acids and their derivatives have the same core structure but different functional groups at C7 (position 7α, R^1^), C12 (position 12α, R^2^), and at the terminal sidechain (R^3^), as demonstrated in Figure 2. The hydroxyl functional groups can efficiently trigger hydrophobic interactions depending on the number and position of the hydroxyl groups.^[^ ^19^ ^]^ The 3α‐hydroxy group forms hydrogen bonds (H‐bonds) with Tyr240 while the 7α‐hydroxy group as R^1^ forms hydrogen bonds with Ser247 and triggers hydrophobic interactions with Leu244.^[^ ^13^ ^]^ A point to be considered is the hydroxyl group at the 3α‐position influences the selectivity of the ligand toward TGR5 compared with other BA receptors such as FXR.^[^ ^1^ ^]^ Therefore, the hydrogen donor and acceptor feature at the 3α‐position are crucial features in pharmacophore construction. The abovementioned H‐bonds can attract the side chains of hydrophobic residues surrounding Ser247 and make them move closer to the ligand, triggering hydrophobic interactions.^[^ ^19^, ^20^ ^]^ In return, these hydrophobic interactions increase the strength of the hydrogen bonds due to stabilizing their geometry.^[^ ^20^ ^]^ Thus, the addition of hydrophobic features to the core structure is beneficial for improving the H‐bond stability of the screened compounds. Moreover, in terms of selectivity toward TGR5, bile acid derivatives with longer and branched side chains have a greater affinity toward TGR5 than FXR,^[^ ^1^ ^]^ which suggests that the addition of hydrophobic features to one of the methyl groups on the side chain could increase the possibility of obtaining more selective compounds.

**Figure 3:**
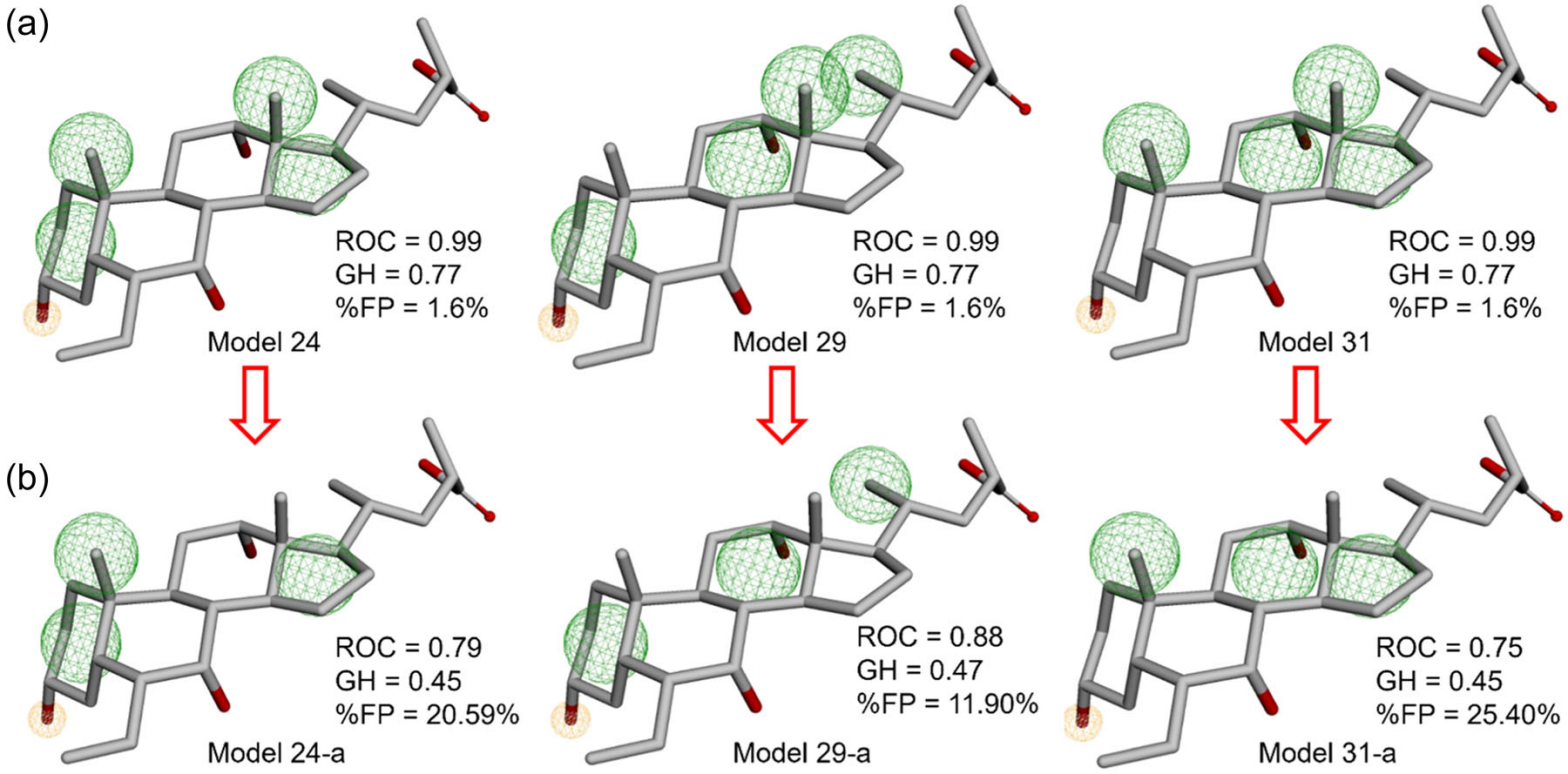
(a) The three‐dimensional (3D) arrangements of the selected pharmacophore models: model‐24, model‐29, and model‐31. (b) The deletion of hydrophobic features in C13 of the three models.

All pharmacophore models were generated using the Pharmit server with INT‐777 and TGR5 as inputs. Seventeen pharmacophore features were retrieved because of feature mapping utilizing a Pharmit server, namely three hydrogen bond donors (D), five hydrogen bond acceptors (A), and nine hydrophobic features (H). An issue frequently encountered for structure‐based pharmacophore modeling is that too many pharmacophore features (generally nonprioritized) can be identified for specific binding sites of macromolecular targets.^[^ ^21^ ^]^ Consequently, the selection of a number of features to generate a hypothetical pharmacophore is a challenging task and typically the pharmacophore has three to seven features.^[^ ^21^ ^]^ According to the information about the crucial interactions related to ligand selectivity toward TGR5, the pharmacophore was set to have six features. The use of six features instead of five features included the consideration that the number of screened compounds would not be too large and to avoid the number of false positives (FPs) obtained when using five features instead of six features (Figure 3). Validation is a crucial step to ensure the generated model can selectively find potential hits for TGR5 agonists in the screening process. A data set of 15 active bile acid‐derived compounds and 437 decoy compounds was used to validate the pharmacophore model (Supporting Information S2: Tables S1 and S2). A total of 430 decoy compounds were acquired from the Database of Useful Decoys (DUD‐E) and combined with seven decoy compounds derived from bile acid derivatives which were previously shown inactive in our in vitro assay. The active and decoy data sets were submitted to the Pharmit server, which generates conformers automatically before the pharmacophore model validation process. The best pharmacophore model was chosen using receiver operating characteristic (ROC) parameters and calculated statistical descriptors. The ROC score is a metric used to evaluate the effectiveness of a predictive model. It evaluates the sensitivity of the model to distinguish between active and decoy and is represented by a score between 0 and 1, with 1 being a perfect score. The higher the ROC score, the better the performance of the model. The GH and E values are utilized as a reference for statistical descriptor calculation; the higher the value, the better the pharmacophore's ability to identify active compounds.^[^ ^22^ ^]^

Each pharmacophore model had six features and was constructed manually using the Pharmit server (Supporting Information S2: Table S3). The top 10 best pharmacophore models with ROC scores ranging from 0.93 to 0.99 were identified from a total of 48 constructed models (Table 1). The sensitivity value of 1.00 and the ROC value of 0.99, in this perspective, indicate that five pharmacophore models are highly accurate in distinguishing between active and decoy compounds. Model‐28 and model‐33 had a sensitivity of 1.00 and an ROC of 0.99; however, these models were excluded due to lower E and GH values (19.65 and 0.75) than the other three models, model‐24, model‐29, and model 31. Since model‐24, model‐29, and model‐31 have the same sensitivity and selectivity and only one model was used in pharmacophore screening, the three models were re‐evaluated with slight modifications. The three models were further modified by removing the hydrophobic feature on C13 to determine the model to be utilized in the intended screening process. The elimination of this feature was based on the fact that the methyl group on C13 is not directly involved in crucial interactions with TGR5, as well as the similarity of the features of the three selected models. Additionally, the three updated models were re‐evaluated to determine their selectivity and specificity (Figure 3).

**Table 1:** Top 10 pharmacophore models were generated using the Pharmit server.^[^ ^23^ ^]^

Model	Features	TP	TN	FP	FN	Sensitivity	Specificity	ROC score	D	A	Ht	Ha	%YA	%RA	E	GH
Model‐4	ADHHHH	13	431	6	2	0.87	0.99	0.93	452	15	19	13	0.68	0.87	20.62	0.74
Model‐5	ADHHHH	13	431	6	2	0.87	0.99	0.93	452	15	19	13	0.68	0.87	20.62	0.74
Model‐24	ADHHHH	15	430	7	0	1.00	0.98	0.99	452	15	22	15	0.68	1.00	20.55	0.77
Model‐27	ADHHHH	13	431	6	2	0.87	0.99	0.93	452	15	19	13	0.68	0.87	20.62	0.74
Model‐28	ADHHHH	15	429	8	0	1.00	0.98	0.99	452	15	23	15	0.65	1.00	19.65	0.75
Model‐29	ADHHHH	15	430	7	0	1.00	0.98	0.99	452	15	22	15	0.68	1.00	20.55	0.77
Model‐30	ADHHHH	14	431	6	1	0.93	0.99	0.96	452	15	20	14	0.70	0.93	21.09	0.77
Model‐31	ADHHHH	15	430	7	0	1.00	0.98	0.99	452	15	22	15	0.68	1.00	20.55	0.77
Model‐32	ADHHHH	13	431	6	2	0.87	0.99	0.93	452	15	19	13	0.68	0.87	20.62	0.74
Model‐33	ADHHHH	15	429	8	0	1.00	0.98	0.99	452	15	23	15	0.65	1.00	19.65	0.75

*Note*: ROC analysis.

Sensitivity = TP/(TP + FN);

Specificity = TN/(TN + FP);

where: TP, true positives (actives); TN, true negatives (inactive); FN, false negatives (actives that are missed by the pharmacophore model); FP, false positives (inactive that are retrieved by the model as actives).

Güner–Henry (GH) scoring.

Total number of compounds in the test set (D); Number of actives in the test set (A); The number of actives retrieved by the model (Ha); Total number of hits retrieved by the model (Ht).

The percent yield of actives (% YA) = (Ha/Ht) × 100; measures the selectivity of generated pharmacophore models.

The percent ratio of actives (% RA) = (Ha/A) × 100; represents the coverage of activity space by the generated pharmacophore models.

False positives (FP) = Ht−Ha.

False negatives (FN) = A−Ha.

The enrichment factor (E) = (Ha × D)/(Ht × A); reflects the power of a pharmacophore model to identify active compounds in the test set relative to random screening.

The goodness of hits $GH\;=\;\left(\frac{Ha\left(3A+Ht\right)}{4HtA}\right)\left(1-\frac{Ht-Ha}{D-A}\right)$.

(GH) represents the ability of a pharmacophore model to differentiate and isolate active compounds from the rest in the test set.

The modified model 24‐a and model‐31‐a (Figure 3) showed a notable increase in FP values characterized by the retrieval of decoy compounds from 1.6% (6 features) to 20.59% and 25.40%, respectively, employing five features. Model 29‐a (Figure 3b) enhanced decoy compound retrieval from 1.6% (six features) to 11.90% (five features), resulting in the modified pharmacophore model with the lowest increase in FP value compared with the previous two models. These results indicated that model‐29 had higher selectivity and specificity than the other two models (model‐24 and model‐31) after modification. Therefore, model‐29 (six features) was selected for pharmacophore‐based virtual screening.

### 2.2 Pharmacophore‐based virtual screening

Model‐29 was selected and applied to pharmacophore‐based virtual screening with ChemSpace, a database containing 50 million compounds. To ensure reliable results, ligand/receptor shape filters, Lipinski rule, number of H‐bond donors ≤5, number of H‐bond acceptors ≤10, molecular weight ≤500, and logP <5.0 were applied. All candidate compounds needed to contain all the model's pharmacophore features and be within the tolerance range. The ChemSpace database screening yielded 3057 candidate compounds, which continued with pan‐assay interference compounds (PAINS) filtering and removal of duplicate compounds followed by filtering using the fit value of minimized root‐mean‐square deviation (mRMSD) with a maximum value limit of 2 Ǻ, resulting in 1625 candidate compounds (Supporting Information S2: File 1) for further docking‐based virtual screening.

### 2.3 Docking‐based virtual screening

Docking validation is essential to evaluate the accuracy of the utilized docking method before performing docking‐based virtual screening. To validate the method, the cocrystallized ligand (INT‐777, PDB: 7CFN) was redocked in the binding cavity. Remarkably, the redocked ligand pose overlapped with the cocrystallized ligand pose (Supporting Information S2: Figure S1) with an RMSD value of 1.780 Ǻ, which is considered to be successful according to the criteria of the RMSD value range of 1.5–2.0.^[^ ^24^ ^]^ LCA, an endogenous bile acid further used in the biological assay as the positive control, was docked to a comparable pose and had similar molecular interactions to the active site of TGR5 compared with INT‐777 (Supporting Information S2: Figure S2).

**Figure 4:**
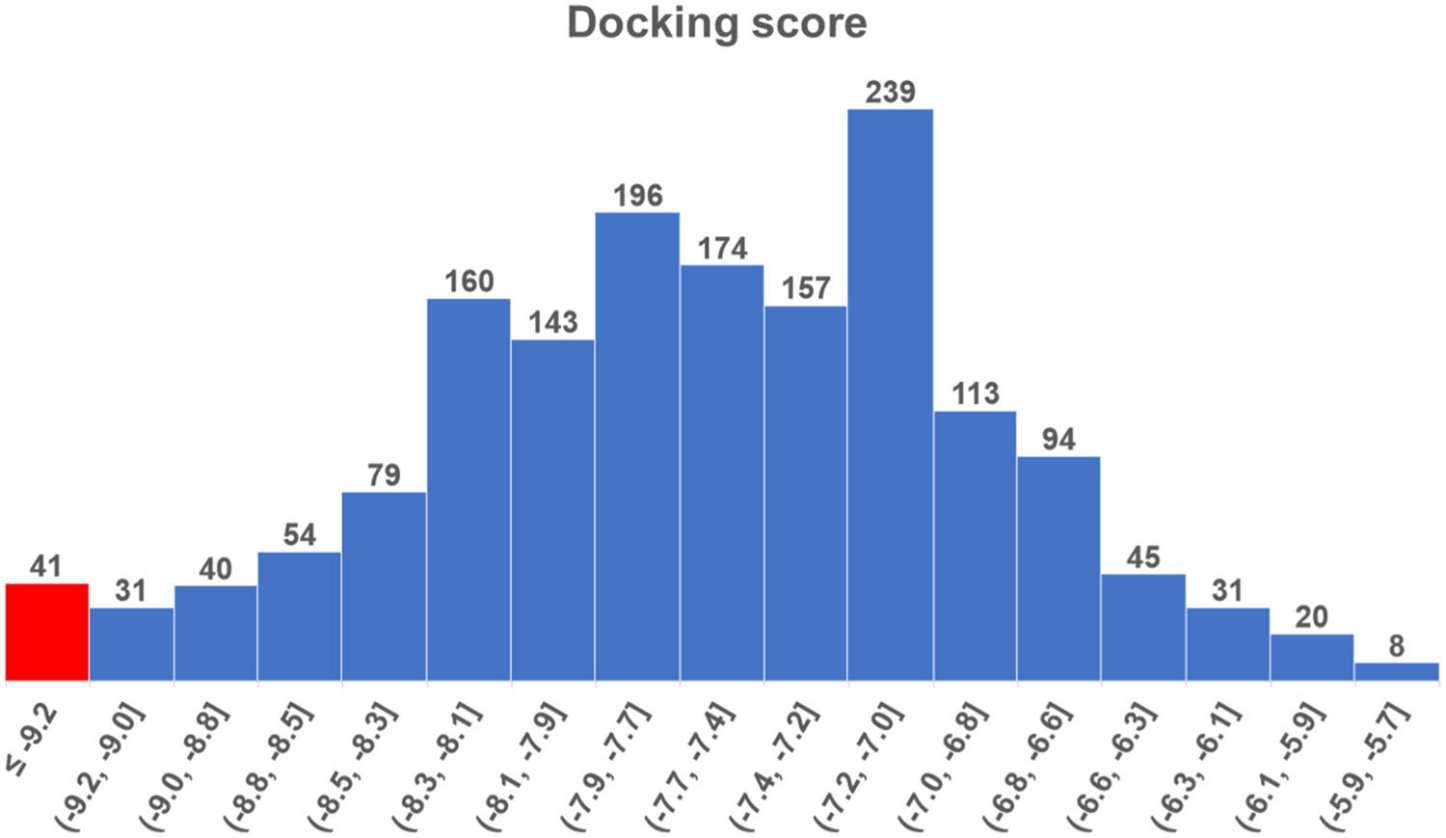
Docking score distribution histogram of 1625 candidate molecules. The red box indicates compounds with equal or better (lower) values of docking score compared to the reference ligand INT‐777.

Subsequently, all 1625 candidate compounds from pharmacophore screening (Supporting Information S2: File 1) were docked in the TGR5 binding cavity using Autodock Vina.^[^ ^25^ ^]^ The docking score of the docked candidate compounds ranged from –10.2 to –5.2 kcal/mol (Figure 4). Forty‐one candidate compounds with lower or equal docking score to INT‐777, a reference ligand with a docking score of –9.2 kcal/mol, were subjected to the second round of docking with increasing exhaustiveness parameter to 32 from the default value of 8 (Figure 4, red box, and Table 2).

**Table 2:** The docking score of the top 41 ligands after docking‐based virtual screening (1st round of docking).

No.	Candidate Compound ID	Docking score (kcal/mol)	No.	Candidate Compound	Docking score (kcal/mol)
1	CSC077963399	–10.1	22	CSC080644454	–9.4
2	CSC079705462	–10.1	23	CSC089939231^a^ (Hit‐3)	–9.4
3	CSC094839014	–10.0	24	CSC058505516	–9.3
4	CSC081416547	–9.9	25	CSC060356222	–9.3
5	CSC101843480	–9.9	26	CSC063236646	–9.3
6	CSC078440051	–9.8	27	CSC073382977	–9.3
7	CSC085237134	–9.8	28	CSC083671887^a^ (Hit‐4)	–9.3
8	CSC058170742	–9.7	29	CSC085298700^a^ (Hit‐5)	–9.3
9	CSC058466881	–9.7	30	CSC085766882	–9.3
10	CSC063325620	–9.7	31	CSC101124650	–9.3
11	CSC088670089	–9.7	32	CSC047179274	–9.2
12	CSC057935886^a^ (Hit‐1)	–9.6	33	CSC056736927	–9.2
13	CSC101141357	–9.6	34	CSC058627952	–9.2
14	CSC067091425	–9.5	35	CSC066721915	–9.2
15	CSC079339708	–9.5	36	CSC069126668	–9.2
16	CSC081262642	–9.5	37	CSC076083544	–9.2
17	CSC081667704^a^ (Hit‐2)	–9.5	38	CSC089109147	–9.2
18	CSC092266505	–9.5	39	CSC093730211	–9.2
19	CSC056910159	–9.4	40	CSC099558229	–9.2
20	CSC077830976	–9.4	41	CSC099828047	–9.2
21	CSC079366454	–9.4			

a Compounds selected in the 2nd round of docking based on visual inspection of interactions to TGR5, further denoted as Hit‐1 to Hit‐5.

The second round of molecular docking studies was performed to determine the hit compounds with similar interaction patterns as the reference ligand INT777. Of the 41 candidates analyzed for interactions, five compounds (denoted by “a” in Table 2) were selected as hit compounds and advanced to the TGR5 activation assay.

**Figure 5:**
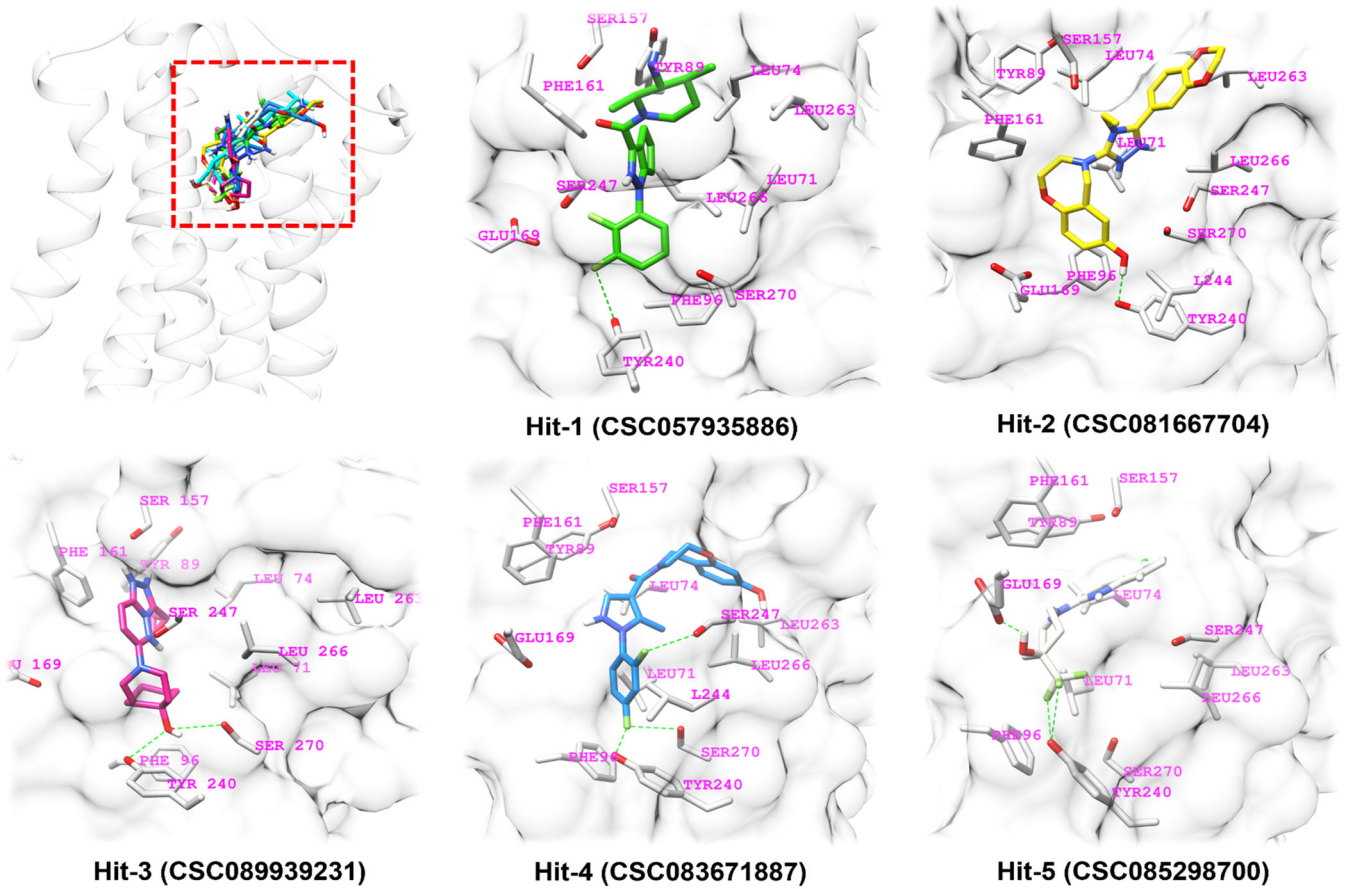
Best poses of the five hit compounds in the ligand‐binding domain (LBD) of Takeda G protein‐coupled receptor 5 (TGR5) (the top left corner image of all hits on the LBD of TGR5; dashed green line shows hydrogen bond interaction).

**Figure 6:**
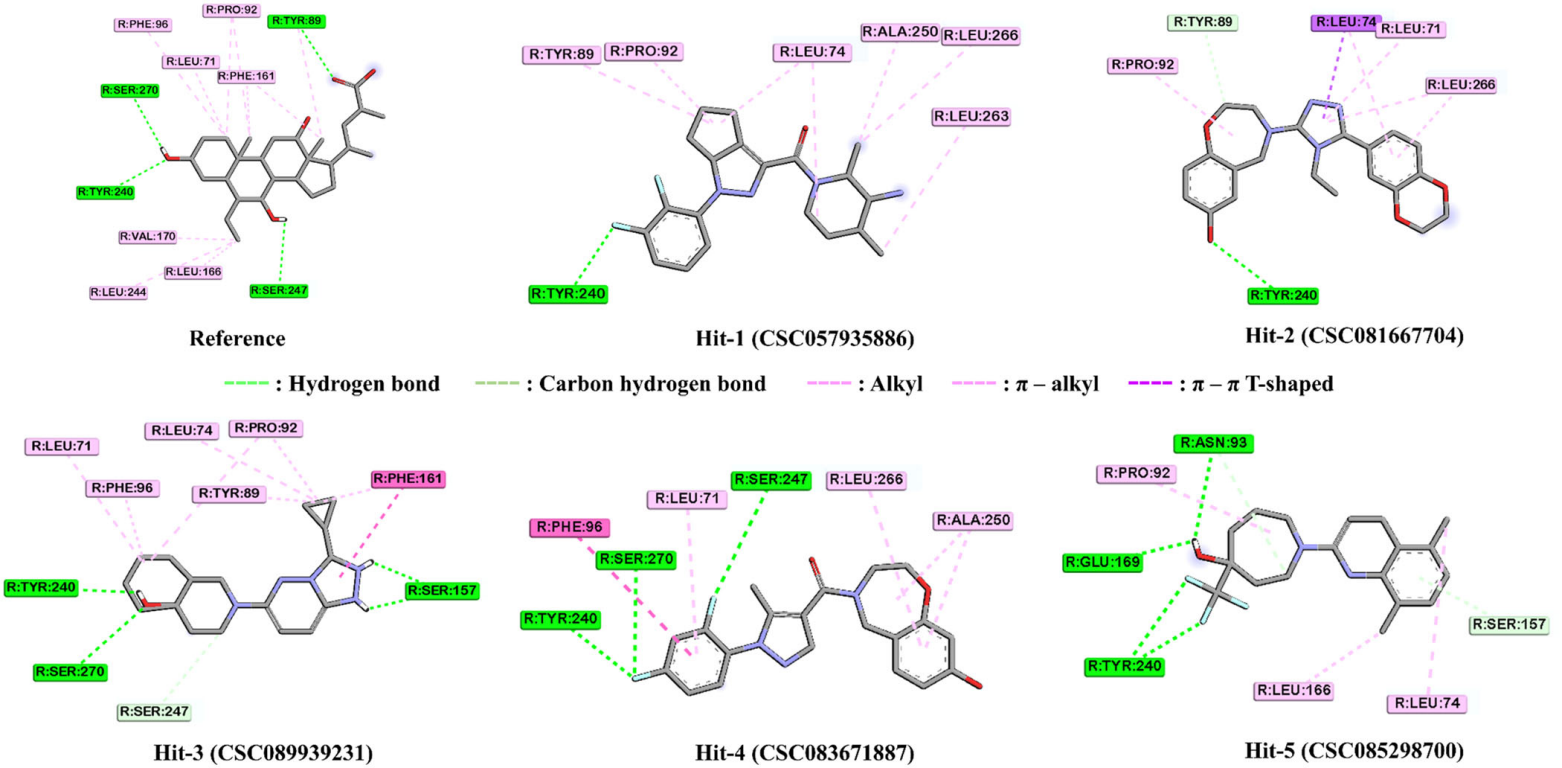
Two‐dimensional (2D) interaction diagrams of five hit compounds in the ligand‐binding domain of Takeda G protein‐coupled receptor 5 (TGR5). (Dashed green line shows a hydrogen bond interaction; dashed purple line indicates a hydrophobic interaction).

Similar to INT‐777, the reference ligand, the five hit compounds formed an H‐bond pattern with the Tyr240 residue, supported by hydrophobic interactions with several other residues in the TGR5 binding cavity. The H‐bond with Tyr240 is critically associated with the activation of TGR5.^[^ ^13^ ^]^ Hit‐1 formed hydrophobic contacts with Leu263 and Leu266, residues that act as “fingerprint readers.”^[^ ^13^ ^]^ Both Hit‐2 and Hit‐4 similarly had this hydrophobic contact with Leu266. Interestingly for Hit‐3, we did not observe hydrophobic contacts with the three leucine residues that serve as a fingerprint reader; however, it formed a carbon‐hydrogen bond with Ser247, another residue in the fingerprint reader.^[^ ^13^ ^]^ Furthermore, there was an H‐bond interaction with Ser157, which was involved in stabilizing the ligand interactions in the binding pocket.^[^ ^8^ ^]^ Remarkably, Hit‐5 had no interactions with the fingerprint reader residues, either hydrophobic or H‐bonds. Hit‐5, on the other hand, formed an H‐bond with Tyr240. All five hit compounds also established a crucial π–π interaction with either Pro92 or Phe96 residues, identical to the reference ligand. The overall interaction analysis results of the five hit compounds are summarized in Table 3 and Figures 5 and 6.

**Table 3:** Intermolecular interactions of INT‐777 (reference) and hit compounds with TGR5 were analyzed with Discovery Studio Visualizer.

Compounds ID	Conventional hydrogen bonds	Carbon hydrogen bond	π‐donor hydrogen	Alkyl bond	π‐Alkyl	π‐sigma	π–π T‐shaped
INT‐777 (Reference)	TYR89 (3.07 Ǻ), TYR240 (2.93 Ǻ), SER247 (2.84 Ǻ), SER270 (2.96 Ǻ)			PRO92 (4.95 Ǻ), LEU71 (4.92 Ǻ), PRO92 (4.83 Ǻ), LEU166 (4.54 Ǻ), VAL170 (4.47 Ǻ), LEU244 (5.10 Ǻ)	TYR89 (5.03 Ǻ), PHE96 (5.23 Ǻ), PHE161 (5.20 Ǻ), PHE161 (4.94 Ǻ)		
CSC057935886 (Hit‐1)	TYR240 (3.00 Ǻ)			LEU74 (5.16 Ǻ), LEU74 (4.51 Ǻ), LEU263 (4.85 Ǻ), ALA250 (3.76 Ǻ), LEU266 (5.27 Ǻ), PRO92 (5.12 Ǻ)	TYR89 (5.34 Ǻ)		
CSC081667704 (Hit‐2)	TYR240 (3.03 Ǻ)	TYR89 (3.40 Ǻ)		PRO92 (5.21 Ǻ)	LEU71 (5.03 Ǻ), LEU266 (5.14 Ǻ), LEU74 (5.41 Ǻ), LEU266 (5.09 Ǻ)	LEU74 (3.90 Ǻ)	
CSC089939231 (Hit‐3)	SER157 (2.31 Ǻ), SER157 (2.70 Ǻ), TYR240 (2.90 Ǻ), SER270 (3.25 Ǻ)	SER247 (3.64 Ǻ)		LEU71 (5.41 Ǻ), PRO92 (5.50 Ǻ), LEU74 (5.10 Ǻ), PRO92 (4.27 Ǻ)	TYR89 (5.08 Ǻ), PHE96 (4.47 Ǻ), PHE161 (5.40 Ǻ)		PHE161 (4.98 Ǻ)
CSC083671887 (Hit‐4)	TYR240 (3.21 Ǻ), SER247 (3.30 Ǻ), SER270 (3.36 Ǻ)			ALA250 (5.00 Ǻ)	ALA250 (5.07 Ǻ), LEU266 (5.27 Ǻ), LEU71 (5.33 Ǻ)		PHE96 (5.34 Ǻ)
CSC085298700 (Hit‐5)	ASN93 (2.42 Ǻ), GLU169 (2.34 Ǻ), TRY240 (3.02 Ǻ), TYR240 3.15 Ǻ)	ASN93 (3.57 Ǻ)	SER157 (3.47 Ǻ)	PRO92 (5.01 Ǻ), LEU74 (4.79 Ǻ), LEU166 (4.51 Ǻ)			

### 2.4 Biological assay of the selected hits

**Figure 7:**
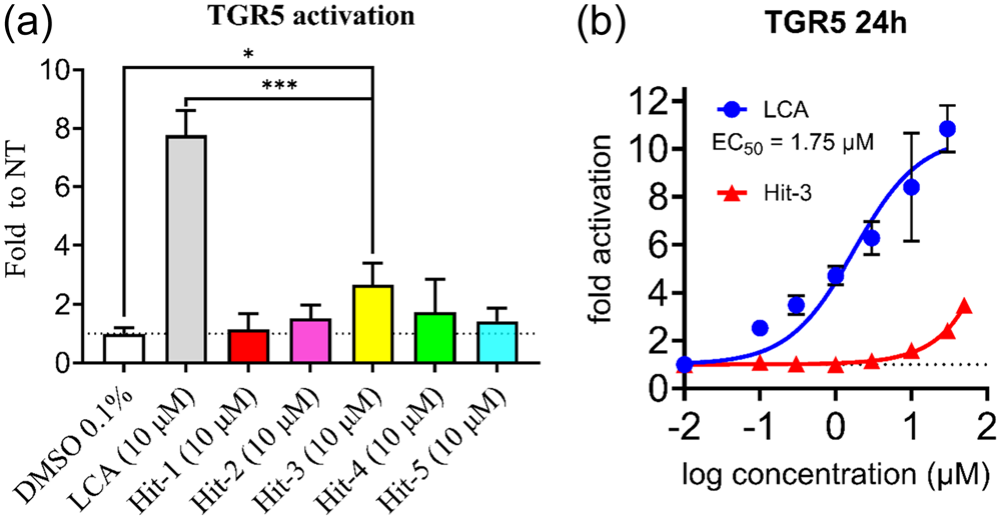
Takeda G protein‐coupled receptor 5 (TGR5) activation in transfected HepG2 cells. (a) All hit compounds at 10 µM. (b) Concentration‐response curves for the activation by lithocholic acid (LCA) and Hit‐3. EC_50_ values were calculated using nonlinear fitting of concentration–response curves. Values are presented as means ± SD from three independent experiments performed in triplicates. *p < 0.05 Hit‐3 versus NT; ***p < 0.001 LCA versus Hit‐3.

During the activation phase, bile acids bind to the ligand‐binding domain (LBD) of TGR5 and initiate downstream signaling via cAMP production, followed by activation of downstream kinases and cAMP response elements (CRE) in the nucleus.^[^ ^26^ ^]^ The potency of the five hit compounds (10 µM concentration) to activate TGR5 was evaluated. For the biological assay, LCA, a secondary bile acid generated through bacterial biotransformation in the intestine,^[^ ^27^ ^]^ was used as a reference compound (Figure 7a). Of the five hit compounds, Hit‐3, a nonbile acid compound, showed a statistically significant (*p *< 0.05) TGR5 activation at 10 µM concentration compared with untreated control (NT). Compared with LCA, a bile acid derivative, the activation by Hit‐3 was significantly lower (*p *< 0.001). In the concentration‐response study (Figure 7b), LCA exerted EC_50_ of 1.75 µM, which is consistent with previous reports (EC_50_ = 1.54 µM (14)). Hit‐3 activated TGR5 in a clear concentration‐dependent manner, but due to the solubility limitations, we were not able to determine the exact EC_50_ value of Hit‐3. The EC_50_ of Hit‐3 is expected to be higher than the EC_50_ of the steroidal reference LCA. Due to the limited availability of the sample from the vendor, Hit‐3 was tested as a mixture of optical isomers. In contrast to the stereoisomer identified in the virtual screening (4aR, 8aR), the other possible isomers of Hit‐3 do not form the critical H‐bond interaction with Tyr240 as predicted by molecular docking (Supporting Information S2: Figure S3) and therefore are probably inactive. The pure isomer of Hit‐3 is therefore expected to have better activity than expressed in Figure 7.

### 2.5 MD simulation of hit molecules

MD studies were performed in an attempt to rationalize the differences in biological activity observed for individual hit compounds. MD simulations were performed for all virtual hits in three independent replicas, followed by an analysis of stability and conformational changes in ligand–receptor interactions.^[^ ^28^, ^29^, ^30^ ^]^ The stability was examined by ligand RMSD analysis, residue fluctuations (RMSF) in the TGR5 binding cavity, and analysis of ligand–receptor H‐bonds. LCA was used as a reference in the MD simulation to ensure consistency with the biological assays.

For all ligands, the RMSD values averaged over the whole trajectory were calculated for each replica (see Table 4) and were used as a simplified measure of the stability of the docked pose. Each ligand showed consistency of trajectories (Supporting Information S2: Figures S4–S9); interestingly, Hit‐3 had the smallest RMSD value (3.20 ± 0.34 Å) compared with the other hit compounds as well as the reference.

**Table 4:** RMSD of heavy atoms of ligands (Å) averaged over the 100 ns trajectory.

	Ref (LCA)	Hit‐1	Hit‐2	Hit‐3	Hit‐4	Hit‐5
Replica‐1	4.19 ± 0.89	3.22 ± 0.38	3.70 ± 0.41	3.21 ± 0.68	6.86 ± 0.95	4.27 ± 0.52
Replica‐2	3.13 ± 0.76	4.55 ± 0.45	2.98 ± 0.44	2.84 ± 0.35	5.75 ± 0.95	3.88 ± 0.76
Replica‐3	3.30 ± 0.51	4.26 ± 0.65	3.21 ± 0.45	3.54 ± 0.41	4.82 ± 0.66	4.72 ± 0.56
Average of replicas	3.54 ± 0.54	4.01 ± 0.34	3.30 ± 0.29	3.20 ± 0.34	5.81 ± 0.63	4.29 ± 0.35

Abbreviations: LCA, lithocholic acid; RMSD, root mean square deviation.

Further analysis was carried out through root‐mean‐square fluctuation (RMSF) to determine the fluctuation of protein residues, particularly the residues in the TGR5 binding cavity. A significant and expected residue fluctuation was detected at the N and C termini and in the loop parts of the protein. On the contrary, the fluctuations were low in parts with ordered secondary secondary structure (Supporting Information S2: Figure S10). As expected, there were no significant fluctuations in the residues forming the TGR5 binding cavity, allowing the interactions between the ligand and the residues in the TGR5 binding cavity to remain stable in time.

**Figure 8:**
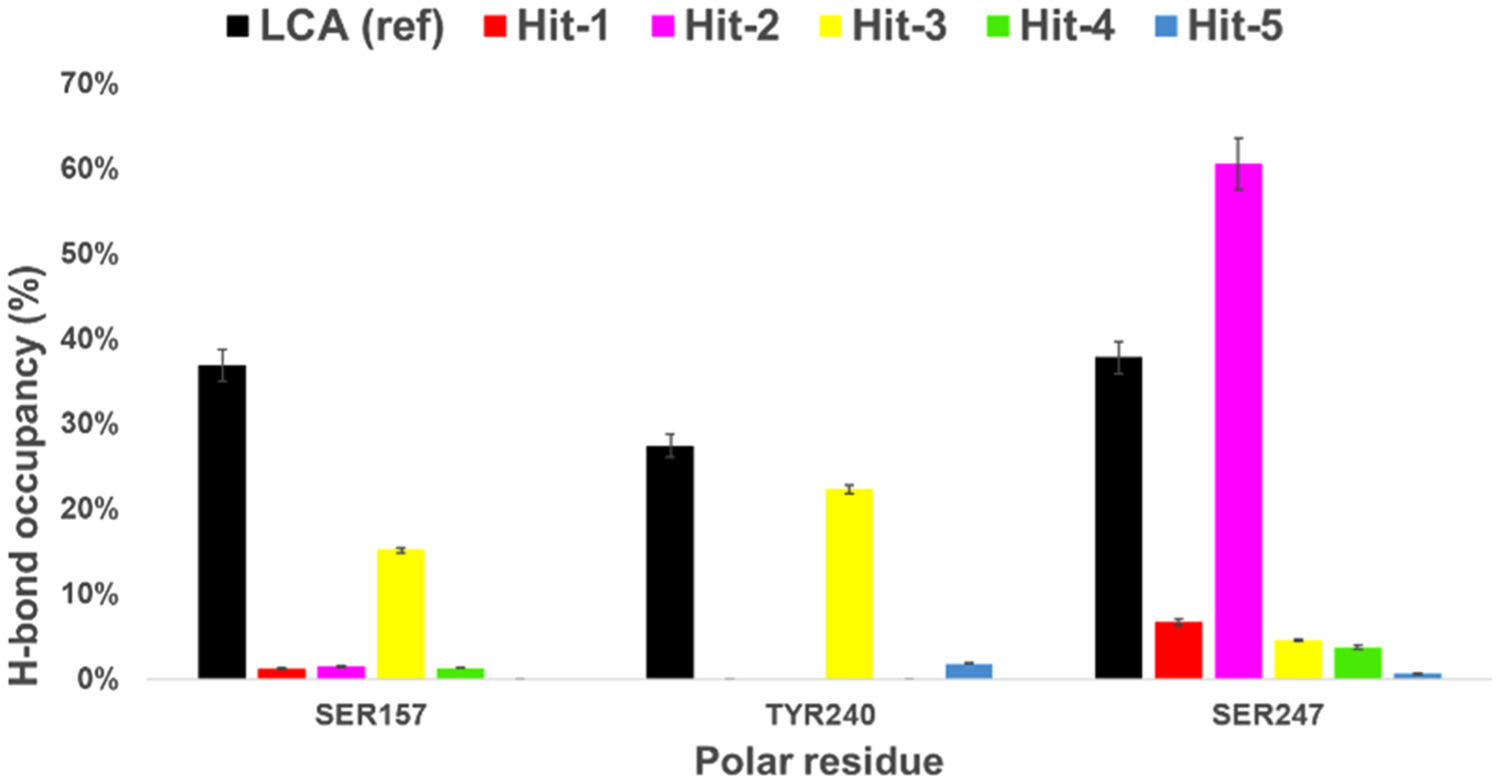
Hydrogen bond occupancies (%) to Takeda G protein‐coupled receptor 5 (TGR5) amino acid residues important for the lithocholic acid (LCA) binding (occupancy > 10%). Occupancies expressed as mean ± SD over three independent replicas, analysis performed at 20–70 ns.

H‐bonds formed in the ligand–receptor complex influence the binding affinity and stability of the ligand–receptor interaction. Figure 8 shows the H‐bond occupancy (%) with amino acid residues that were important for the binding of LCA reference (having H‐bond occupancy >10% in our MD simulations), namely Ser157, Tyr240, and Ser 247. As previously described in the literature, H‐bond with Tyr240 is critically associated with the activation of TGR5,^[^ ^13^ ^]^ and mutations in Tyr240 can decrease or abolish TGR5 activation.^[^ ^4^, ^13^, ^14^ ^]^ Interaction with Ser157 further stabilizes TGR5 ligands through an additional H‐bond.^[^ ^31^ ^]^ Hit‐3 showed a binding pattern similar to LCA, with a significant H‐bond occupancy to two out of three key residues (Tyr240 and Ser157)—the occupancy to the third key residue, Ser247, was around 5%. This key finding distinguished Hit‐3 from the other hits. For example, although Hit‐2 had a high H‐bond occupancy to Ser247 (>50%), it missed the binding to critical Tyr240, rationalizing the inactivity of Hit‐2 in the TGR5 activation assay. Based on our MD simulations and consistent with the measured biological activity, Hit‐3 should be considered the most promising non‐bile acid modulator of TGR5 compared with other hit compounds.

For detailed results of the H‐bond analysis, see Supporting Information (Supporting Information S2: Table S5 and Figure S11).

### 2.6 In silico drug‐likeness analysis and ADMET prediction

The hit compounds were thoroughly analyzed using SwissADME and pkCSM predictors for the physicochemical and pharmacokinetic properties as well as toxicity, and the results are summarized in Supporting Information S2: Tables S6 and S7. Lipinski's Rule of Five was applied to the pharmacophore screening step; therefore, the selected hit compounds satisfied these criteria. All hit compounds also had favorable predicted oral bioavailability, with logP values ranging from 1.52 to 3.57 and predicted intestinal absorption values >50%. Penetration through the blood–brain barrier (BBB) was assessed to minimize brain toxicity; Hit‐5 had the highest predicted BBB permeability of 0.26 compared with the other four hit compounds. Concerning the predicted hepatotoxicity, four of the five hit compounds showed the potential to induce hepatotoxicity except Hit‐2. Dose‐wise, four hit compounds are predicted to fall within the reference value range of the maximum tolerated dose (0.477); however, Hit‐3 was higher than the reference value at 1.25.

## 3 CONCLUSION

With the growing research on the Takeda G protein‐coupled receptor as a potential target for diabetes therapy, identifying new and potent TGR5 modulators, especially nonbile acid derivatives, is crucial. Computational approaches are effective strategies for discovering new lead compounds. This study aimed to discover new TGR5 agonists using bile acid derivatives through pharmacophore‐based screening, molecular docking, biological assays, and MD simulations that rationalize the biological activity of hit compounds. Pharmacophore and molecular docking screenings against the ChemSpace database yielded five hit compounds (Supporting Information S2: Figure S12 and Table S4): CSC057935886 (Hit‐1), CSC081667704 (Hit‐2), CSC089939231 (Hit‐3), CSC083671887 (Hit‐4), and CSC085298700 (Hit‐5), all predicted as potential TGR5 modulators (the InChI codes of the identified hit compounds are provided as Supporting Information). Biological assays confirmed that Hit‐3 activated TGR5 in a concentration‐dependent manner. This finding was supported by MD simulations, which showed that Hit‐3 maintained the critical H‐bond to Tyr240, known to be crucial for TGR5 activations. Although Hit‐3 shows lower potency compared with an LCA reference agonist, this is expected due to the inherently lower activity and suboptimal physicochemical properties typical of hit compounds. Consequently, we propose Hit‐3 as a promising starting point for future hit‐to‐lead optimizations to design potent nonsteroidal TGR5 agonists.

## 4 EXPERIMENTAL

### 4.1 Pharmacophore modeling and validation

The structure of the TGR5 complex with semi‐synthetic bile acid INT‐777 (PDB: 7CFN) as the reference ligand was analyzed for molecular interactions and mapped pharmacophore features.^[^ ^13^ ^]^ The Pharmit server^[^ ^23^ ^]^ was used to construct pharmacophore models with six features. To validate the generated pharmacophore models, the Pharmit server was used to distinguish active compounds from decoys through screening a set of 15 bile acid derivates obtained from PubChem and a set of 437 decoy compounds obtained from the DUD‐E (http://dude.docking.org).^[^ ^28^, ^29^ ^]^ Decoy compounds were generated and calculated based on physical property similarities to bile acid derivatives.

The quality of the pharmacophore model was assessed through a combination of ROC analysis and statistical descriptor calculation. The ROC values were used to determine the sensitivity and specificity of the model, with a range of 0.5–1.0 indicating a highly selective pharmacophore. Scores of 0.9–1.0 indicate an excellent model, whereas higher enrichment factor (E) values indicate its ability to accurately recognize active compounds.^[^ ^20^, ^21^, ^22^, ^23^, ^24^ ^]^ The Güner–Henry (GH) scoring method was used to calculate statistical descriptors, with a score of 0.7–0.8 indicating an excellent model.^[^ ^22^ ^]^ Overall, these assessments provide a comprehensive overview of the efficacy of the pharmacophore model in its ability to distinguish active compounds from decoys.^[^ ^22^, ^32^ ^]^

### 4.2 Pharmacophore‐based virtual screening

The constructed pharmacophore was used to perform virtual screening against a large (250 million conformers of 50 million molecules) and diverse database: ChemSpace. Filters such as ligand/receptor shape filter, Lipinski's rule of five (relative molecular weight ≤500, H‐bond acceptor ≤10, H‐bond donor ≤5, partition coefficient (log P) ≤5.0), and polar surface area (PSA) <140 Å^2^ were applied to select more stringently the ligands of interest. Furthermore, PAINS^[^ ^33^ ^]^ were removed. Finally, a fit value based on mRMSD score was used to discard those hits with significantly deviated poses compared to the pharmacophore model.

### 4.3 Docking‐based virtual screening

Ligands (1625 candidate compounds) obtained from the pharmacophore‐based virtual screening were downloaded from the Pharmit server in SDF format and pretreated by adding polar hydrogens and partial Gasteiger charges using Open Babel,^[^ ^34^ ^]^ followed by energy minimization and conversion to PDBQT using PyRx.^[^ ^35^ ^]^ Protein preparation was carried out in Swiss‐PDBViewer 4.1.0 (10) and AutoDock Tools 1.5.6,^[^ ^25^ ^]^ involving the removal of all water molecules, unnecessary chains, and bound ligands, fixing missing atoms, the addition of polar hydrogen atoms, and the addition of Kollman charges. Finally, the prepared protein was converted to the PDBQT file format. To assess the accuracy of the docking protocol, the INT‐777 ligand was redocked to the TGR5 structure, and the RMSD score was calculated against the original binding pose. Autodock Vina 1.1.2 was used for the virtual screening, with a grid configuration of 25 × 25 × 25 Å and a box center of 95.20 × 122.41 × 114.75 for *x*, *y*, and *z*, respectively, covering ligand‐binding domain and all essential residues inside of it. All other parameters remained at their default values. Candidate compounds with lower binding energy than the reference ligand (INT‐777) were redocked using a modified configuration with exhaustiveness increased to 32 from 8 (default setting) followed by molecular interaction analysis using Discovery Studio Visualizer 2017 and Chimera 1.15.^[^ ^36^ ^]^

### 4.4 Biological assay of the selected hits

All the hit compounds were purchased from SIA ChemSpace, Riga, Latvia, with a declared purity of 100% except Hit‐5 with 93% (detailed information of hit compounds in Supporting Information S2: Table S4). TGR5 activation by hit compounds was assessed in vitro using HepG2 cells transfected with Lipofectamine 3000® (ThermoFisher Scientific) with 200 ng of CRE luciferase reporter vector (CRE‐luc, pGL4. 29[luc2P/CRE/Hygro] Hygro) (Promega), along with 150 ng of TGR5 (GPBAR1‐pcDNA3.1) and 50 ng of pRL‐TK *Renilla* luciferase vectors (Promega). The following day, the cells were exposed to the tested compounds at the indicated concentrations for 5 h. The luciferase activity was measured using Dual Luciferase® Reporter Assay System (Promega) and normalized to *Renilla* luciferase activity. The agonist mode experiments were performed with LCA and hit compounds (all compounds at 10 µM), followed by dose–response assays using increasing concentrations (ranging from 0.01 to 50 µM) of LCA and Hit‐3. The results are presented as a fold change compared with untreated samples (NT). Vehicle (0.1% DMSO) was used as the solvent in all samples, including control samples. The dose–response assays were used to calculate the EC_50_ (the theoretical concentration that provides half‐maximal activation). All experiments were performed in biological triplicates (*n* = 3), each with technical triplicates.

Statistical analyses were performed using GraphPad Prism 9.1.0. software (GraphPad Software, Inc.), with a *p*‐value of <0.05 considered statistically significant. All data are presented as the mean ± standard deviations (SDs) based on at least three independent experiments (*n* = 3). A one‐way analysis of variance (ANOVA) with a Dunnett's or Bonferroni's post‐hoc test was applied to the data if more than two groups were being analyzed. The half‐maximal response (EC_50_) values were calculated using nonlinear fitting of concentration–response curves (log(agonist) vs. response (three parameters)).

### 4.5 MD simulations

To rationalize the activity of the selected hit compounds, MD simulations were performed on each complex with the best hit pose obtained from the docking results. All MD simulations were executed using GROMACS version 2020 with a 100 ns simulation period.^[^ ^37^ ^]^ Protein and ligand topology files were prepared in advance, with the protein topology files obtained by applying the CHARMM36 all‐atom force field^[^ ^38^ ^]^ and the ligand topology files generated by adding hydrogen atoms using Avogadro^[^ ^39^ ^]^ and submitting them to the CgenFF web server.^[^ ^40^ ^]^ All complexes were solvated in a dodecahedron box with TIP3P water type and chloride ions to neutralize the system and with a minimum distance of 1 Å maintained between the box edge and the protein. Energy minimization was then applied to the system using Verlet's cutoff scheme, resulting in a maximum force of less than 10 kJ/mol/nm with a maximum of 50,000 steps and eliminating unwanted steric collisions and bad contacts. The equilibration was carried out under canonical ensemble (NVT) and isothermal‐isobaric ensemble (NPT). The NVT equilibration step was performed with a constant volume at 300 K using the Berendsen thermostat algorithm for 100 ps. Then, the NPT equilibration was executed at a constant pressure of 1 bar at 300 K using the Parrinello–Rahman barostat for 100 ps. Finally, the 100 ns production runs (NPT) were performed in three replicas. MD trajectories were analyzed using various scripts built‐in to the GROMACS software package. “gmx rms” and “gmx rmsf” were used to determine the RMSD and RMSF. Hydrogen bond parameters during the simulation were analyzed using “gmx hbond.” All graphs were plotted using Excel version 2108.

### 4.6 Drug‐likeness and ADMET properties

The physicochemical properties, pharmacokinetic parameters, and toxicity of the virtual screening hit compounds were estimated utilizing SwissADME and pkCSM web platforms.^[^ ^41^, ^42^ ^]^ SwissADME was used to analyze relevant physicochemical properties such as Lipinski parameters, lipophilicity, water solubility, and drug‐likeness, while pkCSM was employed to estimate the pharmacokinetic parameters (ADME) and toxicity of the hit compounds.

## CONFLICTS OF INTEREST STATEMENT

The authors declare no conflicts of interest.

## Supporting information

Supporting information.

Supporting information.

## Data Availability

All data obtained in this study are public and can be accessed by anyone. Data have been included in the manuscript and supplementary files. Structures of small MW compounds were downloaded in SDF format from the screening results on the ChemSpace database listed on the Pharmit webserver (https://pharmit.csb.pitt.edu/). The structure of the receptor is available in the RSCB PDB database (www.rcsb.org), PDB ID: 7CFN. The input and output data files of the molecular docking and the trajectory files of the MD simulation are attached to the Zenodo cloud storage (https://doi.org/10.5281/zenodo.10560686). Physicochemical, pharmacokinetic, and toxicity properties as well as PAINS were calculated by SwissADME and pkCSM.^[^ ^41^, ^42^ ^]^ The third‐party software used in this study: PyRx 0.8 with Open Babel tool (https://pyrx.sourceforge.io/home), free license; Swiss‐PDBViewer (https://spdbv.unil.ch/), distributed under license; AutoDock Tools 1.5.6 (https://ccsb.scripps.edu/mgltools/1-5-6/), distributed under license; Autodock Vina 1.1.2 (https://vina.scripps.edu/), an open‐source software; Chimera 1.15 (https://www.cgl.ucsf.edu/chimera/), free license; Discovery Studio Visualizer 2017 (https://discover.3ds.com/discovery-studio-visualizer-download), free license; Avogadro (https://avogadro.cc/), an open‐source software; CgenFF webserver (https://cgenff.silcsbio.com/); Gromacs v.2020 (https://www.gromacs.org/), an open‐source software; Excel v.2108 (https://www.microsoft.com/en-us/microsoft-365/excel), distributed under license.
