# Supplementary material for: The discovery of a new nonbile acid modulator of Takeda G protein‐coupled receptor 5: An integrated computational approach
Source: Arch Pharm (Weinheim). 2025 Jan 13;358(1):e2400423. doi: 10.1002/ardp.202400423 (PMC11726147; doi:10.1002/ardp.202400423)
Supplement: Supplementary file 1 — Supporting information. [file ARDP-358-e2400423-s002.doc]

**Supplemental Material: Novel Compounds and Biological Screening Results**

The discovery of a new non-bile acid modulator of Takeda G protein-coupled Receptor 5: An Integrated Computational Approach

Rudy Salam^1,4^, Michael Bakker^1^, Mária Krutáková^2^, Alžbeta Štefela^2^, Petr Pávek^2^, Jurjen Duintjer Tebbens^1^, Jan Zitko^3,*^

1 Department of Biophysics and Physical Chemistry, Faculty of Pharmacy, Charles University, Hradec Králové, Czech Republic

2 Department of Pharmacology and Toxicology, Faculty of Pharmacy, Charles University, Hradec Králové, Czech Republic

3 Department of Pharmaceutical Chemistry and Pharmaceutical Analysis, Faculty of Pharmacy, Charles University, Hradec Králové, Czech Republic

4 Department of Pharmacy, Faculty of Medicine, Universitas Brawijaya, Malang, Indonesia

Corresponding author – full address
Assoc. Prof. PharmD. Jan Zitko, PhD., Department of Pharmaceutical Chemistry and Pharmaceutical Analysis, Faculty of Pharmacy, Charles University, Heyrovského 1203, Hradec Králové, Czech Republic, jan.zitko@faf.cuni.cz

| **HIT No.** | **Compound ID** | **InChI** | **Biological Activity (yes/no)^a^** |
| --- | --- | --- | --- |
| HIT-1 | CSC057935886 | InChI=1S/C20H24F2N4O/c1-11-9-10-25(12(2)18(11)23)20(27)19-13-5-3-7-15(13)26(24-19)16-8-4-6-14(21)17(16)22/h4,6,8,11-12,18H,3,5,7,9-10,23H2,1-2H3 | No |
| HIT-2 | CSC081667704 | InChI=1S/C21H22N4O4/c1-2-25-20(14-3-5-18-19(12-14)29-10-9-28-18)22-23-21(25)24-7-8-27-17-6-4-16(26)11-15(17)13-24/h3-6,11-12,26H,2,7-10,13H2,1H3 | No |
| HIT-3 | CSC089939231 | InChI=1S/C17H23N5O/c23-17-8-2-1-3-13(17)11-21(10-9-17)15-7-6-14-18-19-16(12-4-5-12)22(14)20-15/h6-7,12-13,23H,1-5,8-11H2/t13-,17+/m0/s1 | Yes |
| HIT-4 | CSC083671887 | InChI=1S/C20H17F2N3O3/c1-12-16(10-23-25(12)18-5-3-14(21)8-17(18)22)20(27)24-6-7-28-19-9-15(26)4-2-13(19)11-24/h2-5,8-10,26H,6-7,11H2,1H3 | No |
| HIT-5 | CSC085298700 | InChI=1S/C17H18ClF3N2O/c1-11-3-5-13(18)12-4-6-14(22-15(11)12)23-9-2-7-16(24,8-10-23)17(19,20)21/h3-6,24H,2,7-10H2,1H3 | No |

^a^ The hit compounds were obtained from pharmacophore screening followed by molecular docking. The hits were selected as they fulfilled the criteria of pharmacophore features, docking score less than 9.2 kcal/mol, and essential interaction similarity with the reference ligand. Furthermore, biological assays were performed to confirm the TGR5 activation of the selected hit compounds.
