# Supplementary material for: The discovery of a new nonbile acid modulator of Takeda G protein‐coupled receptor 5: An integrated computational approach
Source: Arch Pharm (Weinheim). 2025 Jan 13;358(1):e2400423. doi: 10.1002/ardp.202400423 (PMC11726147; doi:10.1002/ardp.202400423)
Supplement: Supplementary file 2 — Supporting information. [file ARDP-358-e2400423-s001.pdf]

Table S1. Active compounds in the test set – obtained from PubChem

Molecule Name	MW	logP	HBA	HBD	PSA	Smiles
Ursodeoxycholic acid	392.58	4.03	4	3	77.76	C[C@H](CCC(O)=O)[C@@H](CC1)[C@@](C)(CC[C@@H]2[C@@](C)(CC[C@H](C3)O)[C@@H]3C3)[C@@H]1[C@@H]2[C@H]3O
Taurolithocholic acid	483.71	2.94	6	3	112.08	C[C@H](CCC(NCCS(O)(=O)=O)=O)[C@@H](CC1)[C@@](C)(CC2)[C@@H]1[C@H](CC1)[C@H]2[C@@](C)(CC2)[C@H]1C[C@@H]2O
Taurocholic acid	515.71	1.24	8	5	152.54	C[C@H](CCC(NCCS(O)(=O)=O)=O)[C@@H](CC[C@H]1[C@@H]([C@H](C2)[C@@](C)(CC[C@H](C3)O)[C@@H]3C3)[C@@H]3O)[C@@]1(C)[C@H]2O
Obeticholic acid	420.63	4.76	4	3	77.76	CC[C@H]([C@H](C[C@@H](CC1)O)[C@@]1(C)[C@@H](CC1)[C@@H]2[C@H](CC3)[C@@]1(C)[C@H]3[C@H](C)CCC(O)=O)[C@H]2O
Lithocholic acid	376.58	4.88	3	2	57.53	C[C@H](CCC(O)=O)[C@@H](CC1)[C@@](C)(CC2)[C@@H]1[C@H](CC1)[C@H]2[C@@](C)(CC2)[C@H]1C[C@@H]2O
Hyodeoxycholic acid	392.58	4.03	4	3	77.76	C[C@H](CCC(O)=O)[C@@H](CC1)[C@@](C)(CC2)[C@@H]1[C@H](C1)[C@H]2[C@@](C)(CC[C@H](C2)O)[C@@H]2[C@H]1O
Deoxycholic acid	392.58	4.03	4	3	77.76	C[C@H](CCC(O)=O)[C@@H](CC[C@H]1[C@H](CC2)[C@H](C3)[C@@](C)(CC4)[C@H]2C[C@@H]4O)[C@@]1(C)[C@H]3O
Cholic acid	408.58	3.18	5	4	97.99	C[C@H](CCC(O)=O)[C@@H](CC[C@H]1[C@@H]([C@H](C2)[C@@](C)(CC[C@H](C3)O)[C@@H]3C3)[C@@H]3O)[C@@]1(C)[C@H]2O
Chenodeoxycholic acid	392.58	4.03	4	3	77.76	C[C@H](CCC(O)=O)[C@@H](CC1)[C@@](C)(CC[C@@H]2[C@@](C)(CC[C@H](C3)O)[C@@H]3C3)[C@@H]1[C@@H]2[C@@H]3O
INT-777	450.66	4.12	5	4	97.99	CC[C@H]([C@H](C[C@@H](CC1)O)[C@@]1(C)[C@@H](C1)[C@@H]2[C@H](CC[C@@H]3[C@H](C)C[C@H](C)C(O)=O)[C@]3(C)[C@H]1O)[C@H]2O
MI-126*	420.63	4.78	4	3	77.76	CC[C@@]1([C@@H]([C@H](CC2)[C@](C)(CC3)[C@H]2[C@H](C)CCC(O)=O)[C@H]3[C@@](C)(CC[C@H](C2)O)[C@@H]2C1)O
MI-157E*	402.62	5.51	3	2	57.53	C[C@H](CCC(O)=O)[C@@H](CC1)[C@@](C)(CC[C@@H]2[C@@](C)(CC[C@H](C3)O)[C@@H]3C3)[C@@H]1[C@@H]2/C\3=C/C
MI-172*	434.66	5.00	4	3	77.76	CC(C)[C@@]1([C@@H]([C@H](CC2)[C@](C)(CC3)[C@H]2[C@H](C)CCC(O)=O)[C@H]3[C@@](C)(CC[C@H](C2)O)[C@@H]2C1)O
MI-167*	406.60	4.33	4	3	77.76	C[C@H](CCC(O)=O)[C@@H](CC1)[C@@](C)(CC[C@@H]2[C@@](C)(CC[C@H](C3)O)[C@@H]3C3)[C@@H]1[C@@H]2[C@]3(C)O
MI-160*	418.62	4.60	4	3	77.76	C[C@H](CCC(O)=O)[C@@H](CC1)[C@@](C)(CC[C@@H]2[C@@](C)(CC[C@H](C3)O)[C@@H]3C3)[C@@H]1[C@@H]2[C@]3(C=C)O

* Synthesized compound from our laboratory without PubChem CID; molecular weight (MW); partition coefficient (logP); hydrogen bond acceptor (HBA); hydrogen bond donor (HBD); polar surface area (PSA)

Table S2. Decoy compounds obtained from the Database of Useful Decoys (DUD-E) in the test set

Molecule Identifier	MW	logP	HBA	HBD	PSA	Smiles
CHEMBL98821	444.91	3.91	7	3	104.73	OC([C@@H](Cc(cc1)ccc1OC(NC1CCCCC1)=O)NC(c(cccc1)c1Cl)=O)=O
CHEMBL98564	364.48	4.65	3	2	57.53	CC(C)(CCC1(C)C)c2c1cc([C@H](c1ccc(/C=C/C(O)=O)cc1)O)cc2
CHEMBL97689	364.48	4.65	3	2	57.53	CC(C)(CCC1(C)C)c2c1cc([C@H](c1cccc(/C=C\C(O)=O)c1)O)cc2
CHEMBL97080	364.48	4.67	3	2	57.53	CC(C)(CCC1(C)C)c2c1cc([C@H](/C=C\c(cc1)ccc1C(O)=O)O)cc2
CHEMBL94775	372.38	2.53	6	4	131.71	NC(c(cc1)cc2c1[nH]c(-c1cc([N+]([O-])=O)cc(-c3ccccc3)c1O)c2)=N
CHEMBL94188	497.52	3.33	7	3	97.26	N#Cc1c(C2CCN(CCCNC(C([C@@H](c(cc3)cc(F)c3F)N3)=CNC3=O)=O)CC2)ccc(F)c1
CHEMBL93947	448.57	3.50	7	3	105.46	CC(C)(C)C(NCc(cc1)ccc1NC(N(C[C@@H](c1cc(C#N)ccc1)O)C1CCC1)=O)=O
CHEMBL92598	450.54	1.84	7	4	124.98	NC(c(cc1)ccc1C(N(CC1)Cc2c1c(cccc1)c1[n]2Cc1cc(C(N)=N)ccc1)=O)=N
CHEMBL92328	511.55	4.00	7	3	97.26	CC(NC(N[C@H]1c(cc2)cc(F)c2F)=O)=C1C(NCCCN(CC1)CCC1c(ccc(F)c1)c1C#N)=O
CHEMBL92007	367.40	5.15	4	2	66.40	OC(c(cccc1)c1-c(cccc1)c1C(Nc1cc2ccccc2cc1)=O)=O
CHEMBL89297	400.48	3.15	6	3	87.46	Nc1cccc(CN(c2c(CC[C@@H]3NC(Nc4ccccc4)=O)cccc2)C3=O)c1
CHEMBL89283	460.16	5.27	4	4	80.92	C[C@H](Cc(cc(c(O)c1)O)c1Br)[C@@H](C)Cc(cc(c(O)c1)O)c1Br
CHEMBL88904	340.38	4.03	4	2	65.98	OC(c(cc1)ccc1-c1cc(-c2ccncc2)c(-c2ccccc2)[nH]1)=O

CHEMBL86489	482.54	3.92	8	3	105.74	CCCN(c1c2cccc1)c(ccc(NC(Cc1c[nH]c3c1cccc3)=O)c1)c1N(CC(O)=O)C2=O
CHEMBL84470	348.48	5.74	2	2	40.46	CCC[C@H](C1)C(c(cc2)c(C[C@H]3CCC)cc2O)=C3c(cc2)c1cc2O
CHEMBL83793	344.87	4.45	3	2	84.18	CC(C)c(cc1)ccc1Sc1c(C(N)=O)[nH]c(cc2)c1cc2Cl
CHEMBL82943	429.49	5.68	4	2	66.40	CC1(C)c(ccc(C(Nc(cc2)cc(F)c2C(O)=O)=O)c2)c2C(c2ccc(C)cc2)=CC1
CHEMBL78153	394.91	4.63	6	2	164.20	C/C(\c(cccc1)[n+]1[O-])=N\N=C(/N/N=C(/Nc(cccc1)c1Cl)\S)\S
CHEMBL77833	383.23	3.81	6	3	87.66	CC(C)(C(O)=O)Oc(cc1)ccc1NC(Nc1cccc(Cl)c1Cl)=O
CHEMBL77731	375.05	4.61	3	2	49.33	OC(Cc(c(Nc(c(Cl)ccc1)c1Cl)ccc1)c1Br)=O
CHEMBL77193	383.23	3.81	6	3	87.66	CC(C)(C(O)=O)Oc(cc1)ccc1NC(Nc(cc1)cc(Cl)c1Cl)=O
CHEMBL76939	337.46	4.27	3	2	49.33	CC(C)(CCC1(C)C)c2c1cc(CNc1cccc(C(O)=O)c1)cc2
CHEMBL76841	417.68	4.42	6	3	87.66	CC(C)(C(O)=O)Oc(cc1)ccc1NC(Nc(cc1Cl)cc(Cl)c1Cl)=O
CHEMBL76636	383.23	3.81	6	3	87.66	CC(C)(C(O)=O)Oc(cc1)ccc1NC(Nc(cc(cc1)Cl)c1Cl)=O
CHEMBL76388	423.36	5.23	6	2	151.63	C/C(\c1ncccc1)=N/N=C(\N/N=C(/Nc1cccc(Br)c1)\S)/S
CHEMBL76242	423.36	5.23	6	2	151.63	C/C(\c1ncccc1)=N/N=C(\N/N=C(/Nc(cccc1)c1Br)\S)/S
CHEMBL76198	383.23	3.81	6	3	87.66	CC(C)(C(O)=O)Oc(cc1)ccc1NC(Nc(ccc(Cl)c1)c1Cl)=O
CHEMBL76029	383.23	3.81	6	3	87.66	CC(C)(C(O)=O)Oc(cc1)ccc1NC(Nc1cc(Cl)cc(Cl)c1)=O
CHEMBL7598	429.13	5.48	4	2	57.78	CCNC(c1nc(-c2cc(Cl)cc(Cl)c2)c(-c2cc(Cl)cc(Cl)c2)[nH]1)=O
CHEMBL75333	521.41	3.59	7	3	108.51	NC(c1cccc(O[C@@H](C(Nc(ccc(C(N2CCCC2)=O)c2)c2Br)=O)c2ccccc2)c1)=N
CHEMBL75263	378.91	5.11	6	2	151.63	C/C(\c1ncccc1)=N/N=C(\N/N=C(\Nc1cccc(Cl)c1)/S)/S
CHEMBL74928	378.91	5.11	6	2	151.63	C/C(\c1ncccc1)=N\N=C(\N/N=C(/Nc(cc1)ccc1Cl)\S)/S
CHEMBL74446	413.36	5.71	6	2	151.63	C/C(\c1ncccc1)=N/N=C(\N/N=C(\Nc(cc(cc1)Cl)c1Cl)/S)/S
CHEMBL74413	413.36	5.71	6	2	151.63	C/C(\c1ncccc1)=N/N=C(/N/N=C(\Nc1cccc(Cl)c1Cl)/S)\S
CHEMBL74393	378.91	5.57	6	2	151.63	Cc1cccc(/C=N/N=C(\N/N=C(/Nc(cccc2)c2Cl)\S)/S)n1
CHEMBL74303	400.57	6.08	6	2	151.63	CC(C)(C)c(cc1)ccc1N/C(\S)=N/N/C(\S)=N\N=C(\C)/c1ncccc1
CHEMBL74250	392.94	5.45	6	2	151.63	C/C(\c1ncccc1)=N\N=C(\N/N=C(\Nc(cc1)c(C)cc1Cl)/S)/S
CHEMBL74153	402.59	4.87	6	2	151.63	C/C(\c1ncccc1)=N\N=C(/N/N=C(\NC1(C[C@H](C2)C3)C[C@H]3C[C@H]2C1)/S)\S
CHEMBL73766	337.46	4.27	3	2	49.33	CC(C)(CCC1(C)C)c2c1cc(CNc(cccc1)c1C(O)=O)cc2
CHEMBL72550	398.44	3.24	5	3	69.28	O=C1Nc(cccc2)c2C(c(cccc2)c2F)=N[C@H]1NCc1cc(cccc2)c2[nH]1
CHEMBL71344	391.33	4.57	4	1	99.34	CCN/C(\Cc(c(Cl)ccc1)c1Cl)=N\C(/S)=N\c(cc1)ccc1C#N
CHEMBL71011	377.30	4.09	4	3	103.79	N=C(CCc(c(Cl)ccc1)c1Cl)NC(Nc(cc1)ccc1C#N)=S
CHEMBL70649	439.37	5.87	4	2	92.30	N#Cc(cc1)ccc1NC(/N=C(/Cc(c(Cl)ccc1)c1Cl)\Nc1ccccc1)=S
CHEMBL70405	394.48	3.51	5	3	69.28	O=C1Nc(cccc2)c2C(c2ccccc2)=N[C@H]1NCCc1c[nH]c2c1cccc2
CHEMBL64662	416.69	6.73	2	2	40.46	C[C@H]([C@@H](CCC(C)(C)C)O)[C@H](CC1)[C@](C)(CC2)[C@@H]1[C@H]1[C@H]2[C@@](C)(CC[C@@H](C2)O)C2=CC1
CHEMBL63912	362.47	2.90	4	3	61.36	CCNC(c(cc1)ccc1/C(\c(cc1)ccc1O)=C1\C[C@H](CC2)N[C@H]2C\1)=O

CHEMBL63408	437.88	6.34	4	2	54.02	CCc1ccc2ncc(NC(Nc(ccc(F)c3)c3F)=O)c(-c(cccc3)c3Cl)c2c1
CHEMBL63004	360.54	4.43	5	3	96.99	C[C@@](CC[C@H](C1)C2CCCCC2)([C@@H](CC2)/C=C(\C)/C=N\N=C(N)N)[C@@]12O
CHEMBL62807	382.44	3.10	6	4	108.01	C[C@@H](CCCC1)[C@@H]1Oc1cccc(-c([nH]c2c3)nc2cc(C(N)=N)c3F)c1O
CHEMBL62794	419.28	4.37	4	2	66.56	Cc(cc(cc1)F)c1-c1c(CO)ccc(N(C(N)=O)c(c(Cl)ccc2)c2Cl)c1
CHEMBL62509	382.44	3.10	6	4	108.01	C[C@H](CC1)CC[C@H]1Oc1cccc(-c([nH]c2c3)nc2cc(C(N)=N)c3F)c1O
CHEMBL61942	379.24	3.46	5	3	78.43	OC([C@H]1Nc2cc(Cl)cc(Cl)c2[C@H](CC(Nc2ccccc2)=O)C1)=O
CHEMBL61740	379.24	2.71	5	3	78.43	OC([C@H](C1)Nc2cc(Cl)cc(Cl)c2[C@@H]1C(NCc1ccccc1)=O)=O
CHEMBL61658	393.27	3.01	5	3	78.43	Cc1ccc(CC(N[C@H](C[C@@H](C(O)=O)Nc2cc(Cl)c3)c2c3Cl)=O)cc1
CHEMBL60348	476.66	5.11	5	3	68.72	CCCCc1c(CC(C)C)[nH]c([C@@H]([C@]2(CCN(CC3CC3)[C@@H]3C4)c5c4cc4)Oc5c4O)c1C[C@]23O
CHEMBL59186	384.88	4.38	3	2	39.26	OC1(CCN(Cc2ccc(-c(cc3)ccc3F)[nH]2)CC1)c(cc1)ccc1Cl
CHEMBL58405	385.29	3.02	5	3	78.43	OC([C@H](C1)Nc2cc(Cl)cc(Cl)c2[C@H]1NC(CC1CCCCC1)=O)=O
CHEMBL54373	426.64	5.89	3	3	60.69	CC[C@@H](C)[C@H](C#C[C@H](C)[C@@H](CC1)[C@@](C)(CCC2)[C@H]1/C\2=C/C=C(/C[C@H](C[C@H]1O)O)\C1=C)O
CHEMBL518378	448.69	4.62	4	4	80.92	CC(C)[C@@H](CO)/C=C\[C@@H](C)[C@H](C[C@H]1O)[C@](C)(CC2)[C@H]1[C@@H](C1)[C@H]2[C@](C)(CC[C@H](C2)O)[C@@H]2[C@H]1O
CHEMBL50561	485.58	1.67	7	3	112.73	CC(C)(CC(N[C@H](CCc(cccc1)c1N1Cc(cc2)ccc2-c(cccc2)c2C(O)=O)C1=O)=O)N
CHEMBL49839	358.85	3.51	4	2	100.49	OC(CC([C@@H](c1ccccc1)N1)=C(c(cc2)ccc2Cl)N=C1S)=O
CHEMBL492814	460.70	5.10	4	4	80.92	CC[C@H](C(C)C)/C=C\[C@@H](C)[C@H](CC1)[C@@](C)(CC[C@]2([C@](C)(CC[C@@H](C3)O)[C@]33O)O)[C@H]1C2=C[C@@H]3O
CHEMBL49053	358.85	3.51	4	2	100.49	OC(CC([C@@H](c(cc1)ccc1Cl)N1)=C(c2ccccc2)N=C1S)=O
CHEMBL48923	366.31	4.11	4	2	60.21	CC(C)CC/N=C(/Nc1cc(C(F)(F)F)cc(C(F)(F)F)c1)\NC#N
CHEMBL479490	462.62	3.38	6	4	115.06	C[C@H](CCC(O)=O)[C@H](CC1)[C@](C)(CC2)[C@]1(C)[C@H](CC[C@@H]1[C@@](C)([C@H](C3)O)C(O)=O)[C@]2(C2)[C@@]12[C@@H]3O
CHEMBL47892	487.60	4.25	7	3	108.39	C[C@H](CC1)C(C)(C)[C@@]1(C)C(N[C@H](Cc(cc1)ccc1NC(c1ccnc2ccccc12)=O)C(O)=O)=O
CHEMBL47818	470.62	2.92	6	3	101.45	CC(C)(CC(N[C@H](CCc(cccc1)c1N1Cc(cc2)ccc2-c2c(CN)cccc2)C1=O)=O)N
CHEMBL47734	484.60	3.00	7	3	118.52	CC(C)(CC(N[C@@H](CCc(cccc1)c1N1Cc(cc2)ccc2-c(cccc2)c2C(N)=O)C1=O)=O)N
CHEMBL47672	449.55	6.12	4	3	69.56	Oc1ccc([C@@H](CCc(cc2)c3cc2O)[C@@H]3NC(C(c2ccccc2)c2ccccc2)=O)cc1
CHEMBL47622	359.25	5.40	2	2	40.46	OCc1cc(Cl)c(-c(cc2)cc(Cc3ccccc3)c2O)c(Cl)c1
CHEMBL47573	459.54	3.57	7	3	108.39	CC1(C)C(C)(C)C1C(N[C@@H](Cc(cc1)ccc1NC(c1ccnc2ccccc12)=O)C(O)=O)=O
CHEMBL47538	486.35	3.55	7	3	108.39	C[C@@](C1)(C1(Cl)Cl)C(N[C@@H](Cc(cc1)ccc1NC(c1ccnc2ccccc12)=O)C(O)=O)=O
CHEMBL474074	462.71	5.23	4	4	80.92	C[C@@H](CO)[C@@H](C)/C(\C)=C/[C@@H](C)[C@@H](C[C@@H]1O)[C@](C)(CC2)[C@@H]1[C@H](C1)[C@@H]2[C@](C)(CC[C@H](C2)O)[C@H]2[C@H]1O
CHEMBL473038	488.71	4.68	5	4	97.99	C[C@H](C/C=C/C(C)(C)O)[C@@H](CC1)[C@](C)(CC2)[C@]1(C)[C@H](CC[C@H]1[C@@](C)([C@H](C3)O)C(O)=O)[C@@]2(C2)[C@]12[C@@H]3O
CHEMBL47216	518.37	4.22	7	3	108.39	OC([C@H](Cc(cc1)ccc1NC(c1ccnc2ccccc12)=O)NC(c(cccc1)c1Br)=O)=O
CHEMBL47174	357.45	4.72	3	2	49.33	Oc1cc([C@@H](CCC2)NC(C(c3ccccc3)c3ccccc3)=O)c2cc1
CHEMBL468878	488.71	5.07	5	4	97.99	C[C@H](CCC=C(C)C)[C@H](C[C@H]1O)[C@](C)(CC2)[C@@]1(C)[C@@H](CC[C@@H]1[C@](C)([C@@H](C3)O)C(O)=O)[C@@]2(C2)[C@]12[C@H]3O
CHEMBL46751	475.45	3.69	7	3	108.39	OC([C@H](Cc(cc1)ccc1NC(c1ccnc2ccccc12)=O)NC(c(c(F)ccc1)c1F)=O)=O
CHEMBL46725	372.88	3.86	4	2	100.49	Cc(cc1)ccc1C1=C(CC(O)=O)[C@H](c(cc2)ccc2Cl)NC(S)=N1

CHEMBL467206	488.71	5.07	5	4	97.99	C[C@@H](CCC=C(C)C)[C@@H]([C@H](C1)O)[C@](C)(CC2)[C@]1(C)[C@H](CC[C@@H]1[C@@](C)([C@H](C3)O)C(O)=O)[C@]2(C2)[C@@]12[C@H]3O
CHEMBL465355	448.69	4.62	4	4	80.92	C[C@H](CO)[C@H](C)/C=C/[C@@H](C)[C@@H](C[C@@H]1O)[C@](C)(CC2)[C@@H]1[C@H](C1)[C@H]2[C@@](C)(CC[C@@H](C2)O)[C@H]2[C@@H]1O
CHEMBL463414	446.67	4.69	4	4	80.92	C[C@@H](CO)[C@@H](C)/C=C/[C@H](C)[C@@H](C[C@H]1O)[C@](C)(CC2)C1=C(C1)[C@@H]2[C@@](C)(CC[C@@H](C2)O)[C@H]2[C@@H]1O
CHEMBL46076	456.61	3.01	7	3	119.60	CC(C)C[C@@H](C(N1C(C)(C)SC[C@@H]1C(N/C=C/c1c[nH]c2c1cccc2)=O)=O)NC(C)=O
CHEMBL45937	419.36	5.26	3	2	49.33	OC(C(F)(F)F)(C(F)(F)F)c(ccc1ccccc11)c1NC(CC1CCCC1)=O
CHEMBL4543462	390.48	4.97	2	2	40.46	OC(C#CC(O)(c1ccccc1)c1ccccc1)(c1ccccc1)c1ccccc1
CHEMBL45361	373.41	4.09	5	4	89.79	Oc(cc1)cc(C#Cc(cc2)cc(C(NCCc3ccccc3)=O)c2O)c1O
CHEMBL4521454	366.81	3.61	6	3	83.12	O=C(c1ncccc1)Nc1cccc(NC(Nc(cc2)ccc2Cl)=O)c1
CHEMBL4517008	382.42	4.20	6	3	83.12	O=C(c1ncccc1)Nc1cccc(NC(Nc2cc3ccccc3cc2)=O)c1
CHEMBL4514933	447.66	3.66	5	4	89.79	C[C@@H](CCC(NC1CC1)=O)[C@@H](CC[C@@H]1[C@H]([C@H](C2)[C@](C)(CC[C@@H](C3)O)[C@H]3C3)[C@H]3O)[C@@]1(C)[C@H]2O
CHEMBL44946	467.52	4.18	7	3	108.39	Cc1cccc(C)c1C(N[C@@H](Cc(cc1)ccc1NC(c1ccnc2ccccc12)=O)C(O)=O)=O
CHEMBL4483797	489.74	4.69	5	4	89.79	C[C@H](CCC(NC1CCCCC1)=O)[C@H](CC[C@@H]1[C@@H]([C@H](C2)[C@](C)(CC[C@H](C3)O)[C@@H]3C3)[C@@H]3O)[C@@]1(C)[C@H]2O
CHEMBL4483387	373.84	3.44	5	3	111.18	NC(N/N=C(/C=C\c(cc1)cc(F)c1Cl)\c1nc(cccc2)c2[nH]1)=S
CHEMBL44723	457.46	3.59	7	3	108.39	OC([C@@H](Cc(cc1)ccc1NC(c1ccnc2ccccc12)=O)NC(c(cccc1)c1F)=O)=O
CHEMBL4471914	375.51	3.18	5	4	89.79	CC(C)c(cc(CC[C@H](C(C)(C)CCC1)[C@]11C(NCCO)=O)c1c1O)c1O
CHEMBL4467588	391.59	3.63	4	3	83.55	C[C@@H](CCC(N)=O)[C@H](CC1)[C@@](C)(CC[C@@H]2[C@@](C)(CC[C@@H](C3)O)[C@@H]3C3)[C@@H]1[C@H]2[C@@H]3O
CHEMBL44649	473.92	4.10	7	3	108.39	OC([C@H](Cc(cc1)ccc1NC(c1ccnc2ccccc12)=O)NC(c(cccc1)c1Cl)=O)=O
CHEMBL4464925	396.76	2.87	6	3	86.88	O=C(CC(Nc(cc1)cc(C(F)(F)F)c1Cl)=O)Nc1cc([nH]nc2)c2cc1
CHEMBL4463149	446.89	3.22	7	4	113.32	Nc(cc(cc1)Cl)c1NC(c1ccc(CNC(/C=C(/c2c3cccc2)\NC3=O)=O)cc1)=O
CHEMBL4460974	483.69	4.84	5	4	89.79	C[C@H](CCC(Nc1ccccc1)=O)[C@@H](CC[C@H]1[C@@H]([C@H](C2)[C@@](C)(CC[C@@H](C3)O)[C@H]3C3)[C@@H]3O)[C@]1(C)[C@H]2O
CHEMBL44606	493.56	4.16	7	3	108.39	OC([C@H](Cc(cc1)ccc1NC(c1ccnc2ccccc12)=O)NC([C@H](CC1)Cc2c1cccc2)=O)=O
CHEMBL4458718	405.62	4.70	4	3	69.56	C[C@H](CCC(NO)=O)[C@@H](CC1)[C@](C)(CC2)[C@@H]1[C@@](C)(CC1)[C@H]2[C@](C)(CC2)[C@@H]1C[C@@H]2O
CHEMBL4458648	362.50	4.88	4	3	81.07	S=C(NCc1ccccc1)NNc1c(CCCC2)c2nc2ccccc12
CHEMBL44532	481.55	4.52	7	3	108.39	Cc1cc(C)c(C(N[C@H](Cc(cc2)ccc2NC(c2ccnc3ccccc23)=O)C(O)=O)=O)c(C)c1
CHEMBL4452275	399.41	2.97	8	4	111.69	O=C(c(cc1)ccc1NC(Nc1ccccc1)=O)N/N=C(/c(cccc1)c1N1)\C1=O
CHEMBL4449646	364.83	4.01	4	3	61.36	O[C@@](Cc1ccccc1)(c(cc(cc1)Nc(cccc2)c2Cl)c1N1)C1=O
CHEMBL4446803	392.67	6.15	3	2	49.33	OC(c(cccc1)c1Nc(c(Cl)cc(-c(cc1)ccc1Cl)c1)c1Cl)=O
CHEMBL4446210	409.41	4.58	4	2	57.78	Cc(ccc(C(NCc1cccc(C(F)(F)F)c1)=O)c1)c1-c1cc([nH]nc2)c2cc1
CHEMBL4445949	444.49	4.15	6	3	90.64	O=C(c1c[nH]c2c1cccc2)c1c2[nH]c(cccc3)c3c2cc(C(NCc2ccccc2)=O)n1
CHEMBL4445922	392.50	2.28	5	3	78.43	OC(c1cc(NC(C2CCCC2)=O)cc(-c2ccc(C3CCNCC3)cc2)c1)=O
CHEMBL4445395	379.46	5.52	4	2	54.02	Cc1nccc(-c(cc2)ccc2NC(Nc(cc2)ccc2-c2ccccc2)=O)c1
CHEMBL4443643	369.47	5.21	4	2	57.78	CC(C)c1cccc(NC(c2cc(-c3cc([nH]nc4)c4cc3)c(C)cc2)=O)c1
CHEMBL4443140	434.92	3.32	5	3	78.43	OC(c1cc(NC(c(cc2)ccc2Cl)=O)cc(-c2ccc(C3CCNCC3)cc2)c1)=O

CHEMBL4441808	398.38	4.26	4	3	61.36	O[C@@](Cc1ccccc1)(c(cc(cc1)Nc2ccc(C(F)(F)F)cc2)c1N1)C1=O
CHEMBL4441367	416.35	2.63	6	4	98.66	Oc(cc1)ccc1C(N[C@@H](C(NO)=O)c(cc1)ccc1-c(cc1F)cc(F)c1F)=O
CHEMBL4439636	388.63	5.95	2	2	40.46	CC(C)(CCC[C@@H](CC1)[C@](C)(CC2)[C@H]1[C@H]1[C@@H]2[C@@](C)(CC[C@](C)(C2)O)C2=CC1)O
CHEMBL4436844	458.48	3.43	5	3	78.43	ONC([C@H](c(cc1)ccc1-c(cc1F)cc(F)c1F)NC(C1(C[C@H](C2)C3)C[C@H]3C[C@H]2C1)=O)=O
CHEMBL4436488	366.81	3.61	6	3	83.12	O=C(c1ncccc1)Nc1cccc(NC(Nc2cccc(Cl)c2)=O)c1
CHEMBL4436034	449.26	4.64	5	3	78.43	CC(C)[C@H](C(Nc(ccc(C(F)(F)F)c1)c1Cl)=O)NC(c(cc(cc1)Cl)c1O)=O
CHEMBL4435677	372.25	5.89	3	2	49.33	Cc(cc1)ccc1-c(cc1Cl)cc(Cl)c1Nc(cccc1)c1C(O)=O
CHEMBL4434841	404.53	4.85	4	3	87.82	Oc1cc(CCN([C@@H]2c3ccccc3)C(NCCc3ccccc3)=S)c2cc1O
CHEMBL4434818	419.57	3.41	6	3	71.46	Cc(cc1)ccc1NC(NC1(CCCCC1)C(N(CC1)CCN1c1ccccc1)=O)=N
CHEMBL44338	515.63	4.22	7	3	86.36	O=C(CN(CC1)CCC1NC(Nc1ccccc1)=O)N[C@H](CC1)[C@@H](Cc2cnccc2)c(cc2)c1cc2F
CHEMBL437195	411.46	3.11	6	3	73.05	O=C(NCc(c(F)c1F)ccc1N1[C@@H]2CCC[C@H]1CC2)Nc1c(cn[nH]2)c2ccc1
CHEMBL43682	514.70	3.29	7	3	104.62	O=C(NC[C@@H](Cc1c(C2)cccc1)N2C(NC[C@H]1N(Cc2ncccc2)CCC1)=S)Nc1ccccc1
CHEMBL434359	386.57	5.23	3	3	60.69	C[C@H](CC#CC(C)(C)O)[C@@H](CC1)C(C)(C)[C@@H]1/C=C\C=C(\C[C@H](C[C@@H]1O)O)/C1=C
CHEMBL4303615	428.65	5.93	3	3	60.69	C[C@@H]([C@H](CC1)[C@](C)(CCC2)[C@@H]1/C\2=C/C=C(\C[C@H](C[C@H]1O)O)/C1=C)/C=C/[C@H](C)C(C)(C)O
CHEMBL4302818	413.52	4.33	5	3	66.15	Cc1c(C(NCc2cccc3ccccc23)=O)[nH]c2c1c(OC1CCNCC1)ccc2
CHEMBL43017	505.64	2.52	8	3	127.97	NC(c1ccc(CN(C(N2CCCCCC2)=O)NS(c(cc2)ccc2-c2ccccc2)(=O)=O)cc1)=N
CHEMBL4299936	399.49	3.98	5	3	66.15	O=C(c1cc(c(OC2CCNCC2)ccc2)c2[nH]1)NCc1cccc2ccccc12
CHEMBL428939	412.28	4.63	5	3	78.01	Oc1ccc(C(NCc2ccccc2)=O)c2c1[nH]c(-c(ccc(Cl)c1)c1Cl)n2
CHEMBL422411	408.46	3.16	6	3	86.35	O=C(Cc1c[nH]c2c1cccc2)N[C@@H]1N=C(c2ccccc2)c(cccc2)c2NC1=O
CHEMBL421520	468.66	3.33	6	3	129.75	CC(C)(C)N/C(\S)=N/C[C@H](Cc1c(C2)cccc1)N2C([C@H](Cc(c(C)c1)c(C)cc1O)N)=O
CHEMBL414867	372.47	3.83	5	3	91.86	CC(C)(C)c(cc1)ccc1NC(c(cc1)ccc1-c1ncccc1C(N)=N)=O
CHEMBL41024	388.59	5.56	3	3	60.69	CCC(CC)(C#CC[C@@](C)(CC1)C(C)(C)[C@H]1/C=C\C=C(/C[C@@H](C1)O)\C[C@H]1O)O
CHEMBL40581	503.67	3.37	7	3	104.87	O=C(NC[C@H](Cc1c(C2)cccc1)N2C(NC[C@H]1N(Cc2c[o]cc2)CCC1)=S)Nc1ccccc1
CHEMBL40528	519.74	4.02	6	3	119.97	O=C(NC[C@@H](Cc1c(C2)cccc1)N2C(NC[C@@H]1N(Cc2c[s]cc2)CCC1)=S)Nc1ccccc1
CHEMBL404860	352.82	2.59	5	3	83.90	NC(NC(C[n]1c(-c(cccc2)c2Cl)ccc1-c1ccccc1)=O)=N
CHEMBL404127	396.53	4.04	5	3	78.43	OC(C[C@H](C1CCCCC1)N1)=C(C(Nc2ccc(C3CCCCC3)cc2)=O)C1=O
CHEMBL401697	401.31	3.19	5	3	69.81	Cc([nH]c(c1nc(C(NC)=O)c2)c2NCc2c(C)cccc2C)c1Br
CHEMBL401496	368.44	3.18	5	3	83.90	NC(NC(C[n]1c(-c2cc3ccccc3cc2)ccc1-c1ccccc1)=O)=N
CHEMBL40145	460.70	2.61	6	3	101.45	N[C@H](C(C1CCCCC1)C1CCCCC1)C(N(CCC1)[C@@H]1C(NC[C@H](CC1)CC[C@H]1N)=O)=O
CHEMBL399837	387.27	3.20	5	3	83.90	NC(NC(C[n]1c(-c(cc(cc2)Cl)c2Cl)ccc1-c1ccccc1)=O)=N
CHEMBL399718	368.44	3.18	5	3	83.90	NC(NC(C[n]1c(-c2cccc3ccccc23)ccc1-c1ccccc1)=O)=N
CHEMBL399031	370.81	2.69	5	3	83.90	NC(NC(C[n]1c(-c2cccc(Cl)c2)ccc1-c(cc1)ccc1F)=O)=N
CHEMBL398442	402.88	3.78	5	3	83.90	NC(NC(C[n]1c(-c(cccc2)c2Cl)ccc1-c1cc2ccccc2cc1)=O)=N

CHEMBL398441	431.72	3.32	5	3	83.90	NC(NC(C[n]1c(-c(cccc2)c2Cl)ccc1-c1cccc(Br)c1)=O)=N
CHEMBL398352	386.43	3.28	5	3	83.90	NC(NC(C[n]1c(-c2cc3ccccc3cc2)ccc1-c1cccc(F)c1)=O)=N
CHEMBL398330	370.81	2.69	5	3	83.90	NC(NC(C[n]1c(-c(cccc2)c2Cl)ccc1-c1cc(F)ccc1)=O)=N
CHEMBL398314	366.85	2.93	5	3	83.90	Cc(cc1)ccc1-c1ccc(-c(cccc2)c2Cl)[n]1CC(NC(N)=N)=O
CHEMBL396960	409.92	3.52	6	3	73.05	O=C(NCc(cc1)cc(Cl)c1N1[C@@H]2CCC[C@H]1CC2)Nc1c(cn[nH]2)c2ccc1
CHEMBL396714	409.92	3.52	6	3	73.05	O=C(NCc(ccc(N1[C@@H]2CCC[C@@H]1CC2)c1)c1Cl)Nc1c(cn[nH]2)c2ccc1
CHEMBL396713	399.44	3.10	6	3	73.05	O=C(NCc(cc1F)cc(F)c1N1CCCCCC1)Nc1c(cn[nH]2)c2ccc1
CHEMBL395262	411.46	3.11	6	3	73.05	O=C(NCc(cc1F)cc(F)c1N1[C@@H]2CCC[C@H]1CC2)Nc1c(cn[nH]2)c2ccc1
CHEMBL395055	375.21	3.47	6	3	86.88	O=C(c1n[nH]c(NC(c(cccc2)c2Cl)=O)c1Cl)Nc1ccccc1
CHEMBL394120	422.22	3.35	5	3	74.35	Oc(cc1)cc2c1[nH]cc2CCNC(Oc(cc1)ccc1I)=O
CHEMBL390222	372.51	3.92	4	3	66.73	C[C@H](CC1)CC[C@H]1NCc(cc1)cc(C2)c1-c1c2c(-c(cc2)ccc2N)n[nH]1
CHEMBL388853	400.48	3.22	6	3	86.88	O=C(c(cc1)ccc1-c1nccc(-c2cc(C(NCC3)=O)c3[nH]2)c1)NC1CCCC1
CHEMBL388032	431.46	3.74	6	3	73.05	O=C(NCc(cc1)cc(C(F)(F)F)c1N1CCCCCC1)Nc1c(cn[nH]2)c2ccc1
CHEMBL387137	400.85	4.46	3	3	87.34	C[C@@](CSc(cc1)ccc1N)(c1cc2cc(Cl)c(C(F)(F)F)cc2[nH]1)O
CHEMBL382532	381.26	3.28	6	3	86.88	O=C(c1c(C(NC2CCCCC2)=O)nc[nH]1)Nc(cc1)cc(Cl)c1Cl
CHEMBL374662	423.52	2.60	6	3	76.81	O=C1Nc(ccc(-c2c[nH]nc2)c2)c2C=C1c1cc(cc(CN2CCCCC2)cc2)c2[nH]1
CHEMBL373616	360.41	4.21	5	3	86.21	CC(C)c1c2nc(Cc3c[nH]c4c3cccc4)c(O)c(C(O)=O)c2ccc1
CHEMBL367999	356.38	3.90	6	3	86.88	O=C(c1ccccc1)c(cc1)cc2c1[nH]c(NC(Nc1ccccc1)=O)n2
CHEMBL36510	400.60	5.57	3	3	60.69	C[C@H](CC#CC(C)(C)O)[C@@H](CCC1)C(C)(C)[C@@H]1/C=C\C=C(/C[C@@H](C[C@@H]1O)O)\C1=C
CHEMBL364580	436.52	3.42	7	4	112.58	CC(C)(C(Nc(cc1)ccc1-[n]1c(cccc2)c2nc1)=O)c1c[nH]c(cc2)c1cc2C(N)=N
CHEMBL362500	424.46	4.92	6	4	98.66	Oc(cccc1)c1NC(c(cc1)ccc1-c(cc1)ccc1C(Nc(cccc1)c1O)=O)=O
CHEMBL360032	373.41	3.30	6	3	79.46	COc1cccc(NC(Nc(cc2)ccc2-c2cccc3c2CNC3=O)=O)c1
CHEMBL358910	373.46	2.96	5	3	110.03	NC(c1ccc(/C=C(\CCCC/C2=C\c(cc3)ccc3C(N)=O)/C/2=O)cc1)=N
CHEMBL356630	386.50	3.08	5	3	105.32	C/N=C(/c1ccc(/C=C(/CCCC/C2=C\c(cc3)ccc3C(N)=N)\C/2=O)cc1)\N
CHEMBL356466	400.52	3.17	5	3	94.03	CN(C)C(c1ccc(/C=C(\CCCC/C2=C\c(cc3)ccc3C(N)=N)/C/2=O)cc1)=N
CHEMBL355448	374.37	4.00	6	3	86.88	O=C(c1ccccc1)c(cc1)cc2c1[nH]c(NC(Nc(cccc1)c1F)=O)n2
CHEMBL350197	421.03	6.33	4	1	126.71	C/C(\C1(C[C@H](C2)C3)C[C@H]3C[C@H]2C1)=N\N=C(/N/N=C(\c(cc1)ccc1Cl)/S)\S
CHEMBL344154	426.45	3.07	6	3	86.35	O=C(c1cc(cccc2)c2[nH]1)NC[C@H](C(Nc1c2cccc1)=O)N=C2c(cccc1)c1F
CHEMBL338753	434.28	2.41	7	4	107.53	CC(Nc(cc1)ccc1NC(/C=C(\C[C@H](C(O)=O)Nc1cc(Cl)c2)/c1c2Cl)=O)=O
CHEMBL336242	476.97	3.24	8	4	102.15	O=C(Nc1cc(CN[C@H](C2)Cc3c2ccc(Cl)c3)n[nH]1)Nc1c([C@@H](CCC2)N2C2=O)c2ccc1
CHEMBL3355431	480.37	3.05	6	4	104.45	N[C@@H]([C@H]([C@H](c(ccc(Cl)c1)c1N1)C1=O)c1cccc(Cl)c1F)C(N[C@H](CC1)CC[C@H]1O)=O
CHEMBL3347649	401.31	3.41	5	3	69.81	CC(C)(C)NCc1cc2cc(NC(c(cccc3)c3Br)=O)cnc2[nH]1
CHEMBL3342086	392.62	4.65	3	3	60.69	CC[C@H]([C@@H](C[C@H](CC1)O)[C@]1(C)[C@H](CC1)[C@@H]2[C@@H](CC3)[C@@]1(C)[C@@H]3[C@@H](C)CCO)[C@@H]2O

CHEMBL3342084	404.63	5.01	3	3	60.69	C[C@@H](CCCO)[C@@H](CC1)[C@](C)(CC[C@H]2[C@@](C)(CC[C@H](C3)O)[C@H]3/C3=C/C)[C@H]1[C@@H]2[C@H]/3O
CHEMBL3342079	378.59	4.38	3	3	60.69	C[C@@H](CCCO)[C@H](CC1)[C@](C)(CC[C@@H]2[C@@](C)(CC[C@H](C3)O)[C@@H]3C3)[C@@H]1[C@H]2[C@H]3O
CHEMBL3342077	378.59	4.38	3	3	60.69	C[C@@H](CCCO)[C@H](CC1)[C@@](C)(CC[C@@H]2[C@](C)(CC[C@@H](C3)O)[C@H]3C3)[C@H]1[C@H]2[C@H]3O
CHEMBL3341876	380.83	4.21	6	3	83.12	CC(Nc1cccc(-c2cccc(NC(Nc(cc3)ccc3Cl)=O)c2)n1)=O
CHEMBL3341866	352.82	4.15	5	3	66.05	CNc1cccc(-c2cccc(NC(Nc(cc3)ccc3Cl)=O)c2)n1
CHEMBL3339382	418.43	2.71	8	4	115.56	Cc1n[nH]c(-c(cc(C)cc2)c2NC(c2c(C(Nc(cc3)ccc3F)=O)[nH]cn2)=O)c1
CHEMBL3335248	457.12	3.06	6	4	82.62	CCOc(c(O)c1CNc(cc2)cc(N3)c2NC3=O)cc(Br)c1Br
CHEMBL3335247	443.09	2.65	6	4	82.62	COc(c(O)c1CNc(cc2)cc(N3)c2NC3=O)cc(Br)c1Br
CHEMBL3330955	420.58	3.48	5	3	98.14	C=CCNC(NNC(c1nc2ccccc2c(C2(C[C@H](C3)C4)C[C@H]4C[C@H]3C2)c1)=O)=S
CHEMBL3329665	430.46	2.65	8	4	112.32	Cc1c(/C=C(/c(ccc(NC(NC(c(cc2)ccc2OC)=O)=O)c2)c2N2)\C2=O)[nH]c(C)c1
CHEMBL3329653	402.41	1.91	8	4	112.32	COc(cc1)ccc1C(NC(Nc1cccc(/C2=C/c3ccc[nH]3)c1NC/2=O)=O)=O
CHEMBL3329652	402.41	1.91	8	4	112.32	COc(cc1)ccc1C(NC(Nc(cc1)cc(NC2=O)c1/C\2=C\c1ccc[nH]1)=O)=O
CHEMBL3329651	402.41	1.91	8	4	112.32	COc(cc1)ccc1C(NC(Nc(cc1)cc(/C2=C\c3ccc[nH]3)c1NC/2=O)=O)=O
CHEMBL3329650	402.41	1.91	8	4	112.32	COc(cc1)ccc1C(NC(Nc1cccc(NC2=O)c1/C\2=C/c1ccc[nH]1)=O)=O
CHEMBL3329562	390.28	4.60	5	3	73.72	CC(C)(c1cc(/C(\C)=N\O)ccc1)NC(Nc(cc1)ccc1Br)=O
CHEMBL3329561	380.27	5.09	5	3	73.72	CC(C)(c1cc(/C(\C)=N/O)ccc1)NC(Nc(cc1)cc(Cl)c1Cl)=O
CHEMBL3329302	394.86	4.26	6	3	83.12	CCNC(Nc(nc1)cc(-c2ccccc2)c1C(Nc1cccc(Cl)c1)=O)=O
CHEMBL332597	419.50	6.44	6	3	133.08	Cc(cc1)c(C)cc1-c1cccc(/C=N/N=C(\Nc2cc(C(O)=O)ccc2)/S)c1O
CHEMBL3325563	410.60	5.27	3	3	60.69	C[C@@H](CC#CC(C)(C)O)[C@@H](CC1)[C@](C)(CCC2)[C@H]1/C\2=C\c1c(CO)c(CO)ccc1
CHEMBL3325561	410.60	5.27	3	3	60.69	C[C@@H](CC#CC(C)(C)O)[C@H](CC1)[C@@](C)(CCC2)[C@H]1/C\2=C/c1cc(CO)c(CO)cc1
CHEMBL3322584	452.47	0.90	8	4	112.32	COc(cc1)ccc1C1=CNC=C(C(Nc(cc2)cc(NC3=O)c2/C\3=C\c2ccc[nH]2)=O)C1=O
CHEMBL3322177	358.40	4.12	6	3	79.04	CNC(Nc1nc(cc(cc2)Oc3cc(-c4ccccc4)ccc3)c2[nH]1)=O
CHEMBL3318817	358.44	4.05	5	3	83.27	CC(C)Cc(cc1)ccc1-c([nH]c(N=C(N)N1)c2C1=O)c2-c1ccccc1
CHEMBL3317853	398.38	5.15	4	2	58.20	Cc1cccc(C(Nc2cccc(NC(c3ccc(C(F)(F)F)cc3)=O)c2)=O)c1
CHEMBL3317850	372.47	5.54	4	2	58.20	CC(C)(C)c(cc1)ccc1C(Nc1cc(NC(c2ccccc2)=O)ccc1)=O
CHEMBL3317849	384.36	4.80	4	2	58.20	O=C(c1ccccc1)Nc1cccc(NC(c2ccc(C(F)(F)F)cc2)=O)c1
CHEMBL3317822	350.80	4.56	4	2	58.20	O=C(c1ccccc1)Nc1cccc(NC(c(cc2)ccc2Cl)=O)c1
CHEMBL3311219	418.66	5.47	3	3	60.69	C[C@@H](CCCC(C)(C)O)[C@H](CC1)[C@](C)(CC[C@H]2[C@](C)(CC[C@H](C3)O)C3=C3)[C@@H]1[C@@H]2[C@@H]3O
CHEMBL3311217	436.67	4.75	4	4	80.92	C[C@H](CCCC(C)(C)O)[C@@H](CC1)[C@](C)(CC2)[C@@H]1[C@@H](C[C@@H]1O)[C@@H]2[C@](C)(CC[C@H](C2)O)[C@@]12O
CHEMBL3311192	344.28	3.91	3	2	41.13	CCNc1cc(C(F)(F)F)cc(NC(c(c(F)ccc2)c2F)=O)c1
CHEMBL3309280	358.31	4.27	3	2	41.13	CC(C)Nc1cc(C(F)(F)F)cc(NC(c(c(F)ccc2)c2F)=O)c1
CHEMBL329269	420.55	4.73	5	3	78.43	CC(C)(C)NC(c1c(C[C@H](C#Cc(cccc2)c2C(NC(C)(C)C)=O)O)cccc1)=O
CHEMBL323654	446.05	3.20	6	4	115.06	OC(c1cc(Br)cc(Cc2cc(Br)cc(C(O)=O)c2O)c1O)=O

CHEMBL323387	410.52	4.07	5	3	91.33	NC(c1ccc(cc(cc2)C(Nc3cc(C(C4CCCC4)=NCC4)c4cc3)=O)c2c1)=N
CHEMBL319047	355.40	3.48	5	3	92.11	NC(c1ccc(cc(cc2-c3c[o]cc3)C(Nc3ccccc3)=O)c2c1)=N
CHEMBL315162	359.49	4.09	5	3	98.47	CC(C)(C)c1cc(NC(Nc2ccc(C)cc2)=O)c(CNC(C)=O)[s]1
CHEMBL306343	412.46	5.35	6	2	151.63	C/C(\c1ncccc1)=N/N=C(/N/N=C(/Nc1c(C(F)(F)F)cccc1)\S)\S
CHEMBL305349	370.84	2.99	6	4	108.01	NC(c(cc1[nH]c(-c(cccc2OC3CCCC3)c2O)nc1c1)c1Cl)=N
CHEMBL300118	394.26	1.99	6	4	104.45	Nc1ccc(CC(N[C@@H](C[C@@H](C(O)=O)Nc2cc(Cl)c3)c2c3Cl)=O)cc1
CHEMBL300046	393.29	4.12	4	2	100.49	OC(CC([C@H](c(cc1)ccc1Cl)N1)=C(c(cc2)ccc2Cl)N=C1S)=O
CHEMBL293892	382.44	3.10	6	4	108.01	C[C@H](CCCC1)[C@@H]1Oc1cccc(-c([nH]c2c3)nc2cc(C(N)=N)c3F)c1O
CHEMBL293283	444.65	5.07	4	4	80.92	C[C@H]([C@@H](CC1)[C@@](C)(CCC2)[C@@H]1/C\2=C/C=C(/C[C@H](C[C@@H]1O)O)\C1=C)/C=C\[C@@](C)(C(C)(C)O)O
CHEMBL291877	413.69	3.27	5	3	78.43	OC([C@@H](C1)Nc2cc(Cl)cc(Cl)c2[C@H]1NC(Cc(cc1)ccc1Cl)=O)=O
CHEMBL290789	400.60	5.57	3	3	60.69	C[C@@H](CC#CC(C)(C)O)[C@H](CCC1)C(C)(C)[C@@H]1/C=C\C=C(\C[C@@H](C[C@@H]1O)O)/C1=C
CHEMBL28550	376.30	5.49	2	2	40.46	Oc(cc1)ccc1/C(\CC(F)(F)F)=C(\CC(F)(F)F)/c(cc1)ccc1O
CHEMBL272360	411.35	4.38	4	3	87.82	Oc(c(O)c(c(C1)c2CCN1C(NCCCc1ccccc1)=S)Cl)c2Cl
CHEMBL272054	390.48	3.88	5	3	78.43	OC(C[C@H](c1ccccc1)N1)=C(C(Nc2ccc(C3CCCCC3)cc2)=O)C1=O
CHEMBL271845	390.48	3.88	5	3	78.43	OC(C[C@@H](c1ccccc1)N1)=C(C(Nc2ccc(C3CCCCC3)cc2)=O)C1=O
CHEMBL271291	390.48	3.88	5	3	78.43	OC(C[C@H](c1ccccc1)N1)=C(C(Nc2ccc(C3CCCCC3)cc2)=O)C1=O
CHEMBL270703	394.59	3.53	4	4	80.92	C[C@H](CCCO)[C@H](CC[C@H]1[C@@H]([C@@H](C2)[C@](C)(CC[C@@H](C3)O)[C@H]3C3)[C@@H]3O)[C@]1(C)[C@@H]2O
CHEMBL27049	365.82	4.50	4	2	66.40	C/C(\c1cc2ccccc2cc1)=C(/C(Nc(cccc1)c1C(O)=O)=O)\Cl
CHEMBL270287	378.59	4.38	3	3	60.69	C[C@H](CCCO)[C@H](CC1)[C@](C)(CC[C@H]2[C@@](C)(CC[C@H](C3)O)[C@H]3C3)[C@H]1[C@H]2[C@H]3O
CHEMBL268731	390.42	1.74	6	3	86.88	Cc1c(/C=C(/c(cc(cc2)F)c2N2)\C2=O)[nH]c(C)c1C(NCc1ccncc1)=O
CHEMBL259707	382.47	2.08	6	4	117.04	Cc1c(C)c(-c(cc2)ccc2C(N)=N)c[n]2c1nc(-c(cc1)ccc1C(N)=N)c2
CHEMBL254706	387.27	3.20	5	3	83.90	NC(NC(C[n]1c(-c(cccc2)c2Cl)ccc1-c1cc(Cl)ccc1)=O)=N
CHEMBL254694	387.27	3.20	5	3	83.90	NC(NC(C[n]1c(-c(cccc2)c2Cl)ccc1-c(cccc1)c1Cl)=O)=N
CHEMBL254486	382.47	3.52	5	3	83.90	Cc1ccc(cc(cc2)-c3ccc(-c4ccccc4)[n]3CC(NC(N)=N)=O)c2c1
CHEMBL254477	420.82	3.44	5	3	83.90	NC(NC(C[n]1c(-c(cccc2)c2Cl)ccc1-c1ccc(C(F)(F)F)cc1)=O)=N
CHEMBL254476	386.38	2.83	5	3	83.90	NC(NC(C[n]1c(-c2ccc(C(F)(F)F)cc2)ccc1-c1ccccc1)=O)=N
CHEMBL253885	416.91	4.13	5	3	83.90	Cc1ccc(cc(cc2)-c3ccc(-c(cccc4)c4Cl)[n]3CC(NC(N)=N)=O)c2c1
CHEMBL253875	418.50	4.37	5	3	83.90	NC(NC(C[n]1c(-c2cccc3ccccc23)ccc1-c1cc2ccccc2cc1)=O)=N
CHEMBL253437	382.47	3.52	5	3	83.90	Cc(cc1)ccc1-c1ccc(-c2cccc3ccccc23)[n]1CC(NC(N)=N)=O
CHEMBL253236	421.71	3.80	5	3	83.90	NC(NC(C[n]1c(-c(cc(cc2)Cl)c2Cl)ccc1-c(cccc1)c1Cl)=O)=N
CHEMBL253235	366.85	2.93	5	3	83.90	Cc1cccc(-c2ccc(-c(cccc3)c3Cl)[n]2CC(NC(N)=N)=O)c1
CHEMBL253231	402.88	3.78	5	3	83.90	NC(NC(C[n]1c(-c2cccc3ccccc23)ccc1-c1cccc(Cl)c1)=O)=N
CHEMBL253229	402.88	3.78	5	3	83.90	NC(NC(C[n]1c(-c(cccc2)c2Cl)ccc1-c1cccc2ccccc12)=O)=N

CHEMBL253207	400.46	3.62	5	3	83.90	Cc1ccc(cc(cc2)-c3ccc(-c(cc4)ccc4F)[n]3CC(NC(N)=N)=O)c2c1
CHEMBL252989	404.39	4.87	5	3	74.35	Oc(cc1)cc2c1[nH]c1c2[C@@H](CNC(c(cc2)ccc2OC(F)(F)F)=O)CCC1
CHEMBL252828	405.26	3.30	5	3	83.90	NC(NC(C[n]1c(-c(cc(cc2)Cl)c2Cl)ccc1-c1cc(F)ccc1)=O)=N
CHEMBL252626	370.81	2.69	5	3	83.90	NC(NC(C[n]1c(-c(cccc2)c2Cl)ccc1-c(cc1)ccc1F)=O)=N
CHEMBL252423	370.81	2.69	5	3	83.90	NC(NC(C[n]1c(-c2cccc(Cl)c2)ccc1-c1cc(F)ccc1)=O)=N
CHEMBL252385	397.28	2.71	5	3	83.90	NC(NC(C[n]1c(-c2cccc(Br)c2)ccc1-c1ccccc1)=O)=N
CHEMBL252182	352.82	2.59	5	3	83.90	NC(NC(C[n]1c(-c2cccc(Cl)c2)ccc1-c1ccccc1)=O)=N
CHEMBL251992	417.51	2.93	7	4	113.00	CC(C)(C)C(Nc(cc1)ccc1-c1ccc(-c2ccccc2)[n]1CC(NC(N)=N)=O)=O
CHEMBL251828	405.26	3.30	5	3	83.90	NC(NC(C[n]1c(-c(cc(cc2)Cl)c2Cl)ccc1-c(cc1)ccc1F)=O)=N
CHEMBL251806	400.40	3.18	5	3	83.90	Cc1cccc(-c2ccc(-c3ccc(C(F)(F)F)cc3)[n]2CC(NC(N)=N)=O)c1
CHEMBL249044	397.28	2.51	5	3	83.80	NCCCc(c(c1c2)ccc2-c2c[nH]nc2)cc(C(Br)=CN2)c1C2=O
CHEMBL248761	423.52	5.38	5	3	74.25	Oc1cc(CC[C@H]([C@@H]2Cc3ccccc3)NC(Nc3cccc4cnccc34)=O)c2cc1
CHEMBL24754	508.51	1.21	8	3	125.98	Cc(cc1)ccc1-c1ccc(CN(CCCC[C@H]2NC(c(cc3)ccc3OP(O)(O)=O)=O)C2=O)cc1
CHEMBL246815	399.46	1.65	6	4	84.83	C[C@H]([C@@H](c(cc1)cc(CN2)c1NC2=O)O)N(CC1)CCC1(c(cc1)ccc1F)O
CHEMBL242738	389.45	3.98	6	3	95.08	CC(C)(C)CNC(c(cc1)cc(C2)c1-c1c2c(-c(cc2)ccc2C(O)=O)n[nH]1)=O
CHEMBL242736	373.50	4.25	4	3	60.94	C[C@H](CC1)CC[C@H]1NCc(cc1)cc(C2)c1-c1c2c(-c(cc2)ccc2O)n[nH]1
CHEMBL242565	354.80	3.20	6	3	86.88	Cc1c(NC(c(cccc2)c2Cl)=O)[nH]nc1C(Nc1ccccc1)=O
CHEMBL242523	417.46	3.04	7	4	115.31	O[C@H](CC1)CC[C@H]1NC(c(cc1)cc(C2)c1-c1c2c(-c(cc2)ccc2C(O)=O)n[nH]1)=O
CHEMBL241138	422.22	3.35	5	3	74.35	Oc(cc1)cc2c1[nH]cc2CCNC(Oc1cccc(I)c1)=O
CHEMBL240274	364.32	3.77	5	3	74.35	Oc(cc1)cc2c1[nH]cc2CCNC(Oc1cccc(C(F)(F)F)c1)=O
CHEMBL232805	411.46	3.64	6	4	98.24	OCCNC(c1cc(-c2c(C3)c(-c(cc4)ccc4-c(cc4)ccc4O)n[nH]2)c3cc1)=O
CHEMBL231519	375.48	2.91	6	3	73.05	O=C(NCc(cc1)ccc1N1[C@@H]2CCC[C@H]1CC2)Nc1c(cn[nH]2)c2ccc1
CHEMBL231436	389.50	3.25	6	3	73.05	Cc(cc(CNC(Nc1c(cn[nH]2)c2ccc1)=O)cc1)c1N1[C@@H]2CCC[C@H]1CC2
CHEMBL231422	381.45	3.00	6	3	73.05	O=C(NCc(cc1)cc(F)c1N1CCCCCC1)Nc1c(cn[nH]2)c2ccc1
CHEMBL231227	393.47	3.01	6	3	73.05	O=C(NCc(cc1)cc(F)c1N1[C@@H]2CCC[C@H]1CC2)Nc1c(cn[nH]2)c2ccc1
CHEMBL230919	411.46	3.11	6	3	73.05	O=C(NCc(c(F)c1)cc(F)c1N1[C@@H]2CCC[C@H]1CC2)Nc1c(cn[nH]2)c2ccc1
CHEMBL230485	431.46	3.74	6	3	73.05	O=C(NCc(cc1)c(C(F)(F)F)cc1N1CCCCCC1)Nc1c(cn[nH]2)c2ccc1
CHEMBL230380	397.91	3.50	6	3	73.05	O=C(NCc(ccc(N1CCCCCC1)c1)c1Cl)Nc1c(cn[nH]2)c2ccc1
CHEMBL226261	414.51	3.57	6	3	86.88	O=C(c(cc1)ccc1-c1nccc(-c2cc(C(NCC3)=O)c3[nH]2)c1)NC1CCCCC1
CHEMBL226021	408.73	5.98	3	3	60.69	OB(/C=C/c(cc1)cc(Cl)c1-c(cc1)cc(C2(C[C@H](C3)C4)C[C@H]4C[C@H]3C2)c1O)O
CHEMBL221927	428.53	4.12	6	3	86.88	CC(Nc(cc1)ccc1-c1nc(cc(cc2)C(NC3(C[C@H](C4)C5)C[C@H]5C[C@H]4C3)=O)c2[nH]1)=O
CHEMBL221735	386.50	2.00	5	3	74.15	NCC1CCN(Cc(cc2)cc3c2[nH]c(C2=Cc(cccc4)c4NC2=O)c3)CC1
CHEMBL221496	423.52	2.70	6	3	76.81	O=C1Nc(ccc(-c2n[nH]cc2)c2)c2C=C1c1cc(cc(CN2CCCCC2)cc2)c2[nH]1

CHEMBL221448	400.48	2.14	6	3	91.22	NC(c(cc1)cc(C=C2c3cc(cc(CN4CCCCC4)cc4)c4[nH]3)c1NC2=O)=O
CHEMBL219167	385.84	5.14	2	2	61.32	C[C@@](CSc1ccccc1)(c1cc2cc(Cl)c(C(F)(F)F)cc2[nH]1)O
CHEMBL216713	403.56	2.95	5	3	86.63	C[C@H]([C@@H](CC1)[C@](C)(CC2)[C@@H]1[C@H]1[C@H]2[C@](C)(CC[C@@H](C2)O)C2=CC1)C(NCC(O)=O)=O
CHEMBL216337	356.49	4.28	5	3	94.58	CCc1c(C)[nH]c(CCC2)c1/C\2=N/NC(Nc(cc1)ccc1SC)=O
CHEMBL216145	402.42	4.27	5	3	80.04	CCCc1cc(N)c(cc(cc2)NC(NCc3ccc(C(F)(F)F)cc3)=O)c2n1
CHEMBL214445	444.92	5.02	6	3	86.88	O=C(C1CCC1)Nc(cc1)cc2c1[nH]c(-c(cc1)ccc1NC(c(cccc1)c1Cl)=O)n2
CHEMBL212336	448.88	4.53	6	3	86.88	O=C([C@@H](C1)[C@@H]1F)Nc(cc1)cc2c1[nH]c(-c(cc1)ccc1NC(c(cccc1)c1Cl)=O)n2
CHEMBL211156	394.69	5.29	4	2	64.92	Cc(ccc(NC(NC/C(\C#N)=C\c(cc1)cc(Cl)c1Cl)=O)c1)c1Cl
CHEMBL210633	435.49	2.63	7	4	98.64	Oc(c(C1=CC2=CNC=CC2=N1)ccc1)c1-c1cccc(CNC(Nc2cnccc2)=O)c1
CHEMBL210563	412.49	2.98	6	3	76.96	Oc(c(C1=CC2=CNC=CC2=N1)ccc1)c1-c1cccc(CNC(N2CCCC2)=O)c1
CHEMBL210021	380.66	4.94	4	2	64.92	N#C/C(\CNC(Nc(ccc(Cl)c1)c1Cl)=O)=C\c(cccc1)c1Cl
CHEMBL209772	435.49	2.98	7	4	98.64	Oc(c(C1=CC2=CNC=CC2=N1)ccc1)c1-c1cccc(CNC(Nc2ncccc2)=O)c1
CHEMBL209748	415.11	5.55	4	2	64.92	N#C/C(\CNC(Nc(cc1)cc(Cl)c1Cl)=O)=C/c(cc1)cc(Cl)c1Cl
CHEMBL209700	380.66	4.94	4	2	64.92	N#C/C(\CNC(Nc(cc1)cc(Cl)c1Cl)=O)=C/c(cccc1)c1Cl
CHEMBL209377	380.66	4.94	4	2	64.92	N#C/C(\CNC(Nc(cc1)ccc1Cl)=O)=C/c(cc1)cc(Cl)c1Cl
CHEMBL206240	373.45	5.13	4	2	80.39	CC(C)(C)c(cc1)ccc1-c(cc1)ccc1-c1cc(C(N)=O)cc(C(O)=O)c1
CHEMBL202667	373.45	5.13	4	2	80.39	CC(C)(C)c(cc1)ccc1-c1cccc(-c2cc(C(N)=O)cc(C(O)=O)c2)c1
CHEMBL200958	361.19	3.16	5	3	78.43	C=CC(Nc(cc1)cc(O)c1C(Nc1cccc(Br)c1)=O)=O
CHEMBL200834	414.21	4.79	4	2	57.78	O=C(c1c[nH]c(Cc(c(Cl)ccc2)c2Cl)n1)Nc1ccc(C(F)(F)F)cc1
CHEMBL199136	362.82	3.84	5	3	83.80	NC(c1cccc(-c2c(ccc(Nc(cccc3)c3Cl)c3)c3n[nH]2)c1)=O
CHEMBL19894	370.45	5.31	4	2	65.98	Cc1cc(-c2ccc(-c3ccc(C(=O)O)cc3)[nH]2)nc2c(C(C)C)cccc12
CHEMBL198443	409.55	4.03	5	2	117.74	CC(C)(C)c(cc1)ccc1N/C(\S)=N/Cc(cc1)cc(F)c1NS(C)(=O)=O
CHEMBL198419	403.27	3.43	6	3	86.88	C[C@H](c1ccccc1)NC(c1c(C(Nc(cc2)cc(Cl)c2Cl)=O)nc[nH]1)=O
CHEMBL198044	353.49	3.57	5	2	104.76	CC(C)(C)c1ccc(C/N=C(/NCc(cc2)cc3c2[nH]nn3)\S)cc1
CHEMBL197698	363.80	4.24	5	3	78.01	OC(c(cc1)ccc1-c1c(ccc(Nc(cccc2)c2Cl)c2)c2n[nH]1)=O
CHEMBL197613	363.80	4.24	5	3	78.01	OC(c1cccc(-c2c(ccc(Nc(cccc3)c3Cl)c3)c3n[nH]2)c1)=O
CHEMBL197591	403.27	3.43	6	3	86.88	C[C@H](c1ccccc1)NC(c1c(C(Nc(cc2)cc(Cl)c2Cl)=O)nc[nH]1)=O
CHEMBL19727	390.87	5.57	4	2	65.98	CC(C)c(cccc1c(Cl)c2)c1nc2-c1ccc(-c(cc2)ccc2C(O)=O)[nH]1
CHEMBL196852	423.69	3.75	6	3	86.88	O=C(c1c(C(Nc(cc2)cc(Cl)c2Cl)=O)nc[nH]1)NCc(cc1)ccc1Cl
CHEMBL196653	404.45	3.22	7	4	155.55	NC(c1c(NC(N)=O)[s]c(C#Cc2cc(NC(c3ccccc3)=O)ccc2)c1)=O
CHEMBL195919	403.50	3.65	5	2	117.74	CS(Nc(ccc(C/N=C(\Nc1cccc2ccccc12)/S)c1)c1F)(=O)=O
CHEMBL195598	390.83	3.74	6	3	100.87	NC(c(cc1)cc2c1[nH]c(-c(cc1)ccc1C(Nc(cc1)ccc1Cl)=O)n2)=O
CHEMBL194806	445.95	4.44	6	4	81.84	O=C(c1cccc(-c2n[nH]c3c2ccc(Nc(cccc2)c2Cl)c3)c1)NC1CCNCC1

CHEMBL193394	356.38	3.14	6	3	100.87	NC(c(cc1)cc2c1[nH]c(-c(cc1)ccc1C(Nc1ccccc1)=O)n2)=O
CHEMBL192156	425.27	4.35	6	3	100.87	NC(c(cc1)cc2c1[nH]c(-c(cc1)ccc1C(Nc(cc1)cc(Cl)c1Cl)=O)n2)=O
CHEMBL192026	393.87	4.34	5	3	80.14	COc(cc1)cc2c1[nH]c(C(Nc1c(CCCl)c3ccccc3c(N)c1)=O)c2
CHEMBL191485	392.47	5.45	3	2	57.53	OC(/C(\F)=C/c(cc1)ccc1-c(cc1)cc(C2(C[C@H](C3)C4)C[C@H]4C[C@H]3C2)c1O)=O
CHEMBL191402	459.59	4.69	7	3	90.12	CCc1cccc(CC)c1NC(N(C1)Cc2c1c(NC(c1ccc(C(C)(C)C)cc1)=O)n[nH]2)=O
CHEMBL19040	373.52	4.96	5	3	98.47	CC(C)c(cc1)ccc1NC(Nc1c(C(NC)=O)[s]c(C(C)(C)C)c1)=O
CHEMBL18998	403.60	2.69	4	2	76.04	NCCc1c[nH]cn1.S=C(NC1CCCCC1)N(CC1)CCC1c1c[nH]cn1
CHEMBL187730	388.51	5.52	3	2	57.53	C/C(\C(O)=O)=C\c(cc1)ccc1-c(cc1)cc(C2(C[C@H](C3)C4)C[C@H]4C[C@H]3C2)c1O
CHEMBL18752	359.49	4.53	5	3	98.47	CCc(cc1)ccc1NC(Nc1c(C(NC)=O)[s]c(C(C)(C)C)c1)=O
CHEMBL185515	397.56	5.62	3	2	53.11	CC(C)[C@@H]1N(C)CCc2c1cc([C@@H](C1)[C@@H]1c1ccc(cc(cc3)C(N)=N)c3c1)cc2
CHEMBL185269	422.49	3.02	7	4	112.58	C[C@@H](C(Nc(cc1)ccc1-[n]1c(cccc2)c2nc1)=O)c1c[nH]c(cc2)c1cc2C(N)=N
CHEMBL184844	381.52	5.45	3	2	62.23	CC(C)C1=NCCc2c1ccc([C@H](C1)[C@@H]1c(ccc1c3)cc1ccc3C(N)=N)c2
CHEMBL184223	387.44	3.64	6	3	79.46	Cc1cccc(NC(Nc(cc2)ccc2-c(cc2)c(CNC3=O)c3c2OC)=O)c1
CHEMBL183021	381.52	5.45	3	2	62.23	CC(C)C1=NCCc2c1cc([C@@H](C1)[C@@H]1c(ccc1c3)cc1ccc3C(N)=N)cc2
CHEMBL182547	437.63	6.29	3	2	53.11	CN(CC1)[C@@H](C2CCCCC2)c2c1ccc([C@@H](C1)[C@H]1c1ccc(cc(cc3)C(N)=N)c3c1)c2
CHEMBL182460	415.54	6.12	3	2	62.23	NC(c1ccc(cc([C@@H](C2)[C@H]2c2cc(C(c3ccccc3)=NCC3)c3cc2)cc2)c2c1)=N
CHEMBL182303	421.59	6.12	3	2	62.23	NC(c1ccc(cc([C@H](C2)[C@@H]2c2cc(C(C3CCCCC3)=NCC3)c3cc2)cc2)c2c1)=N
CHEMBL182065	381.52	5.45	3	2	62.23	CC(C)C1=NCCc2c1cc([C@H](C1)[C@@H]1c(ccc1c3)cc1ccc3C(N)=N)cc2
CHEMBL182061	444.53	5.21	5	2	105.18	C[C@H](c(cc1)ccc1F)/N=C(/Nc(cc1)ccc1NC(c1nc2ccccc2cc1)=O)\S
CHEMBL182024	444.53	5.08	5	2	105.18	C[C@H](c(cc1)ccc1F)/N=C(\Nc(cc1)ccc1NC(c1cc2ccccc2cn1)=O)/S
CHEMBL181659	436.49	4.77	5	2	116.08	C[C@@H](c(cc1)ccc1F)/N=C(/Nc(cc1)cc(C#N)c1NC(c(cccc1)c1F)=O)\S
CHEMBL181658	420.42	3.98	6	3	94.02	C[C@H](c(cc1)ccc1F)NC(Nc(cc1)cc(C#N)c1NC(c(cccc1)c1F)=O)=O
CHEMBL180991	444.77	3.48	6	3	131.20	Nc1ccc(/C=C\C(N[C@H](C(Cl)(Cl)Cl)/N=C(\Nc2ncccc2)/S)=O)cc1
CHEMBL180649	403.72	3.44	5	3	98.14	O=C(c1ccccc1)N[C@H](C(Cl)(Cl)Cl)NC(Nc1ncccc1)=S
CHEMBL180508	381.71	3.65	5	2	105.18	CC(C)=CC(N[C@H](C(Cl)(Cl)Cl)/N=C(\Nc1ncccc1)/S)=O
CHEMBL180317	382.22	3.39	5	3	118.63	OC(/C(\c1c[s]cc1)=C\c1c(C(O)=O)[nH]c2cc(Cl)cc(Cl)c12)=O
CHEMBL180067	366.16	2.74	6	3	103.53	OC(/C(\c1c[o]cc1)=C\c1c(C(O)=O)[nH]c2cc(Cl)cc(Cl)c12)=O
CHEMBL179941	381.52	5.45	3	2	62.23	CC(C)C1=NCCc2c1cc([C@H](C1)[C@@H]1c(ccc1c3)cc1ccc3C(N)=N)cc2
CHEMBL179865	377.18	2.60	6	3	103.28	OC(/C(\c1cnccc1)=C\c1c(C(O)=O)[nH]c2cc(Cl)cc(Cl)c12)=O
CHEMBL179560	419.26	3.50	6	3	93.63	CN(C)c1cccc(/C(\C(O)=O)=C/c2c(C(O)=O)[nH]c3cc(Cl)cc(Cl)c23)c1
CHEMBL177880	388.37	4.14	6	4	115.06	OC(c1cc2ccccc2c(Cc(c2ccccc2cc2C(O)=O)c2O)c1O)=O
CHEMBL176822	449.53	2.08	8	4	136.76	NC(c1cccc(NC(Nc(cc2)ccc2S(N(CC2)Cc3c2cccc3)(=O)=O)=O)c1)=N
CHEMBL176772	441.42	4.61	3	2	31.06	FC(c1cc(CN2C[C@H](Cc3c[nH]c4c3cccc4)NCC2)cc(C(F)(F)F)c1)(F)F

CHEMBL176252	408.92	5.52	3	2	57.53	OC(/C=C\c(cc1)cc(Cl)c1-c(cc1)cc(C2(C[C@H](C3)C4)C[C@H]4C[C@H]3C2)c1O)=O
CHEMBL175874	406.36	4.59	4	2	44.37	Cc1ccc(C2(CNC(Nc3cc(Cl)cc(Cl)c3)=O)CCN(C)CC2)cc1
CHEMBL175860	455.39	4.65	5	3	66.05	O=C(NCC1(CCNCC1)c(cc1)ccc1-c1cnccc1)Nc1cc(Cl)cc(Cl)c1
CHEMBL175356	402.50	3.99	6	3	82.80	CC(C)(C)OC(N[C@H](Cc1c[nH]c2c1cccc2)c1nc(-c2ccccc2)c[nH]1)=O
CHEMBL175211	435.53	5.46	5	2	65.20	O=C(c(cccc1)c1-c1ccccc1)Nc(cc1)ccc1C(N(CCCC1)c2c1[nH]cc2)=O
CHEMBL173802	428.28	4.70	5	4	89.79	Oc(ccc(Br)c1)c1C(Nc1ccc(CCc(cc2)cc(O)c2O)cc1)=O
CHEMBL173746	370.41	4.24	6	3	86.88	Cc1cccc(NC(Nc2nc(cc(cc3)C(c4ccccc4)=O)c3[nH]2)=O)c1
CHEMBL173503	440.46	6.38	4	2	58.20	CC(C)(C)c(cc1)ccc1C(Nc(cccc1)c1C(Nc1ccc(C(F)(F)F)cc1)=O)=O
CHEMBL173051	386.57	5.23	3	3	60.69	C[C@@H](CC#CC(C)(C)O)[C@@H](CC1)C(C)(C)[C@H]1/C=C\C=C(\C[C@H](C[C@H]1O)O)/C1=C
CHEMBL172771	428.61	4.67	4	4	80.92	C[C@H](CC#C[C@@](C)(CO)O)[C@@H](CC1)[C@@](C)(CCC2)[C@@H]1/C\2=C\C=C(\C[C@@H](C[C@@H]1O)O)/C1=C
CHEMBL172336	420.54	4.09	6	4	136.31	CC(C)(C)c(cc1)ccc1C(Nc1c(C(Nc2cc(C(N)=N)ccc2)=O)[s]cc1)=O
CHEMBL172060	427.71	6.54	2	2	32.26	CC(C)[C@H](C)/C=C\[C@H](C)[C@H]1[C@](C)(CC[C@@H]2C3=CC[C@@H](C4)[C@@]2(C)CC[C@H]4O)[C@@]3(C)NCC1
CHEMBL170353	390.46	5.64	4	2	58.20	CC(C)(C)c(cc1)ccc1C(Nc(cccc1)c1C(Nc(cc1)ccc1F)=O)=O
CHEMBL169358	372.47	5.54	4	2	58.20	CC(C)(C)c(cc1)ccc1C(Nc(cccc1)c1NC(c1ccccc1)=O)=O
CHEMBL166477	435.31	4.92	6	3	95.50	CC(C)(C)C(N/C(\C(O)=O)=C\c(cc1)ccc1NC(c(c(Cl)ccc1)c1Cl)=O)=O
CHEMBL161876	363.17	4.43	3	2	45.11	N=C(c(cc1)cc(Cl)c1Cl)NCc(cccc1)c1OC(F)(F)F
CHEMBL161180	363.17	4.43	3	2	45.11	N=C(c(cc1)ccc1OC(F)(F)F)NCc1cc(Cl)cc(Cl)c1
CHEMBL160595	361.83	4.60	4	2	57.78	O=C(Cc1cccc2ccccc12)Nc1cc(-c(cc2)ccc2Cl)n[nH]1
CHEMBL158732	361.83	4.60	4	2	57.78	O=C(Cc1cc2ccccc2cc1)Nc1cc(-c(cc2)ccc2Cl)n[nH]1
CHEMBL157164	397.90	5.27	4	2	66.40	CC(C)(C)c(cc1)ccc1-c(ccc(NC(C(CCC1)=C1C(O)=O)=O)c1)c1Cl
CHEMBL155875	362.60	5.23	2	2	40.46	C[C@H](CCCO)[C@H](CC1)[C@](C)(CC2)[C@H]1[C@@H](CC1)[C@H]2[C@@](C)(CC2)[C@H]1C[C@H]2O
CHEMBL154908	420.69	4.42	4	2	66.40	OC(C(CCC1)=C1C(Nc(ccc(-c(cc1)ccc1Br)c1)c1Cl)=O)=O
CHEMBL153660	386.50	3.08	5	3	105.32	C/N=C(\c1ccc(/C=C(/CCCC/C2=C\c(cc3)ccc3C(N)=N)\C/2=O)cc1)/N
CHEMBL153659	400.52	3.17	5	3	94.03	CN(C)C(c1ccc(/C=C(/CCCC/C2=C/c(cc3)ccc3C(N)=N)\C/2=O)cc1)=N
CHEMBL151562	386.50	3.08	5	3	105.32	C/N=C(/c1ccc(/C=C(/CCCC/C2=C\c(cc3)ccc3C(N)=N)\C/2=O)cc1)\N
CHEMBL151481	400.52	3.17	5	3	94.03	CN(C)C(c1ccc(/C=C(\CCCC/C2=C/c(cc3)ccc3C(N)=N)/C/2=O)cc1)=N
CHEMBL151480	373.46	2.96	5	3	110.03	NC(c1ccc(/C=C(\CCCC/C2=C\c(cc3)ccc3C(N)=O)/C/2=O)cc1)=N
CHEMBL151294	378.26	1.00	6	4	125.77	NC(c(cc1)ccc1-c1cnc(-c(cc2)ccc2C(N)=N)[o]1)=N.Cl.Cl
CHEMBL150547	373.46	2.96	5	3	110.03	NC(c1ccc(/C=C(\CCCC/C2=C/c(cc3)ccc3C(N)=O)/C/2=O)cc1)=N
CHEMBL149590	372.46	3.36	6	4	128.62	N=C(NCc1ccccc1)Nc1nc(-c2c[nH]c(cc3)c2cc3C#N)c[s]1
CHEMBL148720	424.37	4.06	5	3	70.23	CC(C)(C1)NC(C)(C)CC1NC(/C=C\C=C\C(Nc(cc1)cc(Cl)c1Cl)=O)=O
CHEMBL148499	380.50	6.51	6	2	179.08	N/C(\S)=N\N=C/c1c(cccc2)c2c(/C=N\N=C(/N)\S)c2ccccc12
CHEMBL147068	376.33	4.46	5	3	70.59	Oc1ccc(cccc2NC(NCc(cc3)ccc3OC(F)(F)F)=O)c2c1

CHEMBL146646	374.48	5.14	3	2	57.53	OC(c1ccc(/C=C\c(cc2)cc(C3(C[C@H](C4)C5)C[C@H]5C[C@H]4C3)c2O)cc1)=O
CHEMBL145911	426.51	5.61	4	2	74.60	OC(c1ccc(cc(cc2)-c(cc3)cc(C4(C[C@H](C5)C6)C[C@H]6C[C@H]5C4)c3C(O)=O)c2c1)=O
CHEMBL143748	476.97	3.24	8	4	102.15	O=C(Nc1cc(CN[C@H](C2)Cc3c2ccc(Cl)c3)n[nH]1)Nc1c([C@@H](CCC2)N2C2=O)c2ccc1
CHEMBL143610	438.45	5.29	4	2	58.20	CC(N[C@H](C1)Cc2c1ccc(NC(c(cccc1)c1-c1ccc(C(F)(F)F)cc1)=O)c2)=O
CHEMBL143160	359.47	5.41	3	2	49.33	CC(C)(C)c1cc(-c(cc2)ccc2C(NCc2ccccc2)=O)cc(O)c1
CHEMBL142335	359.47	5.41	3	2	49.33	CC(C)(C)c(cc1)cc(O)c1-c(cc1)ccc1C(NCc1ccccc1)=O
CHEMBL141365	412.47	3.63	5	3	69.28	O=C(c1cc(cccc2)c2[nH]1)NC[C@@H](CNc1c2cccc1)N=C2c(cccc1)c1F
CHEMBL137041	359.47	4.21	4	2	44.37	O=C(NC1CCN(Cc2ccccc2)CC1)Nc1cccc2ccccc12
CHEMBL133250	405.32	3.78	5	2	101.10	COc(cc1)cc2c1[nH]cc2CC/N=C(\Nc(cc1)ncc1Br)/S
CHEMBL131347	376.91	3.49	5	2	105.18	CC(C)(C)[C@H](NC(c(cc1)ccc1Cl)=O)N/C(\S)=N/c1cnccc1
CHEMBL127355	433.39	2.98	7	4	133.34	NC(c(cc1)cc(C(F)(F)F)c1C(Nc(cc1)cc(CC[C@H]2CC(O)=O)c1C2=O)=O)=N
CHEMBL126248	446.29	2.12	7	4	121.52	NC(/C=C\c(cc1)ccc1NC(/C=C(\C[C@H](C(O)=O)Nc1cc(Cl)c2)/c1c2Cl)=O)=O
CHEMBL126174	454.57	4.51	6	3	88.39	N#Cc1cccc(NC(Nc2c(CN3CCC(Cc4ccccc4)CC3)ccc(CO)c2)=O)c1
CHEMBL125925	451.57	2.68	7	3	98.74	OC(CC[C@H](C(NN1CCC2(CCCCC2)CC1)=O)NC(c1cc2ccccc2cc1)=O)=O
CHEMBL125884	472.56	4.61	6	3	88.39	N#Cc1cccc(NC(Nc2c(CN3CCC(Cc(cc4)ccc4F)CC3)cc(CO)cc2)=O)c1
CHEMBL125132	454.57	4.46	6	3	88.39	N#Cc1cccc(NC(Nc2c([C@H](CN3CCC(Cc4ccccc4)CC3)O)cccc2)=O)c1
CHEMBL124473	485.56	4.29	7	3	111.25	NC(c(cc1)cc(CN2CCC(Cc(cc3)ccc3F)CC2)c1NC(Nc1cc(C#N)ccc1)=O)=O
CHEMBL124311	448.31	2.12	7	4	107.53	CC(NCc(cc1)ccc1NC(/C=C(\C[C@H](C(O)=O)Nc1cc(Cl)c2)/c1c2Cl)=O)=O
CHEMBL12294	417.62	2.69	4	2	76.04	C[C@@H](Cc1c[nH]cn1)N.S=C(NC1CCCCC1)N(CC1)CCC1c1c[nH]cn1
CHEMBL122023	444.92	2.86	8	3	99.77	CCC[C@@H](C(Nc(cc1)ccc1N(CCOC1)C1=O)=O)NC(Nc(cc1)ccc1Cl)=O
CHEMBL1214854	429.27	2.37	6	3	99.26	OC(CCc1c(/C=C(/c(cc(cc2)Br)c2N2)\C2=O)[nH]c(CCC2)c1C2=O)=O
CHEMBL120559	384.24	5.30	6	3	133.08	OC(c(cc1)ccc1N/C(\S)=N\N=C/c1cc(Cl)cc(Cl)c1O)=O
CHEMBL1203501	375.93	3.55	5	3	107.33	Cc1cc(-c2c(C)[s]c(N/C(\N)=N\Cc3ccc(C)cc3)n2)c[nH]1.Cl
CHEMBL1203483	413.93	3.46	6	4	114.06	COc(cc1)cc2c1[nH]cc2-c1c[s]c(NC(NCc2ccccc2)=N)n1.Cl
CHEMBL1202285	403.35	3.83	4	3	89.04	Cc1cccc(-c2c[s]c(NC(NCc3ccccc3)=N)n2)c1.Br
CHEMBL1202284	403.35	3.83	4	3	89.04	Cc(cc1)ccc1-c1c[s]c(NC(NCc2ccccc2)=N)n1.Br
CHEMBL1202277	389.32	3.49	4	3	89.04	N=C(NCc1ccccc1)Nc1nc(-c2ccccc2)c[s]1.Br
CHEMBL1202262	423.77	4.09	4	3	89.04	N=C(NCc1ccccc1)Nc1nc(-c(cccc2)c2Cl)c[s]1.Br
CHEMBL1202258	403.35	3.83	4	3	89.04	Cc(cccc1)c1-c1c[s]c(NC(NCc2ccccc2)=N)n1.Br
CHEMBL116592	388.51	5.26	3	2	57.53	Cc(cc(/C=C\C(O)=O)cc1)c1-c(cc1)cc(C2(C[C@H](C3)C4)C[C@H]4C[C@H]3C2)c1O
CHEMBL1163677	392.33	3.40	4	3	65.28	NCCCN(CCc1c2[nH]c(cc3)c1cc3Cl)[C@@H]2c1cc(O)ccc1.Cl
CHEMBL1163629	385.94	3.59	4	3	65.28	Cc(cc1)cc2c1[nH]c1c2CCN(CCCCN)[C@@H]1c1cccc(O)c1.Cl
CHEMBL1163628	371.91	3.13	4	3	65.28	Cc(cc1)cc2c1[nH]c1c2CCN(CCCN)[C@H]1c1cccc(O)c1.Cl

CHEMBL114721	388.37	4.14	6	4	115.06	OC(c(cc(Cc(c1ccccc11)cc(C(O)=O)c1O)c1ccccc11)c1O)=O
CHEMBL114571	428.58	4.62	5	2	51.37	Cc(cc1)ccc1NC(N(CC1)CC=C1c(cc1)cc2c1[nH]cc2C[C@H]1N(C)CCC1)=O
CHEMBL114420	448.54	3.19	6	3	112.96	OC(c(cc1)cc([C@H]2SCCNC(NCc3ccccc3)=O)c1OCc1c2cccc1)=O
CHEMBL114324	464.61	3.76	5	2	135.02	OC(c(cc1)cc([C@H]2SCC/N=C(/NCc3ccccc3)\S)c1OCc1c2cccc1)=O
CHEMBL114043	446.05	3.20	6	4	115.06	OC(c1cc(Cc(cc2C(O)=O)cc(Br)c2O)cc(Br)c1O)=O
CHEMBL113868	436.67	4.91	4	1	73.43	CN1[C@H](Cc2c[nH]c(cc3)c2cc3C(CC2)=CCN2/C(\S)=N\C2CCCCC2)CCC1
CHEMBL112123	380.49	5.39	5	3	78.43	Cc(ccc(NC(C1CCCCCCC1)=O)c1)c1NC(c(cc1)ccc1O)=O
CHEMBL110027	381.43	4.09	5	3	88.20	NC(c1ccc(cc(cc2)C(Nc3cc(Oc4ccccc4)ccc3)=O)c2c1)=N
CHEMBL109367	384.48	3.74	5	3	91.33	CC(C)C1=NCCc(cc2)c1cc2NC(c(ccc1c2)cc1ccc2C(N)=N)=O
CHEMBL1086957	356.25	4.11	4	2	62.32	CC[C@@H](C)[C@@]1(CC(O)=O)OCCc(c2c(cc3)Cl)c1[nH]c2c3Cl
CHEMBL107883	482.62	3.99	8	3	103.53	CC(C)C[C@@H](c1c(C[C@H]2C(N[C@H]3CCCCNC(OC(C)(C)C)=O)=O)c(cccc4)c4[nH]1)N2C3=O
CHEMBL1077282	347.34	4.34	4	2	57.78	CCCC(Nc1n[nH]c2c1ccc(-c1ccc(C(F)(F)F)cc1)c2)=O
CHEMBL107427	470.57	2.83	7	3	104.53	NCc(cc1)ccc1NC(CN(CCCC[C@H]1NC(c(cc2)ccc2-c2ccccc2)=O)C1=O)=O
CHEMBL105778	399.45	4.14	6	3	90.79	CC(Nc(cc1)cc(Nc2c3cccc2)c1/C\3=N/c1ccc(CCC(O)=O)cc1)=O
CHEMBL105395	359.43	3.32	5	3	88.20	NC(c1ccc(cc(cc2[C@@H]3COCC3)C(Nc3ccccc3)=O)c2c1)=N
CHEMBL104434	470.57	2.83	7	3	104.53	NCc(cccc1)c1NC(CN(CCCC[C@@H]1NC(c(cc2)ccc2-c2ccccc2)=O)C1=O)=O
CHEMBL104220	484.60	3.18	7	3	104.53	Cc(cc1)ccc1-c(cc1)ccc1C(N[C@H](CCCCN1CC(Nc2cc(CN)ccc2)=O)C1=O)=O
CHEMBL104064	484.60	3.18	7	3	104.53	Cc(ccc(CN)c1)c1NC(CN(CCCC[C@H]1NC(c(cc2)ccc2-c2ccccc2)=O)C1=O)=O
CHEMBL103284	433.62	5.23	3	2	53.11	CC[C@@H](C)c(cc1)ccc1N(Cc1ccc(C(C)(C)C)cc1)C(N)=N.CS(O)(=O)=O
CHEMBL101101	389.84	4.24	5	2	105.43	O=C(c1ccc[o]1)Nc(cc1)ccc1/N=C(\Nc(cc1)cc(Cl)c1F)/S
CHEMBL100393	437.71	5.98	2	2	32.26	C[C@H]([C@H](CC1)[C@H](CC2)[C@@]1(C)[C@@H](CC1)[C@@H]2[C@@](C)(CC2)[C@@H]1C(C)(C)[C@H]2O)NCc1ccccc1
MI-89*	414.58	4.95	4	1	71.44	C[C@H](CCC(O)=O)[C@@H](CC1)[C@@](C)(CC[C@@H]2[C@@](C)(CCC(C3)=O)[C@@H]3/C3=C\C)[C@@H]1[C@@H]2C/3=O
MI-73*	460.70	5.96	4	3	77.76	C[C@H](CCC(O)=O)[C@@H](CC1)[C@@](C)(CC[C@@H]2[C@@](C)(CC[C@H](C3)O)[C@@H]3C3)[C@@H]1[C@@H]2[C@]3(CCCC=C)O
MI-196*	518.82	7.96	4	3	77.76	CCCCCCCCC[C@@]1([C@@H]([C@H](CC2)[C@](C)(CC3)[C@H]2[C@H](C)CCC(O)=O)[C@H]3[C@@](C)(CC[C@H](C2)O)[C@@H]2C1)O
MI-195*	460.70	5.96	4	3	77.76	C[C@H](CCC(O)=O)[C@@H](CC1)[C@@](C)(CC[C@@H]2[C@@](C)(CC[C@H](C3)O)[C@@H]3C3)[C@@H]1[C@@H]2[C@]3(CCCC=C)O
MI-168*	416.60	3.95	4	3	77.76	C[C@H](CCC(O)=O)[C@@H](CC1)[C@@](C)(CC[C@@H]2[C@@](C)(CC[C@H](C3)O)[C@@H]3C3)[C@@H]1[C@@H]2[C@]3(C#C)O
MI-147*	418.62	4.66	4	3	77.76	C[C@H](CCC(O)=O)[C@@H](CC1)[C@@](C)(CC[C@@H]2[C@@](C)(CC[C@H](C3)O)[C@@H]3/C3=C\C)[C@@H]1[C@@H]2[C@H]/3O
MI-145*	420.63	4.76	4	3	77.76	CC[C@H]([C@H](C[C@@H](CC1)O)[C@@]1(C)[C@@H](CC1)[C@@H]2[C@H](CC3)[C@@]1(C)[C@H]3[C@H](C)CCC(O)=O)[C@@H]2O

* Synthesized compound from our laboratory without PubChem CID; molecular weight (MW); partition coefficient (logP); hydrogen bond acceptor (HBA); hydrogen bond donor (HBD); polar surface area (PSA)

Table S3. Pharmacophore models generated by the Pharmit server

Model	Feature								TP	TN	FP	FN	Sensitivity	Specificity	ROC Score	D	A	Ht	Ha	%YA	%RA	E	GH
	Acc	Don	Acc	Don	Hyd	Hyd	Hyd	Hyd															
Model-1	C3	C3	-	-	C6	C10	C13	C20	2	436	1	13	0.13	1.00	0.57	452	15	3	2	0.67	0.13	20.09	0.53
Model-2	C3	C3	-	-	R1	C10	C13	C20	13	430	7	2	0.87	0.98	0.93	452	15	20	13	0.65	0.87	19.59	0.72
Model-3	C3	C3	-	-	R2	C10	C13	C20	13	430	7	2	0.87	0.98	0.93	452	15	20	13	0.65	0.87	19.59	0.72
Model-4*	C3	C3	-	-	R3	C10	C13	C20	13	431	6	2	0.87	0.99	0.93	452	15	19	13	0.68	0.87	20.62	0.74
Model-5*	C3	C3	-	-	R4	C10	C13	C20	13	431	6	2	0.87	0.99	0.93	452	15	19	13	0.68	0.87	20.62	0.74
Model-6	C3	C3	C7	C7	R1	C20	-	-	9	430	7	6	0.60	0.98	0.79	452	15	16	9	0.56	0.60	16.95	0.58
Model-7	C3	C3	C7	C7	C10	C20	-	-	8	433	4	7	0.53	0.99	0.76	452	15	12	8	0.67	0.53	20.09	0.64
Model-8	C3	C3	C7	C7	C13	C20	-	-	9	433	4	6	0.60	0.99	0.80	452	15	13	9	0.69	0.60	20.86	0.68
Model-9	C3	C3	C7	C7	R4	C20	-	-	8	431	6	7	0.53	0.99	0.76	452	15	14	8	0.57	0.53	17.22	0.57
Model-10	C3	C3	C7	C7	R2	C20	-	-	8	431	6	7	0.53	0.99	0.76	452	15	14	8	0.57	0.53	17.22	0.57
Model-11	C3	C3	C7	C7	R3	C20	-	-	9	432	5	6	0.60	0.99	0.79	452	15	14	9	0.64	0.60	19.37	0.64
Model-12	C3	C3	C7	C7	C6	C20	-	-	2	435	2	13	0.13	1.00	0.56	452	15	4	2	0.50	0.13	15.07	0.41
Model-13	C3	C3	C7	C7	C10	C13	-	-	9	432	5	6	0.60	0.99	0.79	452	15	14	9	0.64	0.60	19.37	0.64
Model-14	C3	C3	C7	C7	R1	C10	-	-	11	420	17	4	0.73	0.96	0.85	452	15	28	11	0.39	0.73	11.84	0.50
Model-15	C3	C3	C7	C7	R2	C10	-	-	11	424	13	4	0.73	0.97	0.85	452	15	24	11	0.46	0.73	13.81	0.54
Model-16	C3	C3	C7	C7	R3	C10	-	-	9	427	10	6	0.60	0.98	0.79	452	15	19	9	0.47	0.60	14.27	0.52
Model-17	C3	C3	C7	C7	R4	C10	-	-	8	430	7	7	0.53	0.98	0.76	452	15	15	8	0.53	0.53	16.07	0.54
Model-18	C3	C3	C7	C7	R1	C13	-	-	9	432	5	6	0.60	0.99	0.79	452	15	14	9	0.64	0.60	19.37	0.64
Model-19	C3	C3	C7	C7	R2	C13	-	-	9	430	7	6	0.60	0.98	0.79	452	15	16	9	0.56	0.60	16.95	0.58
Model-20	C3	C3	C7	C7	R3	C13	-	-	9	430	7	6	0.60	0.98	0.79	452	15	16	9	0.56	0.60	16.95	0.58
Model-21	C3	C3	C7	C7	R4	C13	-	-	8	432	5	7	0.53	0.99	0.76	452	15	13	8	0.62	0.53	18.54	0.60
Model-22	C3	C3	-	-	R1	R2	C10	C13	15	428	9	0	1.00	0.98	0.99	452	15	24	15	0.63	1.00	18.83	0.73
Model-23	C3	C3	-	-	R1	R3	C10	C13	15	426	11	0	1.00	0.97	0.99	452	15	26	15	0.58	1.00	17.38	0.70
Model-24*	C3	C3	-	-	R1	R4	C10	C13	15	430	7	0	1.00	0.98	0.99	452	15	22	15	0.68	1.00	20.55	0.77
Model-25	C3	C3	-	-	R1	R2	C10	C20	13	426	11	2	0.87	0.97	0.92	452	15	24	13	0.54	0.87	16.32	0.64
Model-26	C3	C3	-	-	R1	R3	C10	C20	13	428	9	2	0.87	0.98	0.92	452	15	22	13	0.59	0.87	17.81	0.67
Model-27*	C3	C3	-	-	R1	R4	C10	C20	13	431	6	2	0.87	0.99	0.93	452	15	19	13	0.68	0.87	20.62	0.74
Model-28*	C3	C3	-	-	R1	R2	C13	C20	15	429	8	0	1.00	0.98	0.99	452	15	23	15	0.65	1.00	19.65	0.75
Model-29*	C3	C3	-	-	R1	R3	C13	C20	15	430	7	0	1.00	0.98	0.99	452	15	22	15	0.68	1.00	20.55	0.77
Model-30*	C3	C3	-	-	R1	R4	C13	C20	14	431	6	1	0.93	0.99	0.96	452	15	20	14	0.70	0.93	21.09	0.77
Model-31*	C3	C3	-	-	R3	R4	C10	C13	15	430	7	0	1.00	0.98	0.99	452	15	22	15	0.68	1.00	20.55	0.77

Model	Feature								TP	TN	FP	FN	Sensitivity	Specificity	ROC Score	D	A	Ht	Ha	%YA	%RA	E	GH
	Acc	Don	Acc	Don	Hyd	Hyd	Hyd	Hyd															
Model-32*	C3	C3	-	-	R3	R4	C10	C20	13	431	6	2	0.87	0.99	0.93	452	15	19	13	0.68	0.87	20.62	0.74
Model-33*	C3	C3	-	-	R3	R4	C13	C20	15	429	8	0	1.00	0.98	0.99	452	15	23	15	0.65	1.00	19.65	0.75
Model-34	C3	C3		C7	R1	R4	C10		8	433	4	7	0.53	0.99	0.76	452	15	12	8	0.67	0.53	20.09	0.64
Model-35	C3	C3		C7	R1	R2	C10		9	432	5	6	0.60	0.99	0.79	452	15	14	9	0.64	0.60	19.37	0.64
Model-36	C3	C3		C7	R1	R3	C10		9	432	5	6	0.60	0.99	0.79	452	15	14	9	0.64	0.60	19.37	0.64
Model-38	C3	C3		C7	R1	R4	C13		8	433	4	7	0.53	0.99	0.76	452	15	12	8	0.67	0.53	20.09	0.64
Model-39	C3	C3		C7	R1	R2	C13		9	433	4	6	0.60	0.99	0.80	452	15	13	9	0.69	0.60	20.86	0.68
Model-40	C3	C3		C7	R1	R3	C13		8	433	4	7	0.53	0.99	0.76	452	15	12	8	0.67	0.53	20.09	0.64
Model-41	C3	C3		C7	R1	C10	C13		9	433	4	6	0.60	0.99	0.80	452	15	13	9	0.69	0.60	20.86	0.68
Model-42	C3	C3		C7	R2	C10	C13		9	433	4	6	0.60	0.99	0.80	452	15	13	9	0.69	0.60	20.86	0.68
Model-43	C3	C3		C7	R3	C10	C13		8	433	4	7	0.53	0.99	0.76	452	15	12	8	0.67	0.53	20.09	0.64
Model-44	C3	C3		C7	R4	C10	C13		8	432	5	7	0.53	0.99	0.76	452	15	13	8	0.62	0.53	18.54	0.60
Model-45	C3	C3		C7	R1	C13	C20		9	433	4	6	0.60	0.99	0.80	452	15	13	9	0.69	0.60	20.86	0.68
Model-46	C3	C3		C7	R2	C13	C20		9	433	4	6	0.60	0.99	0.80	452	15	13	9	0.69	0.60	20.86	0.68
Model-47	C3	C3		C7	R3	C13	C20		8	433	4	7	0.53	0.99	0.76	452	15	12	8	0.67	0.53	20.09	0.64
Model-48	C3	C3		C7	R4	C13	C20		9	433	4	6	0.60	0.99	0.80	452	15	13	9	0.69	0.60	20.86	0.68

* Top-ten pharmacophore model

Acc: hydrogen acceptor; Don: hydrogen donor; Hyd: hydrophobic; TP: true positive; TN: true negative; FN: false negative; FP: false positive; D: Total number of compounds in the test set; A: Number of actives in the test set; Ht: Total number of hits retrieved by the model; Ha: The number of actives retrieved by the model; %YA: The percent yield of actives; %RA: The percent ratio of actives; E: The enrichment factor; GH: The goodness of hits

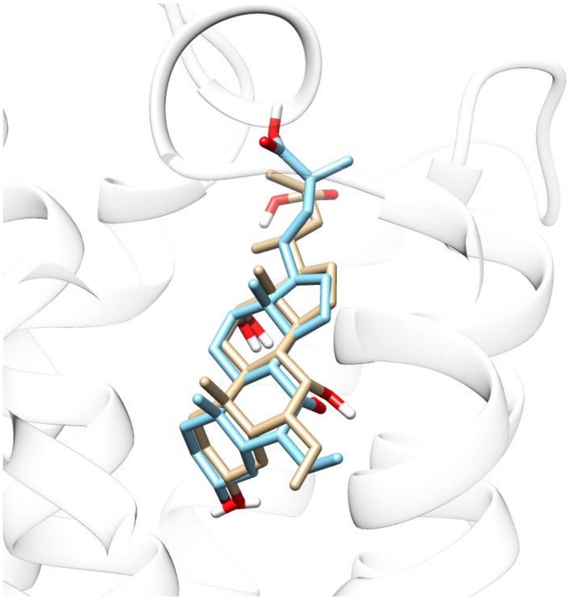

FIGURE S1. Superimposition of re-docked INT-777 (turquoise) and the cocrystallized INT-777 (brown) in the crystal structure of TGR5 (PDB ID: 7CFN)

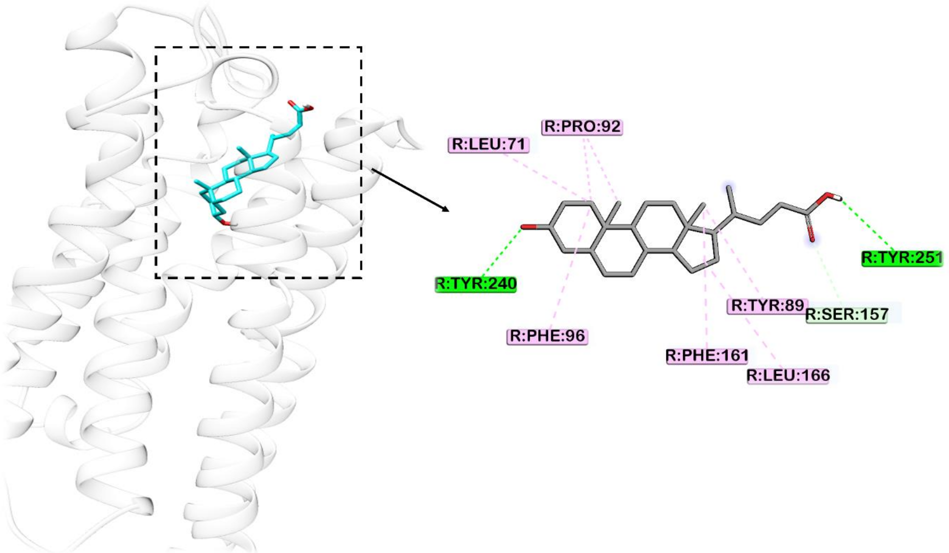

FIGURE S2. Best poses and 2D interaction diagrams of lithocholic acid (LCA) in the ligand binding domain (LBD) of TGR5

Table S4. Detailed information of hit compounds as obtained from the vendor and used for biological testing

ID[a]	Alternative ID[b]	Formula	MW	Purity	Amount (mg)	Smile	Chemical name
CSC057935886 (Hit-1)	CSSS06726389068	C20H24F2N4O	374.4276	100	10.2	CC1CCN(C(C)C1N)C(=O)C2=NN(C=3CCCC23)C=4C=CC=C(F)C4F	1-[1-(2,3-difluorophenyl)-1*H*,4*H*,5*H*,6*H*-cyclopenta[c]pyrazole-3-carbonyl]-2,4-dimethylpiperidin-3-amine
CSC081667704 (Hit-2)	CSSS00081667704	C21H22N4O4	394.4238	100	10.2	CCN1C(=NN=C1C=2C=CC=3OCCOC3C2)N4CCOC=5C=CC(O)=CC5C4	4-[5-(2,3-dihydro-1,4-benzodioxin-6-yl)-4-ethyl-4*H*-1,2,4-triazol-3-yl]-2,3,4,5-tetrahydro-1,4-benzoxazepin-7-ol
CSC089939231 (Hit-3)[c]	CSMS02930605452	C17H23N5O	313.3974	100	10.2	O[C@@]12CCCC[C@H]2CN(CC1)C=3C=CC4=NN=C(C5CC5)N4N3 |&1:1,6,r|	*rac*-(4aR,8aR)-2-{3-cyclopropyl-[1,2,4]triazolo[4,3-*b*]pyridazin-6-yl}-decahydroisoquinolin-4a-ol
CSC083671887 (Hit-4)	CSSS00083671887	C20H17F2N3O3	385.3641	100	10.2	CC1=C(C=NN1C=2C=CC(F)=CC2F)C(=O)N3CCOC=4C=C(O)C=CC4C3	4-[1-(2,4-difluorophenyl)-5-methyl-1*H*-pyrazole-4-carbonyl]-2,3,4,5-tetrahydro-1,4-benzoxazepin-8-ol
CSC085298700 (Hit-5)	CSSS00085298700	C17H18ClF3N2O	358.7858	93	10.2	CC=1C=CC(Cl)=C2C=CC(=NC12)N3CCCC(O)(CC3)C(F)(F)F	1-(5-chloro-8-methylquinolin-2-yl)-4-(trifluoromethyl)azepan-4-ol

[a] The original identifier from the internal database contained in the Pharmit service.

[b] Actual active ID used for the order from the vendor.

[c] Hit-3 was supplied as a non-specified mixture of optical isomers; pure stereoisomer from the virtual screening results (4aR,8aR) was unavailable.

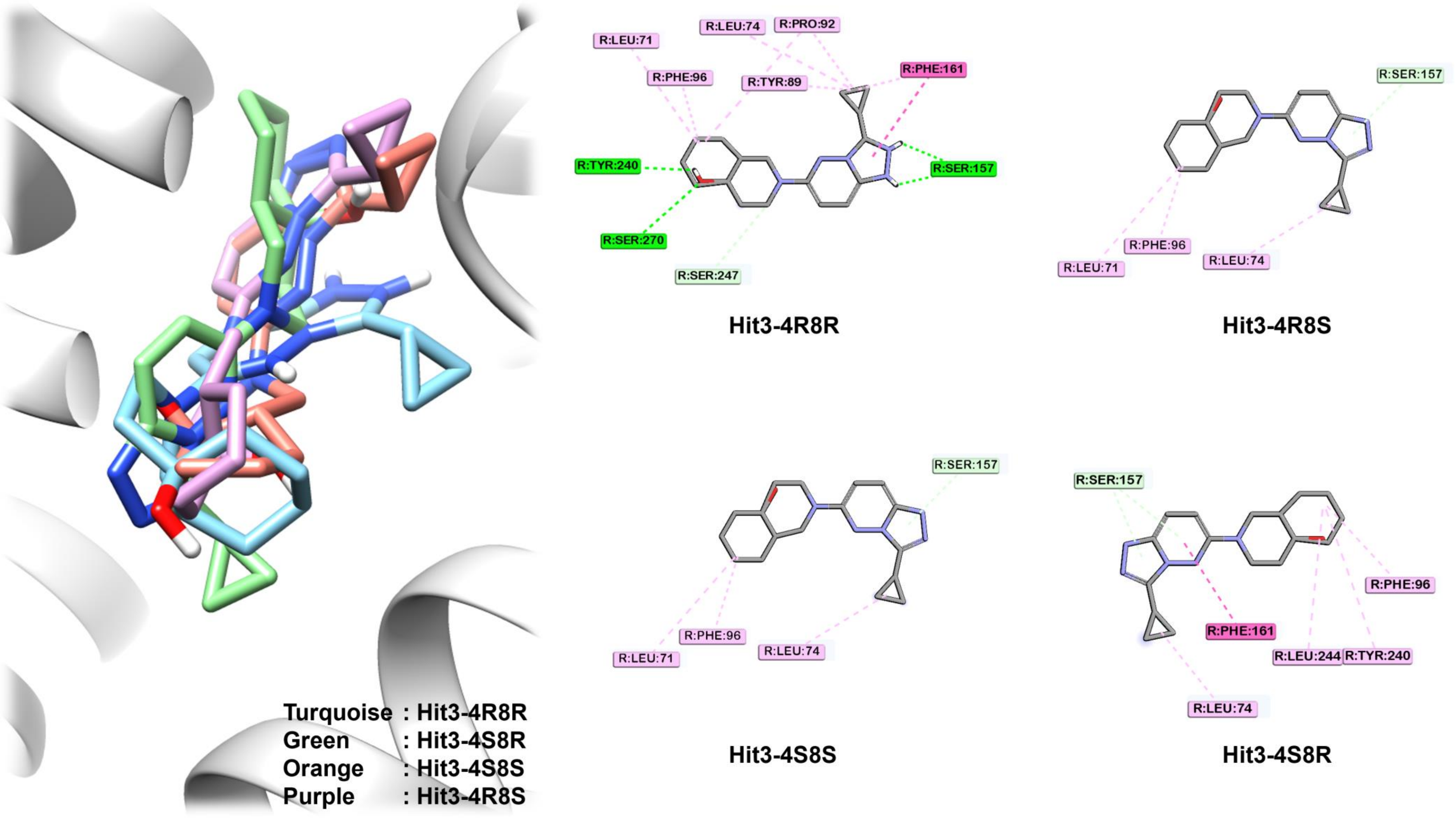

FIGURE S3. Best poses and 2D interaction diagrams of Hit-3 stereoisomers in the ligand binding domain (LBD) of TGR5

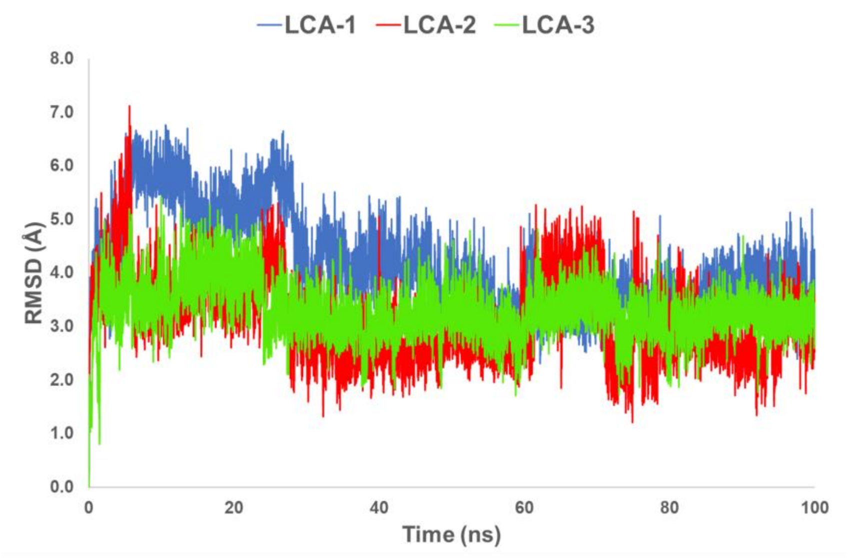

FIGURE S4. RMSD graph of the heavy atoms of lithocholic acid (LCA) as the reference compound (note: 1,2,3 denote replicas)

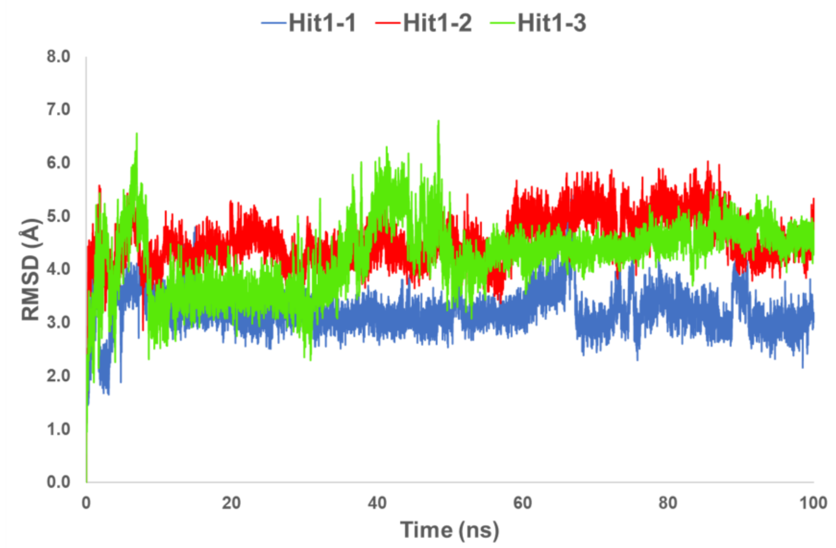

FIGURE S5. RMSD graph of the heavy atoms of Hit-1 (note: 1, 2, 3 denote replicas)

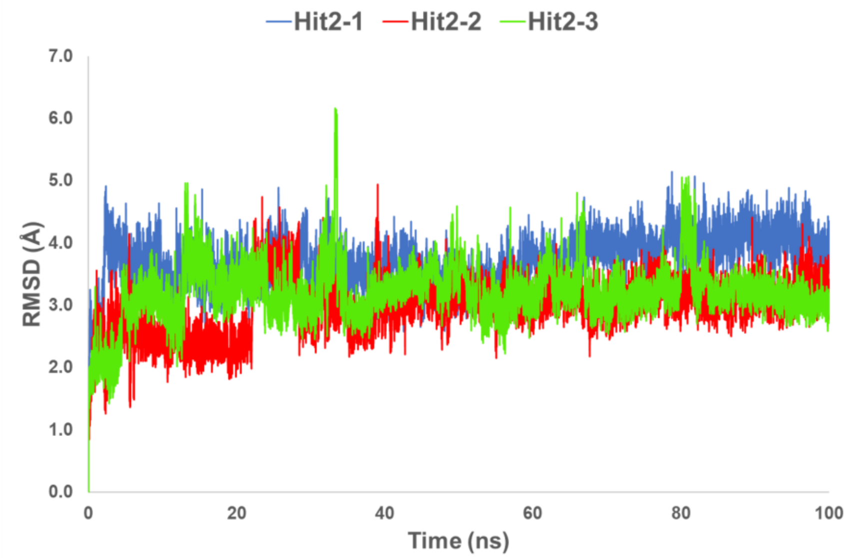

FIGURE S6. RMSD graph of the heavy atoms of Hit-2 (note: 1, 2, 3 denote replicas)

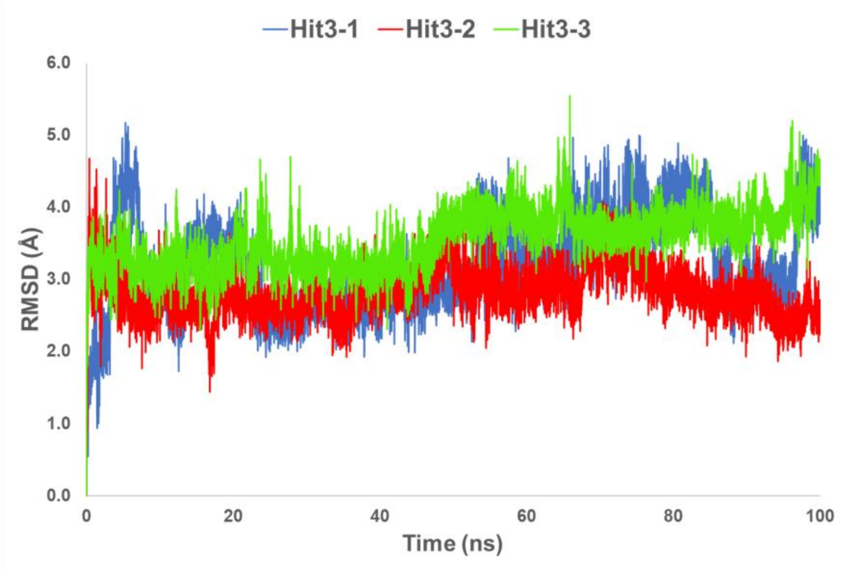

FIGURE S7. RMSD graph of the heavy atoms of Hit-3 (note: 1, 2, 3 denote replicas)

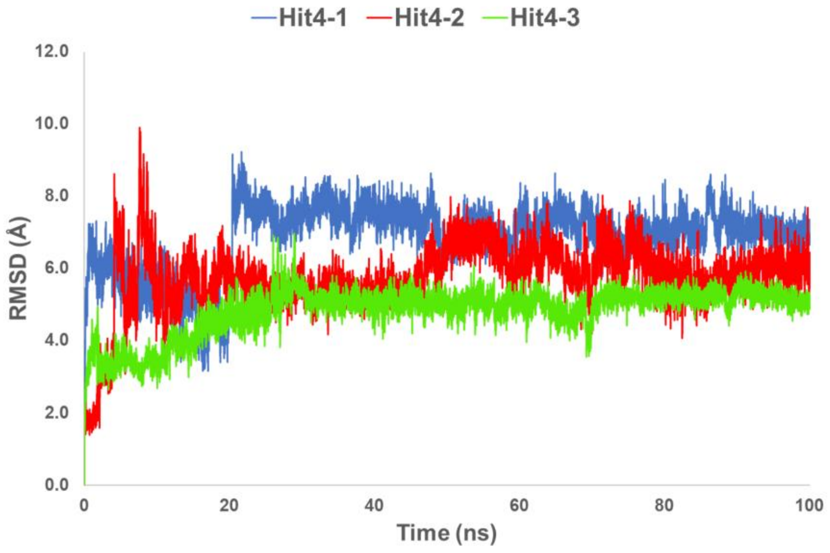

FIGURE S8. RMSD graph of the heavy atoms of Hit-4 (note: 1, 2, 3 denote replicas)

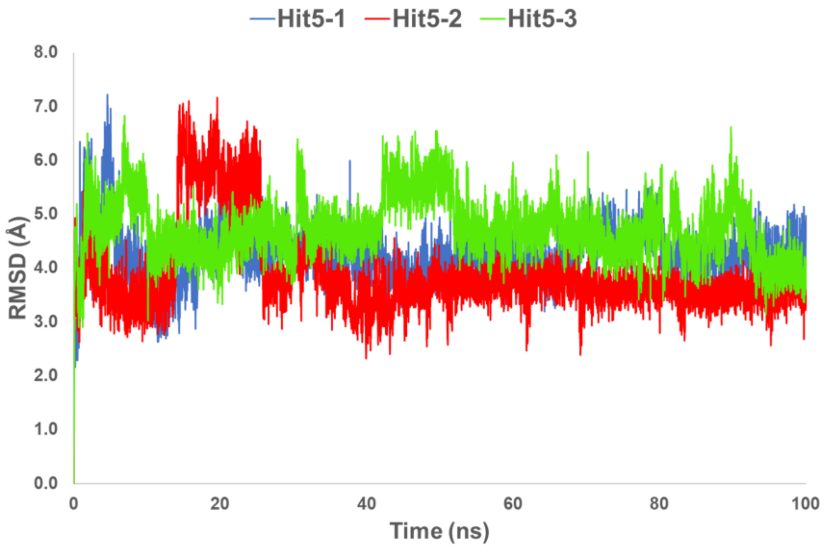

FIGURE S9. RMSD graph of the heavy atoms of Hit-5 (note: 1, 2, 3 denote replicas)

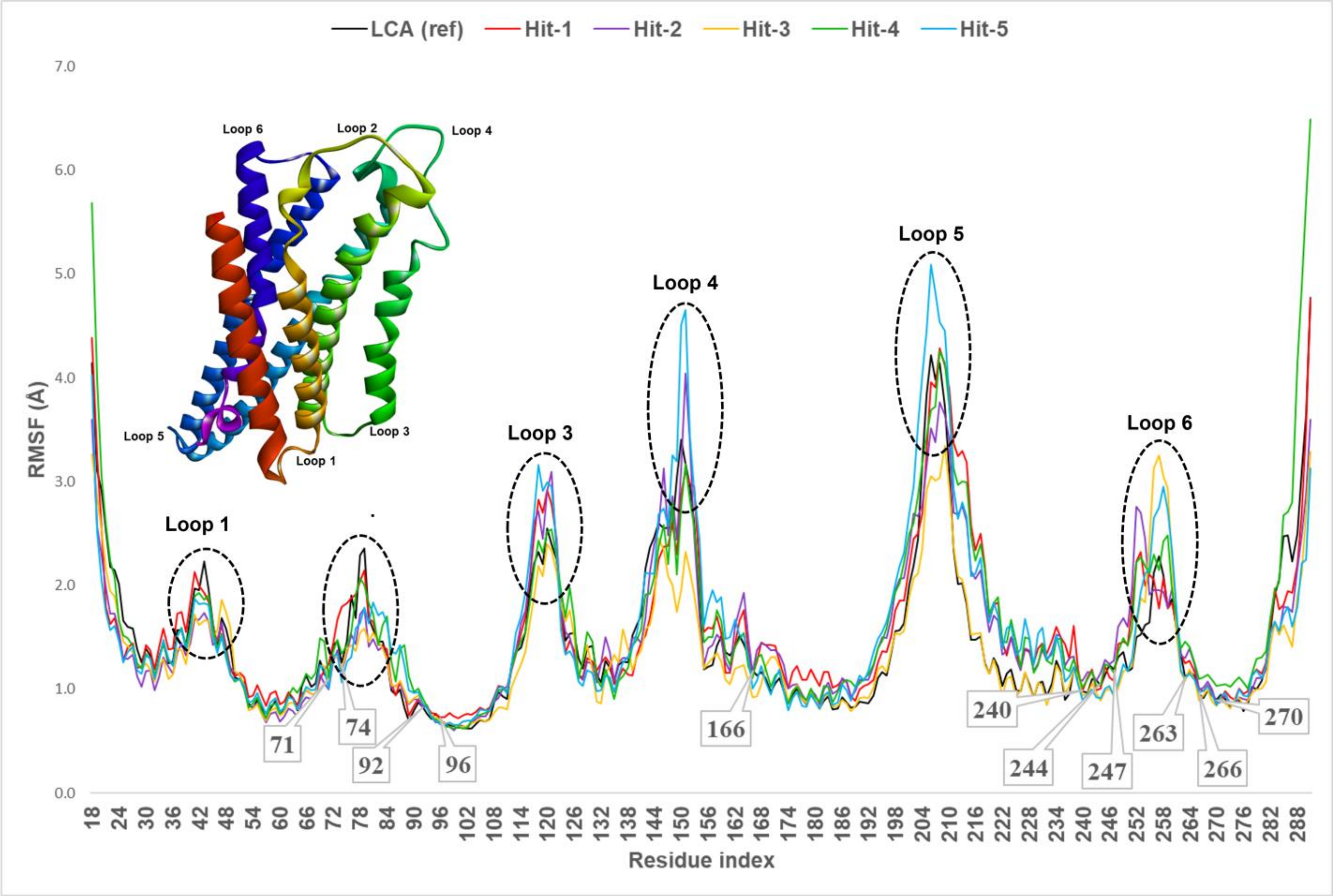

FIGURE S10. RMSF (average from 3 replicas) of Cα of TGR5 complexed with Reference ligand (LCA) and Hit compounds. Simulation time = 100 ns, (numbers in boxes refer to the residues in the binding cavity; the dashed black circle refer to the loop parts of the protein).

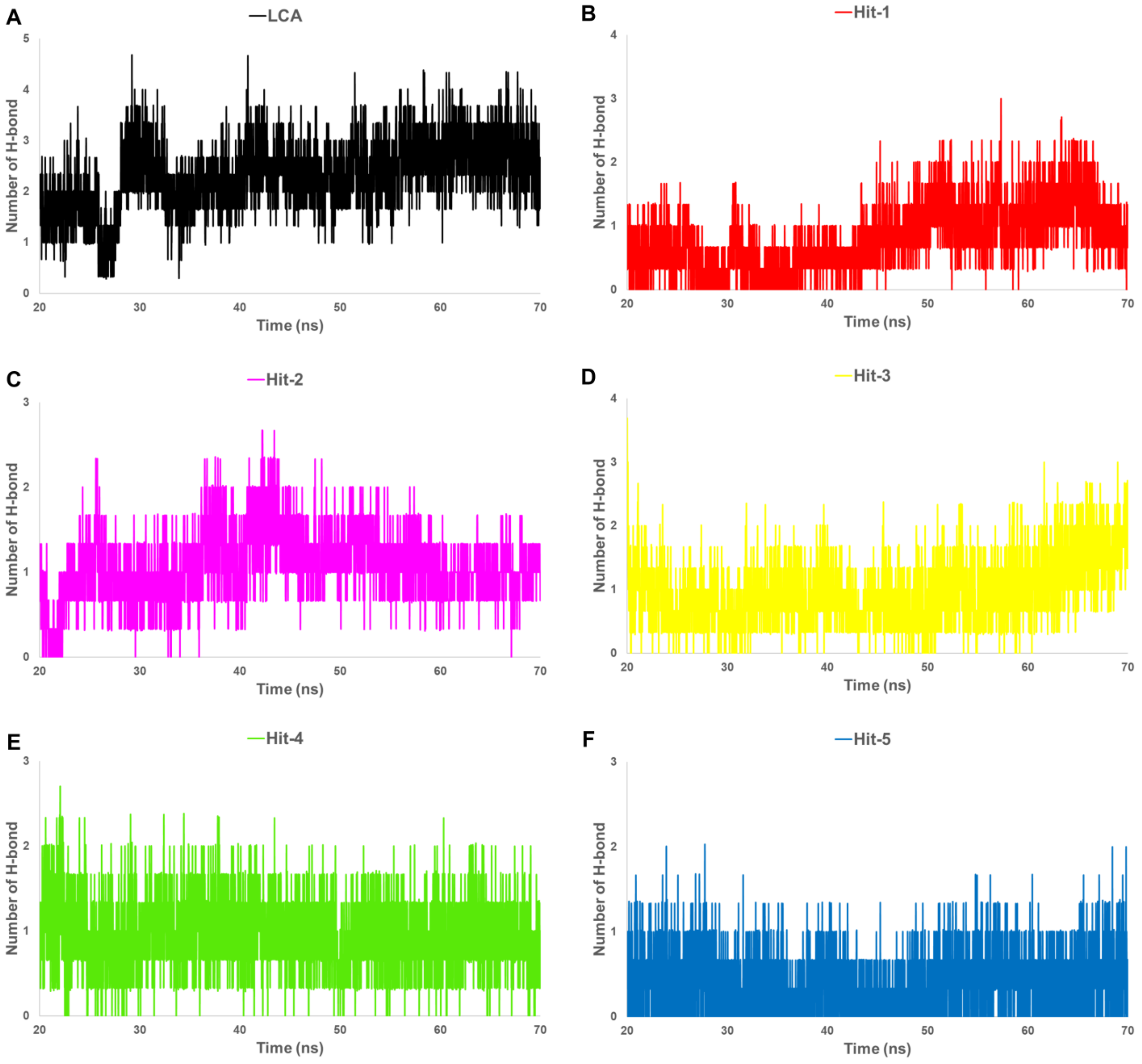

FIGURE S11. The mean number of hydrogen bond interactions formed during the MD simulation of all hits. Analysis performed at 20–70 ns.

Table S5. H-bond occupancy of hit compounds (in percentage), mean ± SD over three replicas.

Residue	Ref (LCA)	Hit-1	Hit-2	Hit-3	Hit-4	Hit-5
TRP75	0.14 ± 0.11	1.13 ± 1.60	2.93 ± 1.09	0.11 ± 0.02	1.92 ± 2.34	1.02 ± 0.71
TYR89	6.86 ± 3.87	**28.84 ± 5.76**	1.32 ± 1.43	**11.57 ± 1.26**	2.35 ± 1.69	0.17 ± 0.19
SER157	**36.93 ± 1.79**	1.27 ± 0.20	1.56 ± 1.50	**15.25 ± 1.69**	1.36 ± 0.75	0.15± 0.19
GLN158	0.00 ± 0.00	0.00 ± 0.00	0.00 ± 0.00	3.53 ± 1.65	**24.25 ± 3.76**	0.00 ± 0.00
TYR240	**27.51 ± 2.28**	0.05 ± 0.08	0.01 ± 0.01	**22.35 ± 3.38**	0.06 ± 0.08	1.87 ± 0.34
SER247	**37.86 ± 3.10**	6.75 ± 1.14	**60.66 ± 4.13**	4.64 ± 0.45	3.78 ± 1.08	0.67 ± 0.37
ALA250	0.00 ± 0.00	0.00 ± 0.00	0.23 ± 0.23	0.00 ± 0.00	0.00 ± 0.00	4.24 ± 1.41
TYR251	0.00 ± 0.00	**27.52 ± 2.45**	3.13 ± 0.79	0.06 ± 0.08	0.55 ± 0.78	4.60 ± 4.11
SER270	1.15 ± 0.19	0.53 ± 0.67	0.11 ± 0.16	0.47 ± 0.07	0.52 ± 0.74	0.03 ± 0.04

Occupancies >10% highlighted in **bold**. Analysis performed at 20–70 ns.

Table S6. List of physicochemical properties of hit compounds predicted by SwissAdme*

**Properties**	**Parameters**	**INT-777 (Reference)**	**CSC057935886 (Hit-1)**	**CSC081667704 (Hit-2)**	**CSC089939231 (Hit-3)**	**CSC083671887 (Hit-4)**	**CSC085298700 (Hit-5)**
Physicochemical properties	MW (g/mol)	450.65	366.45	454.60	437.44	387.39	360.81
	Heavy atoms	32	27	29	23	28	24
	Aromatic heavy atoms	0	14	12	6	12	6
	Rotatable bonds	5	3	3	2	3	2
	H-bond acceptors	5	4	8	2	6	4
	H-bond donors	4	2	3	4	2	2
	Molar Refractivity	128.18	103.98	122.14	100.96	109.56	92.4
	TPSA (Å²)	97.99	67.05	78.46	68.25	65.04	39.26
Lipophilicity	Log P (o/w)	3.63	3.30	1.59	1.52	2.60	3.57
Water solubility	Log S (ESOL)	Moderately soluble	Moderately soluble	Soluble	Moderately soluble	Soluble	Moderately soluble
Drug likeness	Lipinski, Violation	0	0	0	0	0	0
PAINS		No	No	No	No	No	No

* values rounded to two decimal places

Table S7. Pharmacokinetic properties and toxicity of hit compounds predicted by pkCSM*

Pharmacokinetic/ADME Properties	INT-777 Reference)	CSC057935886 (Hit-1)	CSC081667704 (Hit-2)	CSC089939231 (Hit-3)	CSC083671887 (Hit-4)	CSC085298700 (Hit-5)
Intestinal absorption (human) (% Absorbed)	57.66	92.62	90.36	64.83	89.72	89.50
P-glycoprotein substrate	No	Yes	Yes	Yes	Yes	Yes
P-glycoprotein I inhibitor	No	Yes	No	No	Yes	No
BBB permeability (log BB)	-0.68	-0.29	-1.03	-0.18	-0.20	0.26
CYP3A4 substrate	Yes	Yes	No	Yes	Yes	No
CYP3A4 inhibitor	No	No	No	No	No	No
CYP2C9 inhibitor	No	No	No	No	No	No
CYP2D6 inhibitor	No	No	No	No	No	No
Total Clearance (log ml/min/kg)	0.61	0.54	0.62	1.25	0.29	0.21
AMES toxicity	No	No	No	No	No	No
Max. tolerated dose (human) (log mg/kg/day)	-1.12	-0.75	-0.06	1.25	-0.47	-0.16
Oral Rat Acute Toxicity (LD_{50}) (mol/kg)	3.17	2.59	2.38	3.42	2.78	2.42
Oral Rat Chronic Toxicity (LOAEL) (log mg/kg_bw/day)	0.31	1.20	1.33	1.16	1.48	0.54
Hepatotoxicity	No	Yes	No	Yes	Yes	Yes
hERG I inhibitor	No	No	No	No	No	No
hERG II inhibitor	No	Yes	Yes	No	Yes	No

* values rounded to two decimal places

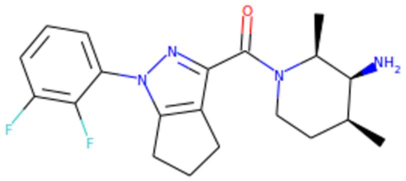

Hit-1 (CSC057935886)

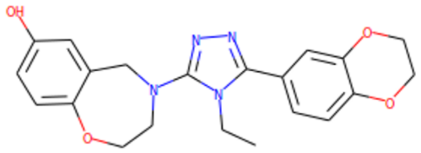

Hit-2 (CSC081667704)

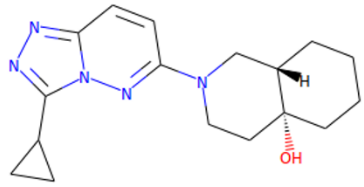

Hit-3 (CSC089939231)

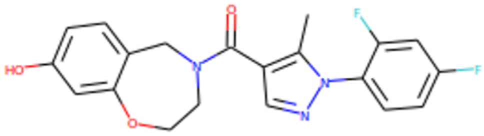

Hit-4 (CSC083671887)

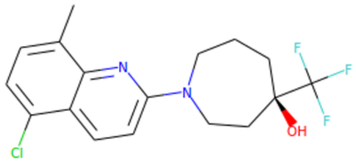

Hit-5 (CSC085298700)

FIGURE S12. The chemical structure of the hit compounds.
